# Involving men and boys in family planning: A systematic review of the effective components and characteristics of complex interventions in low‐ and middle‐income countries

**DOI:** 10.1002/cl2.1296

**Published:** 2023-01-13

**Authors:** Áine Aventin, Martin Robinson, Jennifer Hanratty, Ciara Keenan, Jayne Hamilton, Eimear Ruane McAteer, Mark Tomlinson, Mike Clarke, Friday Okonofua, Chris Bonell, Maria Lohan

**Affiliations:** ^1^ Queen's University Belfast Belfast Northern Ireland; ^2^ University College Cork Cork Ireland; ^3^ Stellenbosch University Stellenbosch South Africa; ^4^ WHARC Benin City Lagos Nigeria; ^5^ London School of Hygiene and Tropical Medicine London UK

## Abstract

**Background:**

Involving men and boys as both users and supporters of Family Planning (FP) is now considered essential for optimising maternal and child health outcomes. Evidence on how to engage men and boys to meet FP needs is therefore important.

**Objectives:**

The main objective of this review was to assess the strength of evidence in the area and uncover the effective components and critical process‐ and system‐level characteristics of successful interventions.

**Search Methods:**

We searched nine electronic databases, seven grey literature databases, organisational websites, and the reference lists of systematic reviews relating to FP. To identify process evaluations and qualitative papers associated with the included experimental studies, we used *Connected Papers* and hand searches of reference lists.

**Selection Criteria:**

Experimental and quasi‐experimental studies of behavioural and service‐level interventions involving males aged 10 years or over in low‐ and middle‐income countries to increase uptake of FP methods were included in this review.

**Data Collection and Analysis:**

Methodology was a causal chain analysis involving the development and testing of a logic model of intervention components based on stakeholder consultation and prior research. Qualitative and quantitative data relating to the evaluation studies and interventions were extracted based on the principles of ‘effectiveness‐plus’ reviews. Quantitative analysis was undertaken using r with robust variance estimation (RVE), meta‐analysis and meta‐regression. Qualitative analysis involved ‘best fit’ framework synthesis.

**Results:**

We identified 8885 potentially relevant records and included 127 in the review. Fifty‐nine (46%) of these were randomised trials, the remainder were quasi‐experimental studies with a comparison group. Fifty‐four percent of the included studies were assessed as having a high risk of bias. A meta‐analysis of 72 studies (*k* = 265) showed that the included group of interventions had statistically significantly higher odds of improving contraceptive use when compared to comparison groups (odds ratio = 1.38, confidence interval = 1.21 to 1.57, prediction interval = 0.36 to 5.31, *p* < 0.0001), but there were substantial variations in the effect sizes of the studies (*Q* = 40,647, df = 264, *p* < 0.0001; *I* ^2^ = 98%) and 73% was within cluster/study. Multi‐variate meta‐regression revealed several significant intervention delivery characteristics that moderate contraceptive use. These included community‐based educational FP interventions, interventions delivered to women as well as men and interventions delivered by trained facilitators, professionals, or peers in community, home and community, or school settings. None of the eight identified intervention components or 33 combinations of components were significant moderators of effects on contraceptive use. Qualitative analysis highlighted some of the barriers and facilitators of effective models of FP that should be considered in future practice and research.

**Authors' Conclusions:**

FP interventions that involve men and boys alongside women and girls are effective in improving uptake and use of contraceptives. The evidence suggests that policy should continue to promote the involvement of men and boys in FP in ways that also promote gender equality. Recommendations for research include the need for evaluations during conflict and disease outbreaks, and evaluation of gender transformative interventions which engage men and boys as contraceptive users and supporters in helping to achieve desired family size, fertility promotion, safe conception, as well as promoting equitable family planning decision‐making for women and girls.

## 1 PLAIN LANGUAGE SUMMARY

### 1.1 Involving men and boys in family planning is effective in increasing contraceptive use

Most family planning interventions involving men and/or boys are effective at increasing contraceptive use. Effective types of interventions include community‐based educational programmes targeting males as well as females of all ages, and programmes delivered by professionals, trained facilitators or peers.

Engaging men and boys in enhancing gender equality for women and girls as part of family planning programming was highlighted as a key strategy, but this remains an under‐used strategy.

### 1.2 What is the review about?

This systematic review of intervention evaluation studies is about how to enhance future programming with men and boys to meet needs for family planning for women and men in low‐ and middle‐income countries (LMICs).

Addressing unmet needs for family planning is a major challenge in LMICs. Addressing male involvement in family planning is also a challenge, as it is in these countries where men's control over family planning decisionmaking for women and girls is known to be greatest. It is important to involve men and boys in ways that support women's and girls' choices, as well as men's own family planning needs.

We used a novel method called causal chain analysis to focus on the content of interventions that may work better than others. This involved developing a picture of important programming components with stakeholders and testing how these components affect the impact of different interventions on family planning outcomes.

**What is the aim of this systematic review?**

This review assesses the strength of evidence of involving men and boys as users and supporters of family planning. The review also aims to uncover the effective components and critical process‐ and system‐level characteristics of successful interventions.

### 1.3 What studies are included?

We included 127 papers which examined the effectiveness of interventions that included men and/or boys in LMICs as programme participants using experimental or quasi‐experimental methods.

We also included 23 qualitative studies and process evaluations which reported why and how some programmes might have been effective.

The studies were conducted worldwide in LMICs, over half in Africa. A third of the studies were conducted on programmes that made a special effort to engage males. Less than a quarter of the studies addressed gender inequality as part of the programme.

### 1.4 What are the main findings of this review?

When considered together, the interventions included in this review were effective in increasing contraceptive use. The most effective interventions are community‐based educational programmes offered in schools, communities and homes or community facilities, and interventions involving multiple components, delivered by professionals, trained facilitators or peers to both males and females for over seven months. Brief programmes of less than three months are also effective.

Added to this, related implementation studies identified the importance of promoting gender‐equitable attitudes and social norms for women and girls among men and women at the individual, wider family, community, health service and societal level as part of family planning programming.

Some studies also emphasised structural factors such as the importance of widening women's access to education and labour markets.

### 1.5 What do these findings mean?

A wide range of family planning interventions which involve men and boys in LMICs have shown efficacy in increasing contraceptive use.

The success of family planning programmes that involve men and boys is most often measured by contraceptive use to the relative neglect of other outcomes, such as met need for family planning, equitable family planning decisionmaking, or gender equality. Our analysis indicates some promising intervention characteristics, which are more effective in promoting contraceptive use than other characteristics.

Our qualitative analysis also highlights the under‐used strategy of addressing gender equality attitudes and norms, from the individual to the structural level.

The findings of this review will be of interest to programme designers wanting to increase male engagement in family planning in gender‐equitable ways. The review can also help in measuring programme efficacy beyond contraceptive use, to also include gender equality and met family planning needs.

### 1.6 How up to date is this review?

The review authors searched for experimental evaluations in August 2020 and ‘connected’ process evaluations and qualitative studies in June 2021.

## 2 BACKGROUND

### 2.1 The problem

The World Health Organisation estimates that there are approximately 300,000 deaths per year, or 800 every day, among women and girls during childbirth or arising from pregnancy‐related complications, including unsafe abortion. Almost all (94%) of these preventable female deaths occur in low‐ and middle‐income countries (LMICs) (World Health Organisation & Press, 2019). The problem is especially acute among adolescent girls. Complications during pregnancy and childbirth are the leading cause of death for 15–19‐year‐old girls globally, with the vast majority of these occurring in LMICs (World Health Organization, 2020). Unintended and mistimed pregnancies also contribute to the burden of high infant morbidity and mortality (Kozuki et al., 2013; Say et al., 2014; A. Singh et al., 2013). Around 2.7 million new‐borns die every year in LMICs and many more suffer from diseases relating to preterm birth, being small for gestational age or malnutrition (Guttmacher, 2017).

The importance of sexual and reproductive health and rights (SRHR) as the bedrock to maternal and child health, economic growth, and the wellbeing of humanity was recognised 25 years ago in the international agreement of the International Conference on Population and Development (Starrs et al., 2018). As part of the contemporary global agenda to attain the sustainable development goals (SDGs), SRHR constitutes two targets (3.7 and 5.6), interlinking the SDGs of health and gender equality (United Nations & UN General Assembly, 2015). Family planning (FP) is a central tenet of SRHR enabling people to avoid unintended pregnancy, attain their desired number of children, and/or determine the spacing of pregnancies. Effective FP is achieved through the use of contraceptive methods, provision of safe abortion, and prevention and treatment of infertility. Worldwide, however, more than 200 million have an unmet need for family planning – wanting to avoid pregnancy but not using modern contraception and each year 25 million unsafe abortions take place (Starrs et al., 2018).

Involving men and boys in FP is increasingly recognised as essential to addressing unmet FP needs and in turn transforming maternal and child health outcomes (Croce‐Galis et al., 2014; Hardee et al., 2017; Lohan et al., 2022; Phiri et al., 2015a; Sahay et al., 2021), with programmes that adopt a focus on transforming gender inequalities for women and girls showing particular promise (Barker et al., 2007; Phiri et al., 2015b; Ruane‐McAteer et al., 2020). The underpinning rationale for involving men in FP recognises that, in many countries, men are the primary decision‐makers on family size and may control or inhibit women's use of FP as well as acknowledging that men themselves may have unmet needs in relation to FP (Nzioka & Press, 2002). In practice, ‘involving’ men and boys in FP can range from encouraging men to be supporters of autonomous FP decision‐making among women and girls, to more inclusive conceptualisations of men and boys as both supporters and users of contraceptive methods, leading change in relation to addressing unmet FP needs in their families and communities as well as meeting their own reproductive health needs (Hardee et al., 2017; Lohan, 2015; Sahay et al., 2021).

International policy debates on SRHR, and FP specifically, have therefore moved beyond the polemic of whether to involve men and boys towards the important question of *how* to involve men and boys (Ruane‐McAteer et al., 2020). The how question relates to how to involve men and boys in LMICs in ways that challenge patriarchal control over women and girls' use of FP and how to involve men as users and co‐users of FP. The question is further to address what characteristics or components of FP interventions allow men to engage with FP alongside women in ways which enhance health and gender equality for all.

### 2.2 The intervention

The review reported here included behavioural and service‐level interventions aiming to improve the uptake of FP and involve men or boys in LMICs as intervention recipients. Eligible interventions included those that aimed to increase the uptake of FP (male and/or female contraception; safe abortion and safe post‐abortion care) in order to ensure decreased unmet need for FP; avoidance of unintended or unwanted pregnancies; birth spacing (i.e., choice in relation to time period between pregnancies); and/or birth limiting (i.e., choice in relation to limiting family size). The review focuses on ‘complex’ interventions. While we recognise that some interventions, such as those with only one component, may be considered ‘simple’, following UK Medical Research Council guidelines (Craig et al., 2008) we recognise that even interventions with one component may be considered complex when they target a number of different behaviours, a variety of outcomes, or may effect behaviours via a number of different pathways.

While FP methods also include medical, surgical, and behavioural (lifestyle) interventions for addressing *infertility*, we did not examine these in the current review. The majority of fertility‐focused interventions are medical or surgical in nature (Ruane‐McAteer et al., 2019), and those that target behavioural determinants are generally focused on lifestyle changes such as reducing smoking and obesity and increasing exercise (Lan et al., 2017). In consultation with our study's https://www.qub.ac.uk/sites/involve-fp/ExpertAdvisoryGroup/, we agreed that because the theoretical basis, components, and characteristics of such interventions differ greatly from those aiming to prevent unintended pregnancy, they were outside the scope of the current study. While we agreed that should an included study address infertility alongside any of the other FP outcomes it would be eligible for inclusion, no such studies were identified.

Eligible interventions include those delivered in education, health or community settings aiming to increase capability (knowledge, skills), opportunity (access, social support) and motivation (attitudes, norms) to use FP methods via mass, small or social media information, face‐to‐face communication; health service enhancements; monetary and other incentives; and access to FP methods. The intervention approaches were grouped under the following categories:

- *Theoretical approach* (e.g., behaviour change theory; gender theory);
- *Approach to intervention design* (e.g., co‐design or co‐production);
- *Materials & procedures* (including approach to engaging men and type of contraceptive method);
- *Who provides* (e.g., health or education professionals, peers, trained facilitators);
- *Who receives* (e.g., adolescents/youth/adults; males only; males and females);
- Modes of delivery (e.g., face‐to‐face, online; individuals/couples/community);
- *Delivery setting* (e.g., home, community, educational);
- *Dose and intensity* (how much, how often, how long); and
- Tailoring, modifications, adherence or fidelity.

Interventions that vary on whether and how they address unequal gender norms in FP were also included. The modification of gender norms can be categorised on a continuum from ‘gender‐unequal/neutral’ approaches which reinforce or ignore unequal norms, roles and relations, thereby perpetuating gender‐based discrimination; to ‘gender‐sensitive/specific’ approaches, which do consider gender norms, roles and relations and/or men and women's specific needs or roles but do not seek to change gender inequalities; to ‘gender transformative’ approaches which are inclusive of gender‐sensitive and gender‐specific strategies, but also challenge gender inequalities by transforming harmful gender norms, roles and relations through programmatic strategies that foster progressive changes in power relationships between women and men (Interagency Gender Working Group, 2017; World Health Organisation, 2011).

### 2.3 How the interventions might work

This review draws upon a Causal Chain Analysis (CCA) (Kneale et al., 2015, 2018), the first step of which is to use a logic model to encapsulate how an intervention might work. The logic model is used to frame data extraction and subsequent analysis of intervention characteristics and outcomes presented (see Section 5.3). This approach addresses a common criticism of systematic reviews and meta‐analyses on the need to go beyond effectiveness analyses towards a more nuanced identification of the active ingredients of effective interventions (Pawson et al., 2005), testing of causal pathways, and identification of system‐ and process‐level barriers and facilitators to effective intervention.

The initial review logic model (Supporting Information: Appendix 1.0) was built based on: (a) a consultation with our expert advisory group; (b) a rapid review of programme theories used in FP interventions involving men and boys (Robinson et al., 2021) and (c) the research team members' own expertise of intervention design and evaluation in SRHR and involvement in prior systematic reviews conducted for the WHO on male engagement interventions in SRHR (Ruane‐McAteer et al., 2019, 2020). It provides a visual representation of how, and under what circumstances, FP interventions might work to increase uptake of FP, help people attain their desired family size and ultimately result in improvements in SRHR, maternal and child health, gender equality, quality of life and livelihoods for all.

Informed by realist interpretations of causality (Pawson et al., 2005), the logic model sets out the multiple possible pathways through which each intervention component, or combination of components, would bring about positive outcomes and change. In essence, we hypothesise that in order to positively impact maternal and child mortality and morbidity indicators, FP interventions involving men and boys first need to effect change in one or more outcomes at proximal (individual), intermediate (interpersonal, community, organisational/service) and distal (structural) levels. As illustrated in the model, changes in these outcomes follows from exposure to an intervention, although different combinations of intervention characteristics are possible and may have differential impact and may also be influenced by the characteristics of the participants and the context in which the intervention takes place. Each FP intervention will include core components as well as a set of resources and theory underlying its implementation. Further, the logic model recognises that interventions can fail to produce change because of issues relating to design or implementation processes (e.g., the intervention may not be well implemented, implementation may not trigger mechanisms or mechanisms may not generate outcomes) and, therefore, incorporates ways of understanding the success of the implementation. It also recognises that potential negative outcomes are possible for every intervention and incorporates potential indicators of these.

### 2.4 Why is it important to do this review?

To the best of our knowledge, this is the first systematic review in the field which focuses on understanding the effective characteristics and components of interventions involving men and boys in FP using causal chain analysis. Our review builds upon prior research in the field of male engagement and SRHR, which includes two WHO evidence and gap maps (EGM) two evidence and gap maps (https://srhr.org/masculinities/rhoutcomes/ https://srhr.org/masculinities/wbincome/) (srhr.org) and a systematic review of reviews of male engagement interventions across all SRHR outcomes (Ruane‐McAteer et al., 2019). There are also two previous systematic reviews of male engagement in relation to gender‐transformative SRHR interventions (Barker et al., 2007; Ruane‐McAteer et al., 2020) focusing on intervention evaluation as well as the characteristics of effective interventions.

Specifically in the field of FP, three previous reviews focus on an analysis of the characteristics and components of FP interventions, including an analysis of male involvement (Lopez et al., 2009; Mwaikambo et al., 2013; Phiri et al., 2015a). A further relevant review specifically on male engagement in FP and examining programme components was published while we were conducting the current systematic review (Sahay et al., 2021).

While our review analysis is based upon quantitative experimental evaluations of interventions, the review also includes an analysis of the available qualitative process evaluations of the interventions under study. The qualitative analysis helped to inform hypotheses of effective characteristics and components as well as our interpretation of review findings. Our review also benefits, as noted above, from consultations held with a multi‐disciplinary international advisory group based and/or working in LMICs in relation to SRHR. The findings of this review will be of benefit to programme planners and policy makers in family planning because of the wide policy interest in male engagement and the specific focus of effective programming components of interventions involving men and boys in FP The review will also help to inform the WHO's Research Priority Setting Exercise on Masculinities and SRHR https://masculinities.srhr.org/.

## 3 OBJECTIVES

The primary aim of this review was to uncover the effective components and characteristics of complex FP interventions involving men and boys in LMICs. In addressing this, we examined the following questions:

(1) What is the nature and extent of experimental evidence on engaging men and boys in FP and what gaps in research knowledge exist?
(2) What are the impacts of FP interventions involving men and boys on FP‐related outcomes?
(3) What are the effective components of interventions that achieve positive change in intended FP outcomes?
(4) What characteristics and combinations of characteristics are associated with positive FP‐related outcomes?
(5) Do outcomes vary by context and participant characteristics?
(6) Are there any unintended or adverse outcomes?
(7) What are the system‐ and process‐level barriers to and facilitators of effective models of FP involving men and boys?

## 4 METHODS

### 4.1 Criteria for considering studies for this review

#### 4.1.1 Types of study designs

As per our protocol (Aventin et al., 2021), included studies were randomised trials (individual or cluster) and quasi‐experimental studies, including quasi‐randomised trials (groups allocated using non‐random methods) and pre‐ and post‐test studies with a comparison group and, where available, their associated qualitative/mixed methods studies (e.g., formative qualitative research, process evaluations, and qualitative research exploring accounts of how the interventions work). Non‐experimental pre‐ and post‐test studies (i.e., those without a comparison group) were excluded. Mixed methods evaluations were included when the quantitative design satisfied the criteria mentioned above.

Included studies must have reported interventions or programmes implemented in countries categorised as Low Income, Lower‐Middle Income, or Upper‐Middle Income by the World Bank (World Bank, 2019) at the time the search was conducted. Studies that reported on multi‐country interventions were eligible if they met the criteria as occurring in at least one LMIC.

#### 4.1.2 Types of participants

The review focuses on FP interventions delivered in LMICs, which involved men or boys as recipients. Included studies must therefore have involved males of any age, of any sexual orientation and gender identity. While we considered outcomes for both women and men, studies were only included if boys or men received the intervention. Studies or interventions that including girls or women only were excluded.

#### 4.1.3 Types of interventions

Included interventions were FP‐focused behavioural and service‐level interventions, directly targeting or involving men or boys in LMICs. The interventions were delivered in health, education, and community settings in LMICs. Comparators included alternative interventions, usual standard care and no intervention.

#### 4.1.4 Types of outcome measures

The outcomes for this review were selected in a stakeholder‐informed logic model development phase. We consulted with FP experts to develop a review logic model (see Aventin et al., 2021) which illustrated relevant proximal and distal outcomes relating to maternal and child health and FP. While we anticipated that some outcomes featured in the review logic model, such as community, organisational and structural level outcomes and distal impacts, may not have been measured in the included studies, we aimed to examine any combination of outcomes provided.

Examples of eligible primary outcomes included: *sexual and reproductive health behaviours* (e.g., male and female contraceptive uptake and sustained use, reductions in unprotected sex, birth spacing, birth limiting); *gender equitable attitudes and behaviours* (e.g., changed attitudes and norms, decreased male‐ dominated FP decision‐making); *FP service use and engagement* (e.g., knowledge and use of FP services, use of safe abortion; support for partner engagement an increased trust in FP services); Fertility (e.g., adolescent/early pregnancy and unintended pregnancy rates). Finally, we included *met need for FP* as a key rights‐based primary outcome.

Examples of eligible secondary outcomes included: *psychosocial determinants of FP* such as knowledge, attitudes and social norms; factors relating to *relationship quality and discordance* such as couple communication and intimate partner violence; *attitudes towards FP services* including more positive attitudes towards help‐seeking in relation to FP; and *community, organisational and structural level outcomes* including gender equitable attitudes and support for FP in wider social contexts.

### 4.2 Search methods for identification of studies

As we sought to include both quantitative studies and qualitative studies in the review, the search had two phases. The first phase was a comprehensive search for randomised trials and quasi‐experimental studies. The second phase was a search for qualitative studies limited to the specific experimental evaluation studies identified in phase one to be included in the causal‐chain analysis. We used EndNote x9 software to remove duplicates in the search. We used EPPI Reviewer 4 software for data management, screening, extraction, and appraisal and further identification of duplicates with its more sensitive and configurable duplicate identification tool.

#### 4.2.1 Search strategy

##### Evaluation studies

The Phase 1 search was conducted using searches of the databases, grey literature sources and other approaches in August 2020 detailed below. The search included any available studies up until the specified dates.

1. Searches of academic literature and databases (CINAHL <August 26, 2020>, Ovid MEDLINE® ALL <August 26, 2020>, Ovid APA PsycInfo <August, Week 3 2020>, Social Science Citation Index–expanded <August 26, 2020>, Cochrane Library (including CENTRAL) <August 26, 2020>, Ovid Embase <August 25, 2020>, Scopus <August 26, 2020>, WHO Global Health Library <August 26, 2020>).
2. Searches of grey literature sources were searched using a selection of key terms on grey literature databases for any materials available to August 20, 2020 (ETHoS, ClinicalTrials.gov Register, ProQuest Dissertation & Thesis A&I, OpenGrey.eu, ELDIS.org) and searching of reports shared by relevant organisation websites (DFID, FP2020, United Nations Library/UNFPA <August 11, 2020>, IPPF <August 12, 2020>, 3ie <August 12, 2020>, USAID <August 12, 2020>, Promundo <August 11, 2020>, FHI360 <August 13, 2020>, Population Council <August 13, 2020>, Population Reference Bureau <August 20, 2020>, Institute for Reproductive Health <August 20, 2020>, Marie Stopes <August 20, 2020>). The results were hand searched for potentially relevant articles for the current review. These searches were supplemented with limited searches of the internet using Google <August 10, 2020> and project keywords.
3. Other approaches to identify eligible studies involved eliciting recommendations from disciplinary experts through the study's International Expert Advisory Group, and checking reference lists of relevant reviews identified during screening, a previous published Evidence and Gap Map (EGM) (Ruane‐McAteer et al., 2019) and a hand‐search of the Campbell Systematic Reviews journal.

##### Connected papers

The Phase 2 search was conducted using the *Connected Papers* resource (Eitan et al., 2021) to identify relevant papers by searching prior and derivative work. This resource generates ‘citation maps’ from similar or related publications based on co‐citation and text similarity assessed by machine learning across Scopus Databases. A Connected Papers graph was generated for each of the included studies in the review. The titles and abstracts of all linked results provided by the mapping tool were hand searched for relevance.

#### 4.2.2 Search limits

##### Evaluation studies

The search was not limited by publication status, date, or language of publication.

##### Connected papers

To keep the number of studies manageable, previous research by study authors not directly related to the intervention of interest and secondary analyses of data conducted outside the intervention study were not eligible for inclusion.

#### 4.2.3 Search terms

##### Evaluation studies

In Supporting Information: Appendix 2.0, we include the search strings used for our Phase 1 searches. Some of these strings were adapted from Ruane‐McAteer et al. (2018) and combined using Boolean Operator AND for terms relating to FP AND men/boys. We combined these with sensitive search filters for study design, adapted from the filter produced by Cochrane Effective Practice and Organisation of Care (2017) sample search for quasi‐experimental studies. We applied the LMIC filters developed by Cochrane EPOC group (EPOC LMIC 2020, v.3). These filters are based on the World Bank list of countries (2019, https://epoc.cochrane.org/lmic-filters). Searches were tested and adjusted as necessary to account for the unique indexing, field codes and truncation for each database.

##### Interventions

Given the very broad range of potential interventions we did not limit our searches by intervention terms in the initial stages. However, we subsequently developed this search string as follows:

(1) Search for the combination of the terms for population AND family planning AND study design AND LMIC in two databases (PsycInfo and Medline).
(2) Scan the first 200 records retrieved in each database to quickly identify studies that appear to meet our eligibility criteria (400 records screened).
(3) We used this selection of studies to develop and test a comprehensive list of intervention terms.
(4) We then screened a further selection of 200 records in each database to identify a new set of potentially eligible studies. This new set was then used to verify that the newly developed string captured the second set of potentially eligible studies and did not exclude any potentially relevant study.
(5) The first set of intervention terms failed to capture one potentially relevant study identified in step 4. The intervention term list was expanded to capture the relevant term (in this case ‘training’) and the process above was repeated once more. All relevant records were identified in the next round. We were therefore satisfied that adding intervention terms improved search specificity without adversely affecting sensitivity.

We recognise that the strategy combines five search strings, which can result in a less sensitive search. However, given the breadth of the interventions of interest, this was necessary to maximise the specificity of the search and reduce the number of irrelevant records retrieved.

### 4.3 Data collection and analysis

To ensure the most effective use of finite time and resources, subsets of the data were used for different review questions (see Table 1). While all 127 studies were included, a subset of studies reporting contraceptive use outcomes (72 studies) was used in the meta‐analysis, and a further subset of 33 studies which included interventions with a male engagement component (see Table 1 for definition) and reported contraceptive use outcomes, were used to examine impacts on intermediate outcomes. The decision to focus the bulk of the quantitative analyses on studies that reported contraceptive use outcomes was driven by, firstly, contraceptive use being the most reported FP outcome and thus yielded the most data for further analysis. Other outcomes (such as FP service use or birth spacing) were less frequently reported limiting the potential for adequately powered analysis. Secondly, resource limitations prevented dual extraction of all outcome data for all 127 studies. The decision to focus on the male engagement studies for elements of the CCA was informed by discussions among the review team and the International Advisory Group to focus attention on interventions that involved active and intentional male engagement.

**Table 1:** Intervention component names, definitions, and examples

Component name	Definition	Examples
Gender Transformative	Addressing gender inequalities and/or harmful/restrictive gender norms.	Interventions which may be inclusive of gender sensitive, and gender aware education, but also include discussion of gendered norms, or gender power and challenging of gender‐inequalities.
Information & Education	Providing information and education about FP methods, practices and outcomes.	Information provision in clinics; educational programme; informational materials dissemination.
Problem‐Solving & Skills	Activities used to increase FP related skills and competencies; Identifying barriers and facilitators of FP communication and access.	Demonstrations of correct contraceptive use; workshops and roles plays about FP communication; behaviour modelling.
Social/Peer/Mentor Support	Activities to foster social support in engaging in FP.	Outreach by male motivators and mentors; peer support; engaging religious leaders; community dialogue to support FP.
Subsidisation & Incentives	Subsidisation or free provision of FP and/or incentives to reinforce use of FP.	Free or discounted contraceptives and materials; vouchers for FP services; conditional cash transfers for use of FP.
Communication	Communication‐based strategies for improving FP outcomes.	Couples counselling; social marketing, mass media, mHealth, hotlines.
Health Service Enhancement	Programme activities intended to improve health service provision related to FP.	Training for healthcare providers; integration of FP services with other healthcare services.
Male Engagement	Programmes with a substantive aim, identified in objectives or procedures, to engage men and/or boys to impact FP outcomes.	Tailored materials and procedures to engage men and boys; purposive targeting of men and boys to effect FP behaviour change.

#### 4.3.1 Selection of studies

##### Evaluation studies

Records identified in the searches were entered into EndNote v9 and duplicates removed. Two review authors independently screened titles and abstracts to exclude studies that were obviously irrelevant. To ensure quality control, Cohen's kappa was calculated between three reviewers on the first 100 records, selected at random, and discussed to resolve any disagreements of eligibility. This process was repeated until Cohen's kappa reached 0.41 or above and we were satisfied that the screeners were making consistent decisions.

We then retrieved studies considered potentially eligible in full text. Dual independent screening of all full texts was undertaken by two review authors. The screening and quality control process outlined above was repeated with a smaller sample of 10 full texts, employing independent dual screening of records thereafter. Any disagreements were discussed with a third review author until a consensus was reached. Cohen's Kappa was once again calculated for this initial full text screening, and for the completed full text screening process ensuring adequate inter‐rater reliability (McHugh, 2012).

##### Connected papers

A citation map was generated for a sub‐set of included evaluation studies (33 studies with a male engagement component) and the connected publications were examined to identify eligible process evaluations and qualitative studies (‘connected papers’). This included investigations of the programme under evaluation conducted in intervention piloting and refinement, simultaneously with delivery, or following implementation assessing aspects of its design and delivery. This led to the identification of 8 qualitative studies and 15 process evaluations for analyses in this review. These studies related to 14 of the 33 male engagement studies.

#### 4.3.2 Data extraction and management

##### Evaluation studies

A data extraction form (Supporting Information: Appendix 3.0) was piloted on 11 studies. Following this, the only content change made to the form was the addition of the *Male Engagement* code under the *Intervention Characteristic* domain. This was added because it became clear early in the review process that this was a substantive differentiating factor in some intervention designs. No other content changes were made to the data extraction form.

Data preparation was performed using Microsoft Excel. Qualitative data extraction and synthesis was performed using annotation function in EPPI Reviewer. Outcome and numeric data were extracted in duplicate for all studies subject to causal chain analysis. This included all outcome data relating to ‘contraceptive use’ for all 127 included studies where available, and all reported FP outcome data (including contraceptive use and all other reported data for intermediate outcomes) for the 33 male engagement studies.

Due to resource constraints and the large number of eligible studies, a deviation from protocol was implemented (see 4.4 below). Dual extraction of *Study Characteristics* and *Intervention Characteristics* was conducted for 28% [*n* = 36] of included studies only and dual *Risk of Bias Appraisal* was conducted for 50% [*n* = 64] of included studies only. We evaluated the reliability of this approach and concluded that it was acceptable in accordance with accepted standards (Landis & Koch, 1977; McHugh, 2012), thus the extraction of *Study Characteristics*, *Intervention Characteristics*, and *Risk of Bias Appraisal* by one review author was implemented for the remaining studies.

As the characteristics and components of interventions were a central feature of this review, care was taken to extract and code this data according to the a priori defined categories outlined in the initial review logic model (Supporting Information: Appendix 1.0), whilst also permitting the inclusion and/or refinement of component names and definitions as coding proceeded. The final component names and definitions used for coding and reporting are included in Table 1.

All studies coded as containing a ‘male engagement’ component (see definition in Table 1) were assessed independently by two review authors to verify the presence of this component. Disagreements about the presence of this component in three studies were resolved by discussion with a third review author.

##### Connected papers

Qualitative data extractions were done for the 23 connected papers and, where reported, the subset of 33 male engagement evaluation studies. Extraction was conducted by one review author in EPPI and checked by a second author. Data constituted verbatim sections of text describing:

(1) full or partial causal‐chain descriptions, whereby authors explain or hypothesise what caused an outcome and under which circumstances;
(2) reflections of the original authors on how specific elements of an intervention worked/might have worked; and
(3) statements on how specific mediators, moderators, and system‐ and process‐level barriers and facilitators impacted/may have impacted on outcomes.

#### 4.3.3 Assessment of risk of bias in included studies

##### Evaluation studies

Assessment of methodological quality and risk for bias in randomised trials was conducted using the Cochrane Risk of Bias tool for Randomised Controlled Trials (RoB 1) (Higgins et al., 2011). This is a standard tool, which takes the forms of a series of questions about the randomisation procedures and blinding. Non‐randomised studies were coded using ROBINS‐I (Sterne et al., 2016). As noted above, dual risk of bias appraisal was conducted for 50% [*n* = 64] of included evaluation studies. We evaluated the reliability of this approach and concluded that it was acceptable in accordance with accepted standards (Landis & Koch, 1977; McHugh, 2012), and risk of bias appraisal by one review author was implemented for the remaining studies.

##### Connected papers

Qualitative studies were coded by one review author using the Jimenez and colleagues (Jimenez et al., 2018) critical appraisal tool and quantitative process evaluation studies using the EPPI‐Centre & EPPI‐Centre Social Science Research Unit (2003). These codes were checked by a second review author. We did not exclude any studies from the review on the basis of quality, rather, we conducted a sensitivity analysis exploring the impact of including low quality studies on the overall findings.

#### 4.3.4 Criteria for determination of independent findings

There were sufficient eligible studies reporting multiple and dependent effect sizes (i.e., occurring in more than 20 eligible studies) so robust variance estimation (RVE) was employed to account for dependency in the data. This technique calculates the variance between effect sizes to give a quantifiable standard error for the variable of interest. It has been shown to calculate correct results with a minimum of 20–30 individual studies (Hedges et al., 2010), although it performs better with more studies.

#### 4.3.5 Measures of treatment effect

Outcomes were typically reported as dichotomous data so meta‐analysis was conducted using odds ratio (OR), with a random effects model. We focused our analysis on contraceptive use because this was the most measured outcome across all studies.

#### 4.3.6 Unit of analysis issues

##### Multiple intervention groups

We used RVE to account for dependencies in the data and to allow us to make use of multiple effect sizes reported in single studies.

##### Multiple interventions per individual

We coded each study according to intervention components. We used meta‐regression to assess the effectiveness of individual and combined intervention components.

#### 4.3.7 Dealing with missing data

Of the 127 included studies, 12 study reports did not contain sufficient data to allow calculation of effect size estimates for the primary outcome of our analyses, contraceptive use. When appropriate, we contacted the original authors to request necessary summary data, such as means and standard deviations or standard errors. Where no information was provided, the study was not included in the meta‐analysis and was included in the narrative synthesis only. We were unable to retrieve information for 40 effect sizes across 12 included studies. These studies were included in the review but excluded from the meta‐analysis.

#### 4.3.8 Assessment of heterogeneity

Heterogeneity was assessed first through visual inspection of forest plots and checking for overlap of confidence intervals and second through the *Q*, *I* ^2^ and Tau^2^ statistics. Investigation of the source of heterogeneity is addressed in data synthesis section.

#### 4.3.9 Assessment of reporting biases

We assessed small study bias (such as publication bias) using a regression test for funnel plot asymmetry (Egger et al., 1997). The model used was a weighted regression with multiplicative dispersion using sampling variance as predictor.

To ensure robustness of the review and to account for individual studies that appear to exert an undue influence on findings, process sensitivity analysis was carried out on domains relating to the quality of the included studies (Cooper, 2016).

#### 4.3.10 Data synthesis

We adopted a Causal Chain Analysis (CCA) (Ivers et al., 2014; Kneale et al., 2018; Tanner‐Smith & Grant, 2018) approach to data synthesis. The logic model was tested using appropriate meta‐analytic techniques combined with findings from narrative synthesis of evaluation study findings and qualitative analysis of connected papers. The process involved the following:

(1) **Multivariate pairwise meta‐analysis** to assess the overall effectiveness of the interventions on reported FP outcomes;
(2) **Meta‐regression** to assess the impact of multiple intervention components and characteristics on FP outcomes; and
(3) **Narrative synthesis** involving the identification of characteristics and components of included interventions and **‘best‐fit’ framework synthesis** of connected qualitative studies and process evaluations to identify barriers and facilitators to effective models of FP.

As noted, different subsets of the data were used for the review questions (see Table 2). All 127 studies were included in the narrative synthesis relating to review questions 1 and 6. The subset of 72 studies are those that report contraceptive use outcome data and had outliers removed and this subset was used for questions 2–5. A further subset was created of male engagement studies. This subset of 33 studies are those that report contraceptive use outcome data, had outliers removed, and included a ‘male engagement’ component (i.e., explicitly stated an intention or practice of engaging men/boys either through their objectives or tailoring their practice for males in order to impact FP outcomes). These studies were used in the analysis in questions 2. The analytic approach for each of our objectives is summarised in Table 2.

**Table 2:** Summary of analysis procedures used for each review question

Review question	Analysis approach
1. What is the nature and extent of experimental evidence on engaging men and boys in FP and what gaps in research knowledge exist?	Summary statistics and narrative synthesis for 127 studies.
2. What are the impacts of FP interventions involving men and boys on FP‐related outcomes?	Multivariate pairwise meta‐analysis of the effect of interventions on ‘contraceptive use’ compared to comparisons for 72 studies (see Supporting Information: Appendix 7.1).
	Multivariate pairwise meta‐analysis of the effect of interventions on intermediate FP outcomes^a^ compared to comparisons for 33^b^ male engagement studies (see Supporting Information: Appendix 7.2).
3. What are the effective components of interventions that achieve positive change in intended FP outcomes?	Meta‐regression to estimate variance accounted for by the identified intervention components and combinations of components for 72 studies.
4. What characteristics and combinations of characteristics are associated with positive FP‐related outcomes?	Meta‐regression on extrinsic (year of publication); methodological (study design); and substantive (intervention design, dosage, intervention setting; intervention theory of change; who delivers) variables for 72 studies.
5. Do outcomes vary by context and participant characteristics?	Multivariate meta‐analysis of dependent effect sizes with robust variance estimation on characteristics of context (region) and participants (age and sex) for 72 studies.
6. What adverse effects were reported?	Narrative synthesis of any reported adverse effects in 127 studies and qualitative synthesis of 23 connected papers (See Supporting Information: Appendix 7.3).
7. What are the system‐ and process‐level barriers to and enablers of effective models of FP involving men and boys?	Qualitative synthesis using a ‘best‐fit’ framework synthesis approach for 23 connected papers (11 connected qualitative studies and 12 connected process evaluations).

^a^ Intermediate FP outcomes: attitudes to FP services; contraceptive attitudes; contraceptive knowledge; FP communication; gender equitable behaviours and beliefs; joint FP decision‐making; and FP service use.

^b^ Thirty‐four male engagement studies less removal of one study with outliers.

#### 4.3.11 Approach to meta‐analysis

Given the diverse range of interventions included in this review, random effects models, using RVE, were used as the basis for meta‐analysis. The analyses were conducted using *r* and the range of commands externally developed to conduct meta‐analysis with r including *metafor* and *clubSandwich* (Megha Joshi, 2022; Michael Kossmeier, 2020; Pustejovsky & Tipton, 2018; Viechtbauer, 2010).

#### 4.3.12 Main effects

The main effects analysis, synthesising the evidence on the effects of the interventions was undertaken using multivariate pair‐wise meta‐analysis outlined above for each outcome in turn.

#### 4.3.13 Sensitivity analysis

For each outcome, the following sensitivity analyses was undertaken to assess whether there were potential influences relating to studies that appear to exert an undue influence on findings. We used meta‐regression to assess the impact of:

- Year study was conducted
- Study design (cluster‐RCT, RCT, Quasi‐experimental)

We did not conduct sensitivity analysis on study risk of bias due to the mixture of RCTs and non‐RCTs.

#### 4.3.14 Subgroup analysis and investigation of heterogeneity

The complexity of the logic model means that there were many possible subgroup analyses and meta‐regressions to assess the differential effects in relation to the components of interventions, characteristics of the intervention delivery, population of interest and context. Using robust variance estimates, we conducted analysis for the following:

- Geographical region
- Who delivered the intervention (peers, professionals, trained facilitators, etc.)
- Intervention dosage
- Intervention design
- Population included (males only or males and females)
- Intervention setting
- Age of participants (adolescents, adults, both)
- Presence or absence of a theory of behaviour change

This exploratory analysis used single‐variable, no intercept model and we did meta regression using residual maximum likelihood (REML). The meta‐regression model was a no‐intercept effect size model with dummy codes for each included variable.

#### 4.3.15 Treatment of qualitative data

Qualitative data extracted from the 23 connected papers (15 process evaluations and 8 qualitative studies) were analysed using a ‘best‐fit’ framework synthesis approach (Booth & Carroll, 2015; Carroll et al., 2013). Where possible, qualitative data was also extracted from the subset of 33 male engagement studies. The a priori framework used to code the data constituted categories from the review logic model (Supporting Information: Appendix 1.0). One author coded the data deductively using the a priori framework and subsequently conducted thematic analysis. Inductive, thematic analysis techniques were used for data that could not be coded under existing categories. Codes and resulting categories were checked by a second author and any differences in opinion were resolved through discussion. The synthesis findings were used to inform decision‐making in relation to the quantitative synthesis and to help explain and provide additional evidence for the outcome patterns reported in the quantitative synthesis. Information from the critical appraisal tools was not used to exclude studies but a sensitivity analysis was conducted by excluding low‐quality studies and to test the impact of these exclusions on the overall synthesis of findings. Conclusions were integrated at the end of the review process in the conclusion and discussion section and used to inform a revised version of the review logic model.

### 4.4 Deviations from protocol

#### 4.4.1 Data extraction

The published review protocol (Aventin et al., 2021) specified that data extraction and risk of bias appraisal would be carried out in full by two reviewers independently. Due to the large number of included studies (127) and resource constraints, this was not possible. We have upheld standards in line with the methodological guidance specified by the Campbell MECCIER guidelines (Methods Group of the Campbell Collaboration, 2019) in light of these constraints. One review author completed data extraction and risk of bias appraisals for all 127 included studies. In total, 36 (28.1%) included studies were subject to dual data extraction of intervention characteristics, and 64 (50%) risk of bias appraisals were done by another member of the review team independently. To ensure accurate extraction of numerical data, contraceptive use outcome and numeric data were extracted in duplicate for all 72 studies subject to quantitative analysis and checked by an experienced methodologist who conducted the analyses. Intermediate outcome and numerical data (extracted only for the 33 male engagement studies) was extracted by an experienced research assistant and checked by one review author and an experienced methodologist who conducted the analysis.

Duplicate extraction and appraisal were subject to evaluation by the review team to ensure consistent decision‐making by a single reviewer. To assess inter‐rater agreement and provide a measure of internal validity, we present the kappa statistic, *κ*. Generally, Cohen's kappa is used most often as it determines agreement between reviewer A and reviewer B (Landis & Koch, 1977) but the Fleiss kappa statistic may be used where there are multiple reviewers extracting the same data (Fleiss, 1971). The kappa statistic is preferable to reporting percent agreement, as the possibility of agreement occurring by chance is included in the equation. We used this to establish internal consistency across the team. This measure was checked using the irr package in R. Calculation of Fleiss' kappa was determined to be favourable for data extraction (Percent Agreement = 94.6, *κ* = 0.70) and risk of bias tools (Percent Agreement = 87.4, *κ* = 0.56).

Reliability of data extraction was deemed acceptable in accordance with accepted standards (Landis & Koch, 1977; McHugh, 2012), thus the extraction of Study Characteristics, Intervention Characteristics, and Risk of Bias Appraisal (see Supporting Information: Appendix 3.0, Data Extraction Form) by one reviewer was accepted.

#### 4.4.2 Analysis

Although it had been our intention to conduct the full causal chain analysis on all included studies, our inclusion criteria led to the inclusion of a large number of studies (127). Time constraints led us to focus our resources of dual extraction and data analysis of intermediate outcome data for a subset of 44 male engagement studies only, 33 of which were included in the meta‐analysis as these included a contraceptive use outcome. These studies were used to answer review Q2 regarding the effectiveness of interventions on intermediate FP outcomes and they were also used as the basis for selecting the 23 connected papers (i.e., the connected papers relate to the 33 male engagement evaluations studies).

Finally, in a deviation from our per‐protocol analysis we did not conduct analysis separately for different follow‐up times as planned. Instead, we used RVE to allow us to combine multiple effect sizes on the same outcome from each study while accounting for dependency in the data. We did not conduct separate analysis where the same outcome construct was measured but across multiple time domains, such as through the collection of both post‐test and further follow‐up data.

## 5 RESULTS

### 5.1 Description of studies

#### 5.1.1 Results of the search

**Figure 1:**
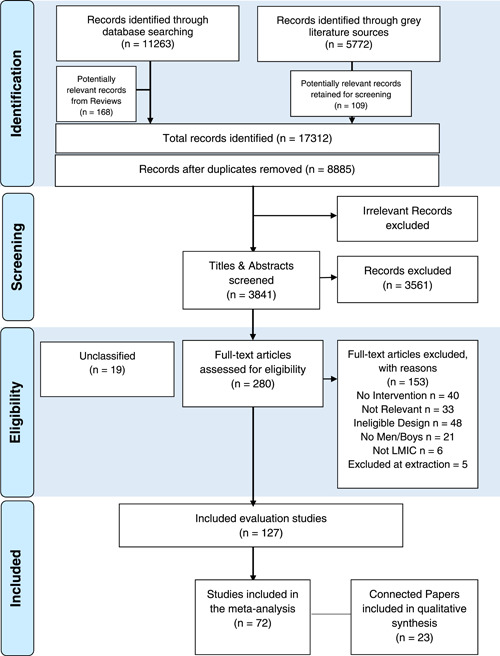
INVOLVE_FP review PRISMA flow diagram

Figure 1 shows the results of the search process. A total of 8885 potentially relevant records were identified from our academic and grey literature searches, after excluding 8318 duplicates. The 8885 articles included 168 records that were identified from hand searches of the reference lists of 89 review articles. All 8885 records were screened for relevancy based on their title and abstract and, of these, 5044 were excluded because they were obviously irrelevant to this review (e.g., records related to animal studies, studies of infant mortality, health interventions explicitly not related to FP behaviours such as child nutrition and smoking cessation, reporting of national demographic and health surveys). The titles and abstracts of the remaining 3841 records were screened according to the following criteria:

1. Related to a psycho‐social or behavioural FP intervention
2. Related to a Randomised Controlled Trial or Quasi‐Experimental Design
3. Involved males in intervention delivery
4. Conducted in a Low‐ or Middle‐Income Country(ies)

These criteria were applied in sequential order for the purposes of exclusion and inclusion of records in title and abstract screening and led to the following exclusions:

- not related to a psycho‐social or behavioural FP intervention (*n* = 2864, 80.5%) (e.g., surveys of family planning attitudes or practices, commentary on family planning, an intervention unrelated to family planning behaviours),
- ineligible study design (*n* = 633, 17.8%) (e.g., pre‐ post‐intervention designs, lack of a comparison group, intervention protocol or development paper, review of interventions),
- did not involve men or boys in intervention delivery (*n* = 55, 1.6%),
- not conducted in a LMIC (*n* = 5, 0.01%),
- unavailable publication abstract or full text, thus awaiting classification (*n* = 5, 0.01%).

Following title and abstract screening of identified studies, 280 records were subject to full‐text screening. In assessing studies for eligibility at this stage, the same four criteria were applied to the records marked for inclusion at the title and abstract screening stage. This led to the exclusion of a further 147 records for the following reasons:

- Did not evaluate an intervention (*n* = 40, 27.2%)
- Did not evaluate a relevant intervention (*n* = 32, 21.8%)
- Ineligible study design, i.e., no comparison group (*n* = 48, 32.7%)
- Did not deliver intervention to men or boys (*n* = 21, 14.3%)
- Was not conducted in a LMIC (*n* = 6, 0.4%)

Five records were removed following closer examination during data extraction for the following reasons, which are in line with the eligibility criteria for this review: lacking a comparison group exposed to a different or no intervention (Baochang et al., 1998; Nabaggala et al., 2019); intervention content related to HIV prevention exclusively (Harvey et al., 2000; Vernon et al., 1990); intervention delivered to females only despite appearing to encourage male involvement (Jahanfar et al., 2005). The review team was unable to acquire abstract or full‐text resources for a total of 19 records, meaning these were labelled as ‘*Awaiting Classification*’ and did not advance to eligibility assessment or inclusion.

#### 5.1.2 Included studies

A total of 127 evaluation studies were included in the review. Of these, 106 were identified from database and grey literature searches, 18 from review forward searching, and 3 from searches of the EGM (Ruane‐McAteer et al., 2019) (see Section 4.2.1). As noted, a total of 23 ‘connected’ process evaluations and qualitative papers relating to 14 of the included experimental evaluation studies were also included.

##### Review question 1: What is the nature and extent of experimental evidence on engaging men and boys in FP?

This section reports findings relating to Review Question 1 on the nature and extent of experimental evidence on engaging men and boys in FP. An overview of the characteristics of all included studies (*n* = 127) (see Table 3) is followed by a summary of characteristics of the 44 studies that had a male engagement component. Finally, the characteristics of the 23 connected papers are outlined. Supporting Information: Appendix 4.0 provides detailed study information for the 127 studies included in the review. Tables 3 and 5 provide summary statistics for all included studies and male engagement studies, respectively.

**Table 3:** Key summary statistics for all included studies (*n* = 127)

Characteristic	*N*	%	Characteristic	*N*	%
*Region*			*Intervention design*		
Asia	37	29	Community Based Educational	101	80
Africa	67	53	Maternal & Child Health Programme	5	4
America	25	20	Contraceptive Counselling	21	16
*Study design*			*Components included*		
RCT	51	40	Information & Education	123	97
QE	68	54	Social/Peer Mentor Support	58	46
cRCT	8	6	Communication	51	40
*Publication type*			Male engagement	44	35
Journal Article	103	81	Service Enhancement	41	32
Report	14	11	Problem Solving & Skills	35	28
Thesis	5	4	Subsidisation & Incentives	34	27
Presentation Abstract	1	1	Gender Transformative	29	23
*Year of publication*			*Number of components*		
1965–1985	8	6	1	10	8
1986–2011	74	58	2 to 4	90	71
2012–2019	45	35	5 to 7	27	21
*Intervention recipients*			*Dosage*		
Men and Women	118	93	<3 months	42	33
Men only	9	7	3–6 months	20	16
Adolescents only	39	31	7–12 months	24	19
Adults only	31	24	>12 months	38	30
Adolescents and adults	57	45	*Intervention setting*		
*Mode of delivery*			Community only	36	28
Individuals only	18	14	Home only	6	5
Couples only	3	2	Healthcare only	25	20
Groups only	45	35	Schools/Universities only	37	29
Media only	3	2	Mixed settings	21	17
Mixed modes	54	43	Not specified	2	1
Not specified	4	3			
*Intervention provider*			*Outcomes reported*		
Professionals only	38	30	Contraceptive use	72	57
Peers only	12	10	Pregnancy, pregnancy timing and desired family size	36	28
Trained Facilitators only	31	24	Contraceptive attitudes	49	39
Mhealth only	8	6	Contraceptive knowledge	52	41
Media only	2	1	Communication about FP	29	23
Mixed providers	25	20	Service use	31	24
Not specified	11	9	Equitable decision‐making about FP	12	9

**Table 4:** Key summary statistics for male engagement studies (*n* = 44)

Characteristic	*N*	%	Characteristic	*N*	%
*Region*			*Intervention design*		
Asia	16	36	Community Based Educational	26	59
Africa	24	55	Maternal & Child Health Programme	3	7
America	4	9	Contraceptive Counselling	15	34
*Study design*			*Components included*		
RCT	19	52	Information & Education	42	95
QE	21	48	Social/Peer Mentor Support	19	43
cRCT	4		Communication	20	45
*Publication Type*			Male Engagement	44	100
Journal Article	33	75	Health Service Enhancement	17	39
Report	6	14	Problem Solving & Skills	8	18
Thesis	2	5	Subsidisation & Incentives	9	20
Presentation Abstract	3	7	Gender Transformative	15	34
*Year of publication*			*Number of components*		
1965–1985	3	7	2	6	14
1986–2011	20	45	3 to 4	21	48
2012–2019	21	48	5 to 6	17	39
*Intervention recipients*			*Dosage*		
Men and Women	37	84	<3 months	10	23
Men only	7	16	3–6 months	10	23
Adolescents only	2	5	7–12 months	8	18
Adults only	19	43	>12 months	11	25
Adolescents and adults	23	52	Mixed dosage	4	9
*Mode of delivery*			Not specified	1	2
Individuals only	9	20	*Intervention setting*		
Couples only	1	2	Community only	14	32
Groups only	11	25	Home only	4	9
Mixed modes	22	50	Healthcare only	14	32
Not specified	1	2	Schools/Universities only	3	7
			Mixed settings	9	20
*Intervention provider*			*Outcomes reported*		
Professionals only	17	39	Contraceptive use	34	77
Peers only	4	9	Pregnancy, pregnancy timing & desired family size	21	48
Trained Facilitators only	12	27	Contraceptive attitudes	19	43
Mhealth only	3	7	Contraceptive knowledge	16	36
Mixed providers	7	16	Communication about FP	11	25
Not specified	1	2	FP Service use	11	25
			Joint FP decision‐making	8	18

**Table 5:** Risk of bias in connected papers

First author and year	Study design	Overall risk of bias judgement
Ahmed et al. (2015)	Process Evaluation	Moderate
Akhter et al. (1993)	Process Evaluation	Moderate
Baqui et al. (2018)	Process Evaluation	Low
Bertrand et al. (1982)	Process Evaluation	Moderate
Daniele (2017)	Process Evaluation	Low
Doyle et al. (2011)	Process Evaluation	Moderate
Harrington (2017a)	Process Evaluation	Moderate
Harrington (2017b)	Process Evaluation	Moderate
Khan et al. (2008)	Process Evaluation	Moderate
Mantell et al. (2006)	Process Evaluation	Low
McCarthy et al. (2018)	Process Evaluation	Moderate
McCarthy et al. (2018)	Process Evaluation	Moderate
Ngure et al. (2012)	Process Evaluation	Moderate
Ross et al. (2007)	Process Evaluation	Moderate
Turan (1997)	Process Evaluation	Moderate
Cooper et al. (2014)	Qualitative Study	Moderate
Ghule et al. (2015)	Qualitative Study	Moderate
Harrington et al. (2016)	Qualitative Study	Low
Harrington et al. (2019)	Qualitative Study	Moderate
Hartmann et al. (2012)	Qualitative Study	Moderate
Jewkes et al. (2008)	Qualitative Study	Moderate
McCarthy (2019)	Qualitative Study	High
Nair et al. (2019)	Qualitative Study	Moderate

###### Study characteristics all evaluation studies (n = 127)

####### Year of publication, participants and study design

**Figure 2:**
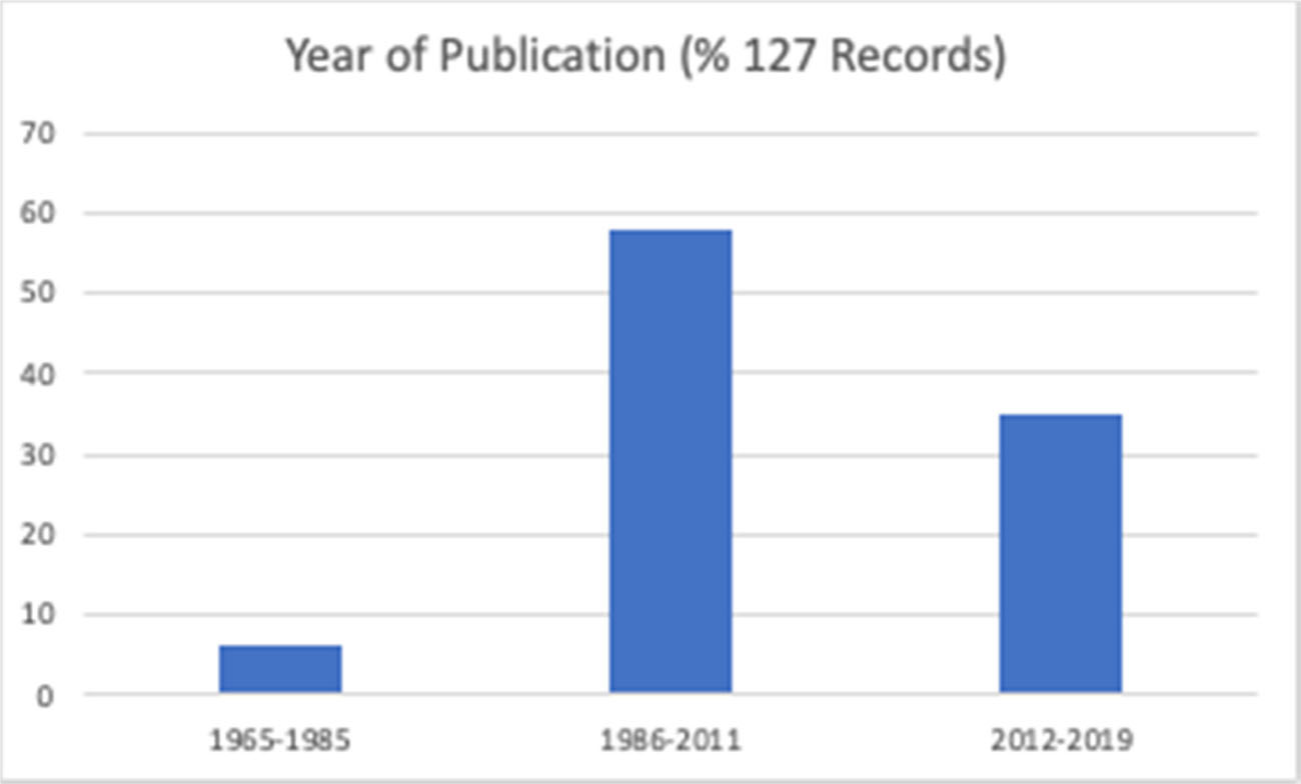
Study design (all included studies)

**Figure 3:**
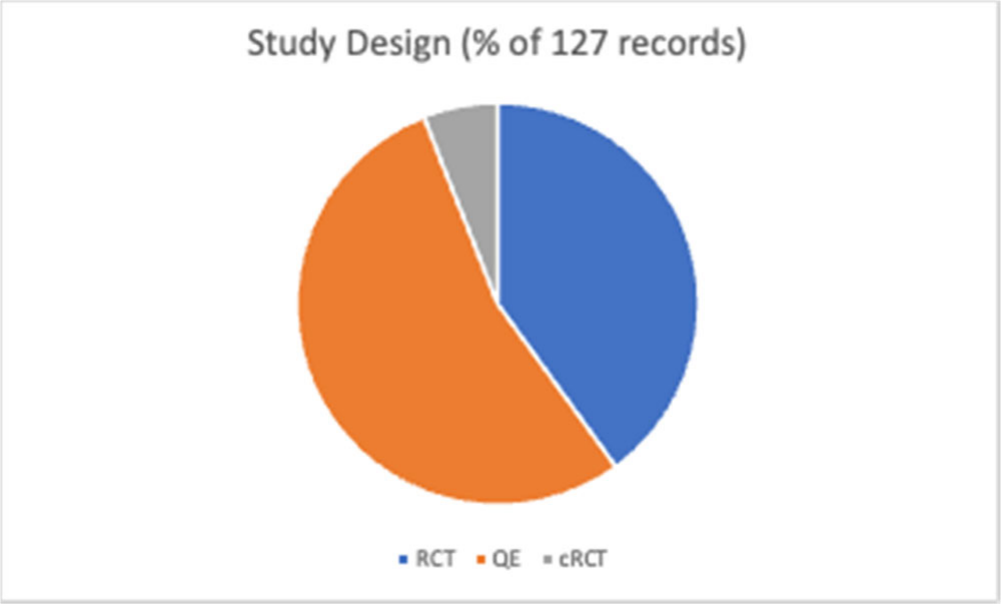
Study design (all included studies)

The review includes studies published between 1965 and 2019 (Figure 2), with a third of these (*n* = 43) published since 2012. The studies included a total of 491,365 participants. Fifty‐one (40.2%) of the included studies were randomised trials (RCTs), eight (3.1%) were cluster randomised trials (cRCTs) and 68 (53.5%) were quasi‐experimental studies (Figure 3).

####### Study location

**Figure 4:**
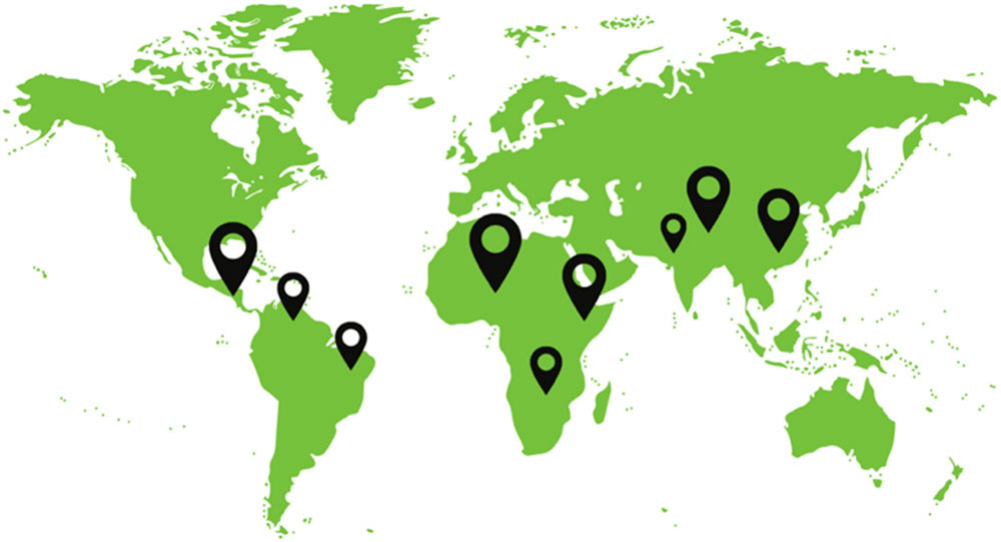
Study locations

The included studies provide a global scope of reported experimental evaluations of FP programming with men and boys in LMICs. Figure 4 illustrates the geographic dispersion of study locations. Over half of studies (*n* = 67) took place in Africa. Among the most common study sites were *Kenya* (*n* = 10), *South Africa* (*n* = 7), *Nigeria* (*n* = 6). This was followed by Asia (*n* = 37), with *China* (*n* = 12), *India* (*n* = 7), *Bangladesh* (*n* = 6), and *Vietnam* (*n* = 4) the most frequently reported study locations. Around 20% (*n* = 25) of studies took place in the Americas. Most common study sites were *Mexico* (*n* = 7), *Brazil* (*n* = 3), *Guatemala* (*n* = 3), and *Colombia* (*n* = 3).

####### Intervention design & components

**Figure 5:**
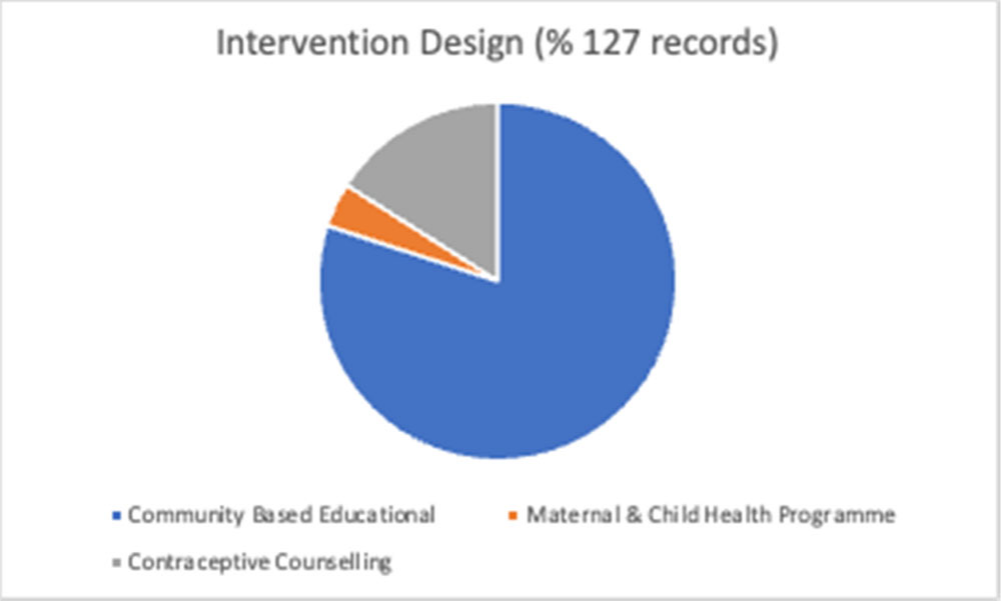
Intervention design (all included studies)

The studies involved three broad categories in relation to the central design of the intervention—community based educational interventions (*n* = 101), contraceptive counselling (*n* = 21), and maternal and child health programmes (*n* = 5) (Figure 5). Interventions were delivered across a variety of settings including schools and universities (*n* = 37), the community (*n* = 36), healthcare settings (*n* = 25), homes (*n* = 6), and some combination of these (*n* = 21).

**Figure 6:**
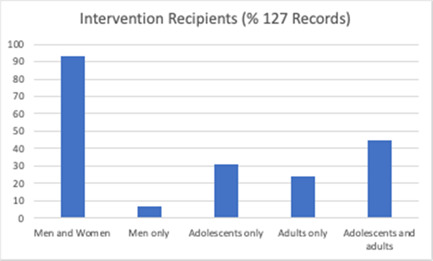
Intervention recipients (all included studies)

Figure 6 shows that intervention recipients were most often both men and women (*n* = 118), but some were delivered to men only (*n* = 9). The interventions were delivered to adolescents (age 10–19) (*n* = 39), adults (*n* = 31) or both (*n* = 57). Participants received the intervention as individuals (*n* = 18), couples only (*n* = 3), groups (*n* = 45), or via a combination of different modes of delivery (*n* = 54).

**Figure 7:**
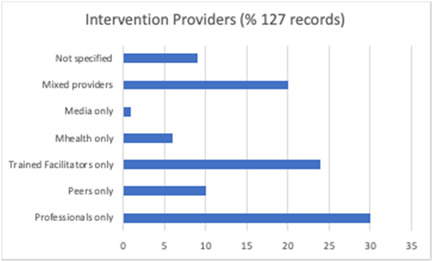
Intervention providers (all included studies)

Several different intervention providers were noted, and these included: professionals such as teachers and nurses (*n* = 38), trained facilitators (*n* = 31), trained peers (*n* = 12), mHealth (*n* = 8), or a combination of these (*n* = 25). Some mHealth (*n* = 8) and digital/mass media (*n* = 2) interventions were delivered remotely with no human contact involved (Figure 7).

**Figure 8:**
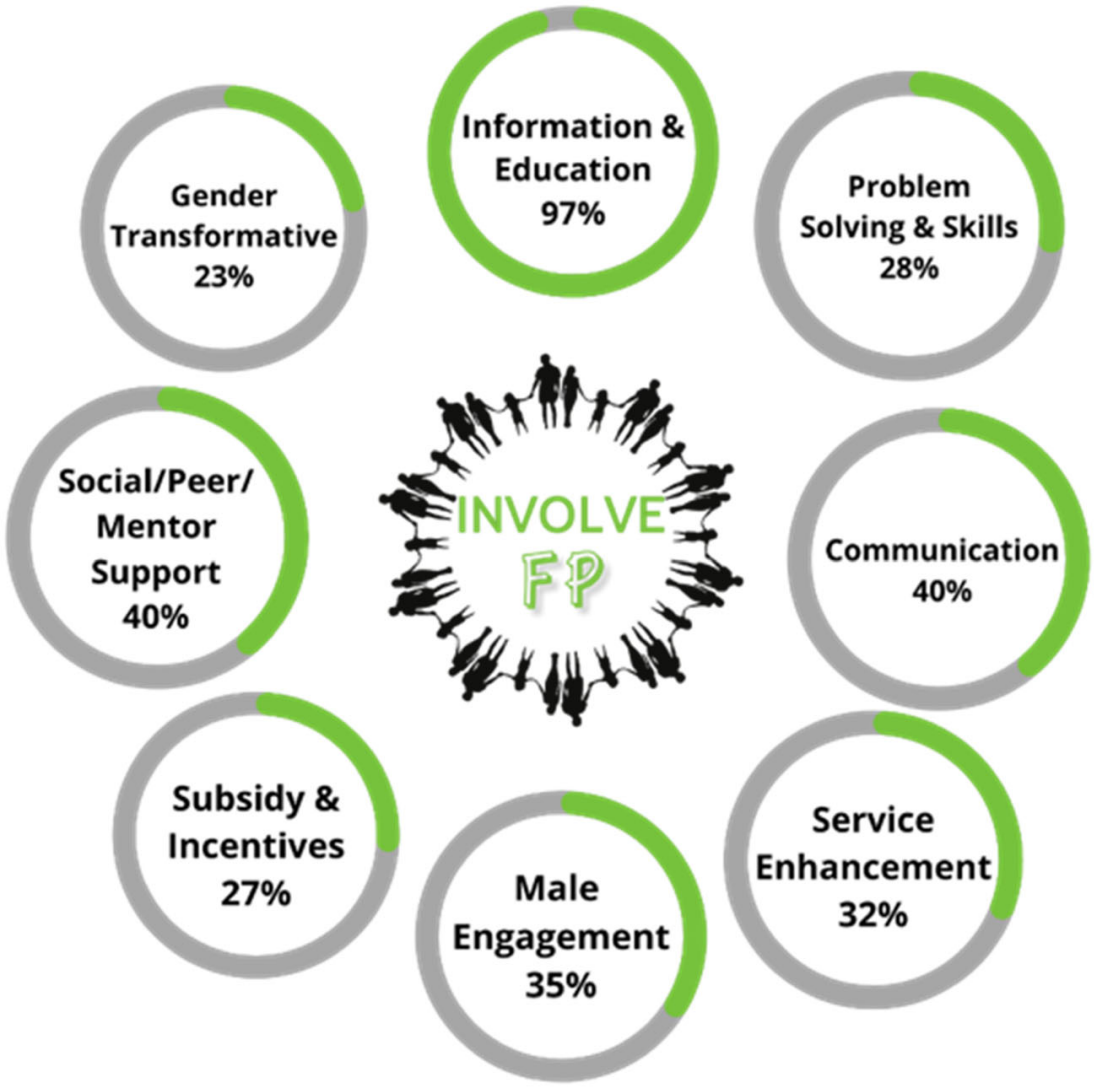
Percentage of intervention components (all included studies)

As illustrated in Figure 8, the most common intervention component or strategy was the *Provision of Information or Education* about FP and contraception, with 123 (97%) of studies incorporating this. Other commonly included components were *social/peer mentor support* (*n* = 58, 46%) and *Communication* (*n* = 51, 40%). Less common were *Problem‐Solving and Skills building* (*n* = 35, 28%), *subsidisation and incentives* (*n* = 34, 27%), and gender transformative components (*n* = 29, 23%). While all interventions in the included studies involved men or boys in their delivery, only 35% (*n* = 44) of included studies substantively incorporated intervention components designed to *engage males* with through their objectives or tailored delivery for this group.

Those interventions categorised with the *Male Engagement* component (*N* = 44) were those that included substantive engagement of men and boys evidenced either through their objectives or tailoring their practice for males purposefully. Examples of this include:

- Explicit targeting of husbands for counselling to increase acceptance of female family planning methods (Amatya et al., 1994; Fisek & Sumbuloglu, 1978; Ha et al., 2005a).
- Male promoters used to disseminate information to males and increase acceptability of male family planning methods and participation (Bertrand et al., 1982; Shattuck et al., 2011).
- Tailored messaging and communication for the purpose of involving men and boys (El‐Khoury et al., 2016; Fleming et al., 2018; Mantell et al., 2017; Sebastian et al., 2010).
- Intervention objectives specifically and exclusively targeting men (Exner et al., 2009; Sahip & Turan, 2007; Shattuck et al., 2011).

**Figure 9:**
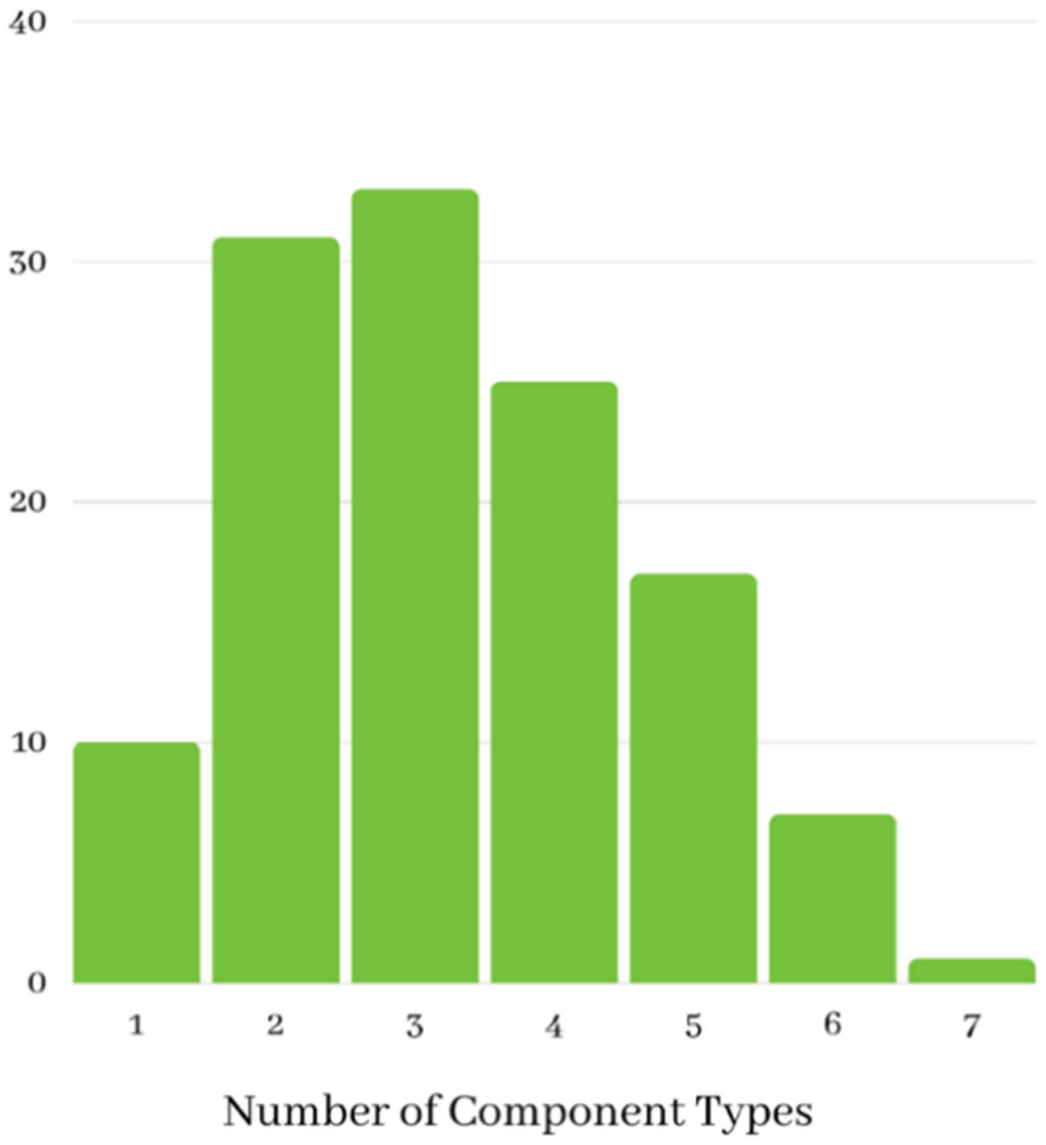
Number of different components (all included studies)

**Figure 10:**
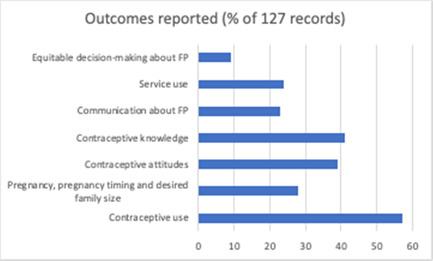
Outcomes reported (all included studies)

**Figure 11:**
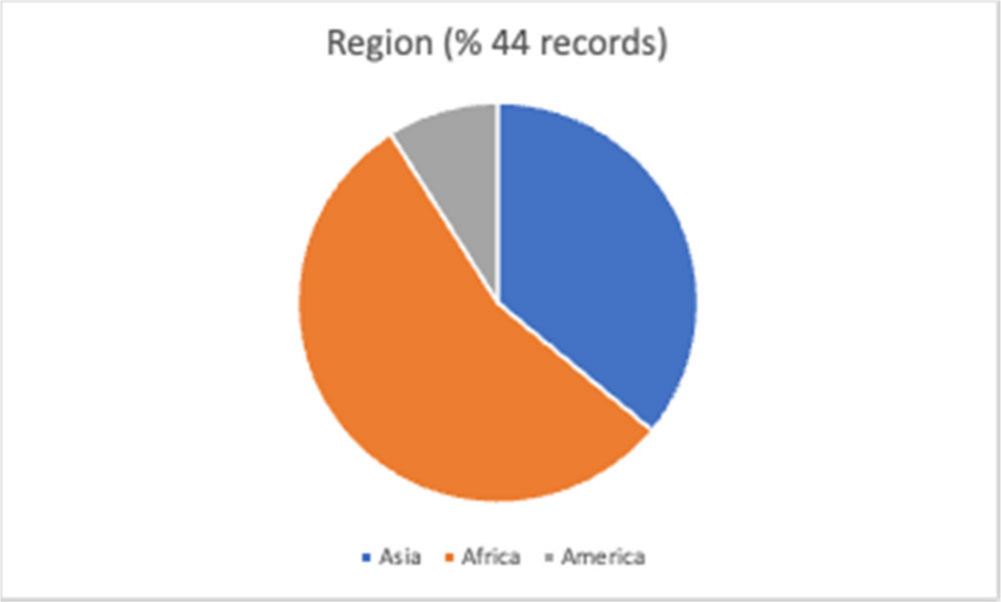
Region (male engagement studies)

**Figure 12:**
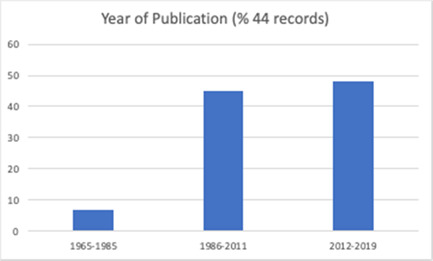
Year of publication (male engagement studies)

**Figure 13:**
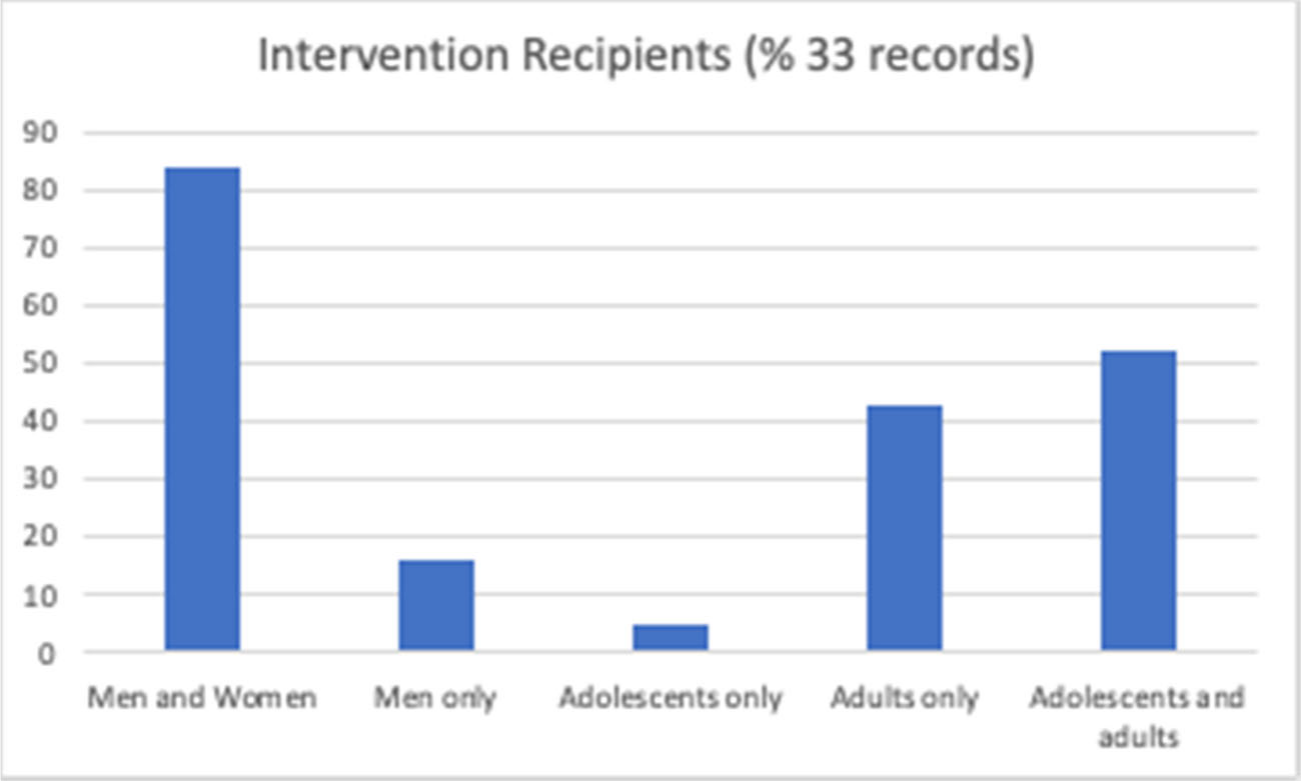
Intervention recipients (male engagement studies)

**Figure 14:**
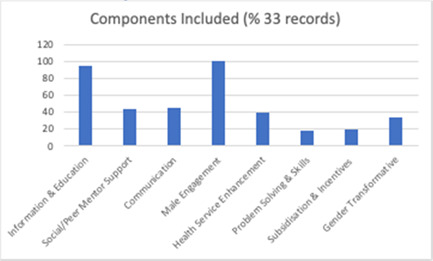
Intervention recipients (male engagement studies)

**Figure 15:**
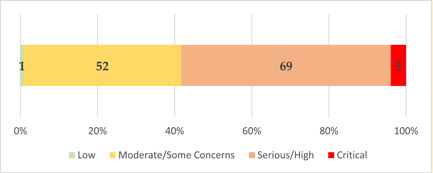
Overall risk of bias for evaluation studies

The number of components judged present in each intervention, based on the descriptions provided by study authors, ranged from 1 to 6 (Mean = 3.8). Most studies (*n* = 116, 91%) described an intervention comprised of multiple different component types, with 51.9% of described interventions comprising two or three different component types. Noted within evaluations of these complex interventions was the difficulty in parsing the effects of different components and strategies (see Firestone et al., 2016). Descriptive information for included studies is summarised graphically in Figures 9, 10, 11, 12, 13, 14, 15.

**Figure 16:**
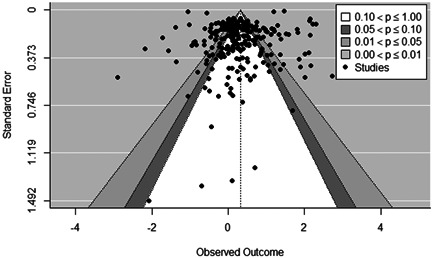
Funnel plot examining publication bias

**Figure 17:**
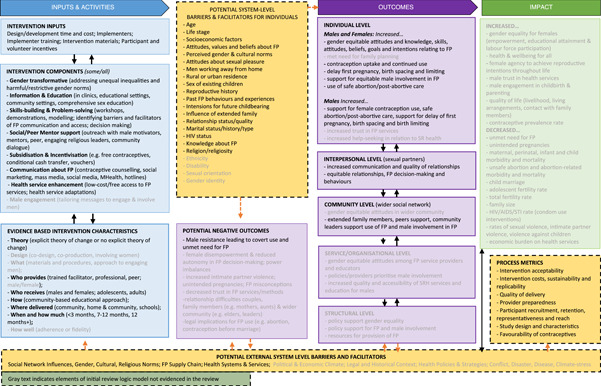
Revised review logic model

####### Reported outcomes

Few studies distinguished whether effects were related to the study's primary or secondary outcomes. Outcomes were judged to be measured at various levels: individual (e.g., relating to individual FP attitudes and behaviour beliefs), interpersonal (e.g., relating to relationship and FP behaviours between individuals), organisational (e.g., organisational policy and practice changes that increase involvement of men in FP), and structural (e.g., public policy and support for male involvement in FP).

Most outcomes assessed by the included studies were *Individual Level Outcomes* (*n* = 127) (see review logic model Supporting Information: Appendix 1.0 for examples), with some studies addressing *Interpersonal Outcomes* (*n* = 38), and few evaluating *Organisational* (*n* = 8) or *Structural Outcomes/Impacts* (*n* = 1). A minority of studies examined outcomes at multiple levels, with the most common involving a combination of individual and interpersonal level outcomes. Studies which examined outcomes at higher levels did so simultaneously with the preceding levels of outcomes. All studies that assessed Interpersonal outcomes also assessed Individual level outcomes (*n* = 38), five studies that assessed Organisation outcomes also assessed Interpersonal outcomes, and the one example assessing structural outcomes assessed outcomes at all preceding levels.

Most commonly studies targeted and assessed outcomes at the individual level (*n* = 127). By far the most common individual‐level outcomes targeted and measured were changes in contraceptive use (*n* = 72), contraceptive knowledge (*n* = 52), and changes in attitudes about FP/contraceptives (*n* = 49). Birth spacing and delay (e.g., delayed first pregnancy, intentions to limit family size, total fertility rate) was assessed chiefly in interventions delivered to adults or groups inclusive of all individuals of reproductive age (*n* = 36).

Interpersonal level outcomes were assessed in *n* = 38 studies. Of these, the most common were *Communication* (*n* = 29) and *Joint decision‐making around FP* (*n* = 12). Perhaps unsurprisingly, interventions attempting to address these were those involving males and females in delivery (*n* = 10 out of 12). The remaining two studies/interventions were delivered to males exclusively, however, these emphasised building communication skills and the promotion of joint FP decision‐making.

Organisation‐level outcomes were assessed in *n* = 8 studies and chiefly addressed increasing service engagement and accessibility for all, not necessarily specifically for males. A small number of studies, however, did consider enhancing gender equitable beliefs among service providers (*n* = 3) (Khatun et al., 2011; Timol et al., 2016; Vernon & Dura, 2004).

One study (Singh et al., 2016) that included FP as part of community health intervention delivery assessed structural level outcomes, but only assessed service use indicators and did not assess any individual level FP outcomes.

###### Study characteristics male engagement studies (n = 44)

As noted, 44 of the included studies included a male engagement component designed to actively engage men and boys in FP. The characteristics of these studies are outlined in Table 4.

Most of these 44 studies were conducted in Africa (55%), followed by Asia (36%) and America (9%), with just under half (48%) published since 2012. Approximately half (52%) of the studies were RCTs and the other 48% were quasi experimental studies. Seventy‐seven percent of the 44 studies (*n* = 34) reported contraceptive use outcomes, with 48% (*n* = 21) reporting a heterogeneous mix of outcomes relating to pregnancy, pregnancy timing and desired family size. Forty‐three percent (*n* = 19) reported attitudes about contraceptives, 36% (*n* = 16) knowledge about contraceptives, 25% (*n* = 11) communication about FP, 25% (*n* = 11) service use and 18% (*n* = 8) joint FP decision‐making.

Most of the interventions in the included studies had a community‐based educational (59%, *n* = 26) or contraceptive counselling (34%, *n* = 15) focus with a small number (7%, *n* = 3) focussed on maternal and child health. Eighty‐seven percent (*n* = 38) of the studies included interventions that contained between 3 and 6 components, with all but two (95%) including an information and education element. Forty‐five percent (*n* = 20) included a communication component, 43% (*n* = 19) a social/peer mentor component, 39% (*n* = 17) a health service enhancement component and 34% (*n* = 15) a gender transformative component.

The majority of interventions (84%, *n* = 37) were delivered to men and women. This subset was slightly different than all included studies in that it included fewer interventions that targeted adolescents only (only 5% (*n* = 2) of male engagement studies targeted adolescents only compared with 31% (*n* = 39) of all included studies). Of male engagement studies more targeted adult populations (43% (*n* = 19) compared with 24% (*n* = 31) of all included studies. The studies showed an almost even range of dosage timeframes (ranging from 18% (*n* = 8) for 7 to 12 months to 15% (*n* = 11) for >12 months). Half of the interventions were delivered using mixed delivery modes (50%, *n* = 22) with the remainder delivered to groups only (25%, *n* = 11) or individuals only (20%, *n* = 9), couples only (2%, *n* = 1) or not specified (2%, *n* = 1). In relation to intervention providers, professionals were the most common (39%, *n* = 17), followed by trained facilitators (27%, *n* = 12) and mixed providers (16%, *n* = 7). Four studies (9%) used peers only, and 3 (7%) used MHealth only.

###### Study characteristics connected papers (n = 23)

An overview of the characteristics of these 23 included studies can be found in Supporting Information: Appendix 4.2. Almost three‐quarters of the studies (*N* = 17, 74%) were published since 2012, with 11 (48%) published between 2015 and 2019. Two studies (Ross et al., 2007; Bertrand et al., 1982) reported findings that were more than 40 years old. Fifteen of the studies were process evaluations and eight were qualitative studies, all directly connected to the evaluations outlined above.

Four of the papers (Harrington et al., 2016; Harrington 2017a, 2017b; Harrington et al., 2019) related to the same MHealth intervention (MobileWatchXY) and three others (McCarthy et al., 2018a, 2018b, 2019) to TFPA's ‘Healthy Lifestyles’ app. Nine of the study samples were pregnant or postpartum women and their partners and six included adolescents. Most of the process evaluations aimed to explore the feasibility of ongoing or future experimental work and reported development and acceptability of planned interventions. Qualitative studies explored perceptions of FP, couple communication about FP, gendered power dynamics and women's negotiation of FP, and barriers and facilitators of FP uptake or to including men and boys in FP.

### 5.2 Risk of bias in included studies

A full breakdown of risk of bias judgements for all studies may be found in Supporting Information: Appendix 5.0.

#### 5.2.1 Evaluation studies

The risk of bias across included studies is summarised in Figure 15. Overall, only one study was judged to have a Low risk of bias. Most were found to have a Moderate to High risk of bias with five determined to have an irreconcilable or ‘*Critical*’ risk of bias. A full breakdown of risk of bias judgements for all evaluation studies can be found in Supporting Information: Appendices 5.1. and 5.2.

Those quasi‐experimental records identified to have a critical risk of bias (*n* = 5) predominately received this classification due to under‐reporting of study design and methodology. The majority of quasi‐experimental studies were judged to have a *Serious* risk of bias (70.6%). Most records were judged to have a Serious risk of bias in multiple domains, however most common *Serious* judgements related to the treatment of missing data (ROBINS‐I: Domain 5) and potential bias due to confounding (ROBINS‐I: Domain 1).

In total *n* = 21 RCT studies were judged to have a High risk of bias, with records showing a high risk of bias across multiple domains. However, the most common judgements were for deviations from intended intervention (RoB2.0: Domain 2b) and risk of bias arising from randomisation process (RoB2.0: Domain 1).

#### 5.2.2 Connected papers

We appraised qualitative studies using Jimenez et al. (2018) critical appraisal tool and quantitative process evaluation studies using the EPPI Centre Tool (EPPI‐Centre & EPPI‐Centre Social Science Research Unit, 2003) (see Table 6). A full breakdown of risk of bias judgements for all domains of each tool is included in Supporting Information: Appendix 5.0. Three process evaluations (Daniele, 2017; Mantell et al., 2006) were judged to have low risk of bias, while the remainder were judged to have moderate risk of bias. One qualitative study (McCarthy, 2019) was judged to have a high risk of bias, because of lack of full reporting on several of the domains.

**Table 6:** Impact of male engagement FP interventions on intermediate outcomes

Outcome	*N* (*k*)	OR	CI	PI	*p* Value
Attitudes FP Services	3 (9)	1.16	0.75–1.77	0.34–3.94	0.18
Contraceptive Attitudes	8 (25)	1.26	0.97–1.64	0.39–4.10	0.02
Contraceptive Knowledge	10 (33)	1.19	0.93–1.52	0.37–3.86	0.40
FP Communication	5 (9)	1.20	0.78–1.84	0.35–4.09	0.28
Gender Equitable Behaviours & Beliefs	3 (7)	2.55	1.56–4.17	0.73–8.90	0.20
Equitable FP Decision‐making	3 (8)	0.95	0.60–1.52	0.28–3.30	0.89
FP Service Use	5 (25)	1.36	0.94–1.96	0.41–4.54	0.14

*Note*: Bold text indicates adequately powered analysis, the other variables had too few studies reporting the outcome of interest to be adequately powered.

Abbreviations: CI, confidence interval; *k*, number of effect size estimates; *N*, number of studies; OR, odds ratio; PI, prediction interval.

### 5.3 Synthesis of results—Causal chain analysis

#### 5.3.1 Review question 2: What are the impacts of FP interventions involving men and boys on FP outcomes?

##### The effects of FP interventions on ‘contraceptive use’ outcomes

The meta‐analysis of 72 studies (*k* = 265) revealed that the FP interventions had statistically significantly higher odds of improving contraceptive use when compared to comparison groups (OR = 1.38, CI = 1.21 to 1.57, PI = 0.36 to 5.31, *p* < 0.0001). The groups who received the FP interventions were one and a third times more likely to experience improved contraceptive use.

As there were substantial variations between the studies in terms of their effect sizes (heterogeneity *Q* = 40,647, df = 264, *p* < 0.0001; *I* ^2^ = 98%), we investigated *I* ^2^ further and found that 25% of heterogeneity was between cluster/study and 73% was within cluster/study. We know that the multilevel model contains two variance components (sigma^2_1 and sigma^2_2), for the between‐cluster heterogeneity and the within‐cluster heterogeneity. Therefore, about 25% of the total variance is estimated to be due to between‐cluster heterogeneity, 70% due to within‐cluster heterogeneity, and the remaining 5% are sampling variance. This is an investigation of the total remaining variance after outliers were removed following the process outlined by (Viechtbauer, 2010).

To test for publication bias, a weighted regression with multiplicative dispersion using sampling variance as a predictor was utilised. This test found no evidence of publication bias (*p* = 0.48) (see Figure 16), indicating that there was an accurate representation of the literature of interest.

##### The effects of male engagement interventions on contraceptive use and intermediate FP outcomes

When we separated the male engagement studies that reported contraceptive use from the larger dataset, we examined the impact of these interventions (male engagement interventions with a contraceptive use outcome (*n* = 33 *k* = 226) on intermediate outcomes measured by the included studies. Identified intermediate outcomes included: attitudes about FP services; attitudes about contraception; knowledge about contraception; FP communication; gender equitable beliefs; joint FP decision‐making; and FP service use. We conducted multivariate pairwise meta‐analysis across 33 studies (*k* = 226) but urge caution in interpretation as Tipton demonstrates that the Satterthwaite approximation is valid so long as the df is >4 and this is only the case for contraceptive attitudes and contraceptive knowledge in these exploratory analyses, indicating that the analysis is underpowered for outcomes. To allow for estimation of the variance components the Satterthwaite approximation was used to account for two different sample variances where only estimates of the variance are known. The analysis is useful to calculate an approximation to the effective degrees of freedom (Keenan et al., 2021).

While the male engagement interventions appear to be improving the outcomes measured (the ORs were >1 for all outcomes apart from equitable FP decision‐making (OR = 0.95)), the results were statistically significant only for contraceptive attitudes (OR = 1.26, CI 0.97–1.64, *p* = .02). Table 7 presents individual findings for each of the outcomes.

**Table 7:** Summary of correlated effects meta‐regression results linking intervention components to contraceptive use

	*n* (*k*)	OR	*t* (df)	*p* Value	95% CI	95% PI
*Component*	72 (265)					
Gender Transformative		1.04	0.293 (22.29)	0.772	0.73–1.50	0.25–4.34
Information & Education		1.30	0.836 (8.09)	0.427	0.76–2.23	0.30–5.71
Male Engagement		0.95	−0.412 (41.99)	0.682	0.71–1.26	0.23–3.87
Problem Solving and Skills		1.10	0.348 (5.94)	0.740	0.68–1.78	0.25–4.73
Social/Peer Support		0.86	−1.105 (41.81)	0.275	0.64–1.16	0.21–3.52
Subsidised/Incentivised Contraception		1.24	1.329 (36.55)	0.192	0.93–1.65	0.30–5.05
Communication		0.82	−1.524 (39.14)	0.136	0.61–1.10	0.20–3.35
Health Service Enhancement		1.16	1.032 (40.04)	0.308	0.87–1.54	0.28–4.74

Abbreviations: 95% CI, 95% confidence intervals for the meta‐regression coefficients; *k*, effect estimates; *n*, number of studies; OR, odds ratio; PI, 95% prediction intervals for the meta‐regression coefficients.

#### 5.3.2 Review question 3: What are the effective components of interventions that achieve positive change in contraceptive use outcomes?

Table 8 summarises results from the meta‐regressions across 72 studies (*k* = 265) with all 8 identified intervention components added in the model. The test of moderators provides an omnibus test of all components (QM(df = 8) = 27.5844, *p* = 0.0006) and this indicates that the explained variance across this data is significantly greater than the unexplained variance, overall.

**Table 8:** Summary of correlated effects meta‐regression results linking combinations of intervention components to contraceptive use

Combination	*n* (*k*)	OR	*t* (df)	*p* Value	CI	PI
G + I + PSS	1 (2)	1.78	13.2 (1)	0.05	0.50–6.32	0.27–11.91
G + I + PSS + C	3 (8)	1.42	1.08 (1.98)	0.40	0.69–2.93	0.29–6.96
G + I + PSS + C + H	2 (3)	1.52	9.22 (1)	0.07	0.59–3.89	0.28–8.32
G + I + PSS + IN	1 (2)	0.67	−9110 (1)	0.00	0.20–2.28	0.10–4.36
G + I + PSS + SO	4 (8)	1.41	4.25 (2.87)	0.03	0.75–2.66	0.30–6.66
G + I + PSS + SO + C	2 (6)	1.00	0.262 (1)	0.84	0.44–2.28	0.19–5.16
G + I + PSS + SO + C + H	1 (2)	1.79	6940 (1)	0.00	0.43–7.37	0.24–13.26
G + I + PSS + SO + IN + C + H	2 (8)	1.10	0.716 (1)	0.60	0.48–2.55	0.21–5.73
I + PSS	10 (49)	1.08	0.383 (8.06)	0.71	0.74–1.57	0.25–4.67
I + PSS + C	3 (16)	1.14	0.457 (1.71)	0.70	0.57–2.29	0.24–5.53
I + PSS + C + H	1 (1)	3.66	NA	NA	0.87–15.42	0.49–27.57
I + PSS + H	4 (13)	1.30	1.65 (2.79)	0.20	0.72–2.33	0.28–6.01
I + PSS + IN	5 (24)	2.90	2.55 (3.74)	0.07	1.72–4.88	0.64–13.11
I + PSS + IN + C	1 (4)	2.23	322 (1)	0.00	0.76–6.58	0.37–13.27
I + PSS + IN + C + H	2 (6)	1.28	4.32 (1)	0.14	0.55–2.97	0.25–6.64
I + PSS + IN + H	2 (12)	2.35	0.745 (1)	0.59	1.08–5.12	0.47–11.83
I + PSS + SO	2 (4)	1.04	0.118 (1)	0.93	0.43–2.53	0.20–5.55
I + PSS + SO + C	3 (3)	0.97	−0.127 (1.97)	0.91	0.41–2.31	0.18–5.11
I + PSS + SO + C + H	4 (23)	1.19	0.936 (2.84)	0.42	0.68–2.09	0.26–5.48
I + PSS + SO + H	1 (8)	1.10	4.81 (1)	0.13	0.39–3.05	0.19–6.29
I + PSS + SO + IN	2 (8)	1.51	1.6 (1)	0.36	0.69–3.33	0.30–7.66
I + PSS + SO + IN + C	4 (13)	1.03	0.111 (2.68)	0.92	0.56–1.88	0.22–4.80
I + PSS + SO + IN + C + H	2 (10)	0.98	−0.0466 (1)	0.97	0.46–2.11	0.20–4.92
I + PSS + SO + IN + H	4 (10)	2.19	2.39 (2.63)	0.11	1.16–4.16	0.46–10.38
I + SO + C	1 (6)	1.46	9.52 (1)	0.07	0.52–4.12	0.25–8.45
I + SO + H	1 (5)	2.20	133 (1)	0.00	0.76–6.35	0.38–12.92
I + SO + IN + C + H	1 (4)	0.88	−1180 (1)	0.00	0.30–2.58	0.15–5.20
PSS + H	1 (1)	3.07	NA	NA	0.64–14.80	0.37–25.5
PSS + IN	1 (5)	1.16	8.85 (1)	0.07	0.41–3.31	0.2–6.77

*Note*: Bold text indicates adequately powered analysis, the other variables had too few studies reporting the outcome of interest to be adequately powered.

Abbreviations: C, Communication; G, Gender Transformative; H, Health Service Enhancement; I, Information & Education; IN, subsidised or incentivised contraception; ME, Male Engagement; PSS, Problem Solving & Skills; SO, Social/Peer/Mentor Support.

As highlighted in Table 8, none of the components were individually more effective than the others in improving contraceptive use. ‘Information and Education’ (OR = 1.30, 95% CI 0.76–2.23, *p* = 0.34), ‘subsidised or incentivised contraception’ (OR = 1.24, 95% CI 0.93–1.65, *p* = 0.15), ‘health service enhancement’ (OR = 1.16, 95% CI 0.87–1.54, *p* = 0.31), problem‐solving and skills (OR = 1.1, 95% CI 0.68–1.78, *p* = 0.71) and ‘gender transformative’ (OR = 1.04, 95% CI 0.73–1.5, *p* = 0.82) components had non‐significant positive effects. The remaining three components, ‘communication’, ‘social/peer support’, and ‘male engagement’ had non‐significant negative effects.

We undertook further analysis to assess the combination of components. However, given that there were 33 identified combinations of components in the included studies this exploratory analysis should be interpreted with caution. As illustrated in Table 9, the only combination of components adequately powered to detect moderating effects were interventions that included ‘information & education’ and ‘problem solving & skills’ components. Interventions that used this combination of components did not show statistically significant effects (OR = 1.08, 95% CI 0.74–1.57, *p* = 0.71).

**Table 9:** Summary of correlated effects meta‐regression results on contraceptive use and extrinsic, methodological, and substantive variables

Variables	Categories	*n* (*k*)	estimate (SE)	*t* (df Satt)	CI	*p* Value (Satt)	Test of moderators
**Extrinsic variable**							
Year	1960–1969	1 (1)	0.73 (0.693)	NA	−0.628–2.089	NA	*F*(df1 = 6, df2 = 259) = 36.17, *p* ≤ 0.0001
	1970–1979	2 (13)	0.34 (0.327)	1.166 (1)	−0.301–0.98	0.451	
	1980–1987	1 (1)	2.157 (0.805)	NA	0.58–3.735	NA	
	1980–1989	3 (17)	0.128 (0.262)	0.282 (1.99)	−0.387–0.642	0.804	
	1990–1999	7 (20)	0.58 (0.204)	1.925 (5.49)	0.179–0.98	0.107	
	2000–2009	27 (100)	0.403 (0.102)	4.002 (23.57)	0.203–0.603	<0.001	
	2010–2019	31 (113)	0.17 (0.1)	2.24 (25.27)	−0.025–0.365	0.034	
**Methodological variables**							
Study design	c‐RCT	4 (15)	0.114 (0.267)	0.362 (2.8)	−0.409–0.638	0.743	*F*(df1 = 3, df2 = 262) = 25.42, *p* < 0.0001
	RCT	27 (109)	0.383 (0.088)	3.905 (35.8)	0.211–0.555	<0.001	
	QE	41 (141)	0.266 (0.107)	3.306 (22.7)	0.055–0.476	0.003	
**Substantive variables**							
Behaviour change theory	TOC present	20 (67)	0.388 (0.128)	2.86 (16.9)	0.137–0.639	0.011	*F*(df1 = 2, df2 = 263) = 24.86, *p* < 0.001
	TOC not present	52 (198)	0.299 (0.075)	4.05 (45.2)	0.151–0.447	<0.001	
Intervention Provider	Media	2 (7)	0.184 (0.347)	0.328 (1)	−0.496–0.864	0.798	*F*(df1 = 10, df2 = 255) = 47.78, *p* ≤ 0.001
	Mhealth	4 (17)	0.341 (0.271)	0.836 (2.24)	−0.189–0.871	0.483	
	Peers	9 (22)	0.219 (0.177)	2.72 (6.89)	−0.128–0.566	0.030	
	Peer & Media	1 (6)	0.001 (0.407)	4.25E + 15 (1)	−0.796–0.798	<0.001	
	Professionals	21 (102)	0.284 (0.103)	2.82 (17.53)	0.082–0.485	0.011	
	Professionals & Media	2 (8)	−0.208 (0.333)	−1.19 (1)	−0.861–0.445	0.444	
	Professionals & Peers	13 (42)	0.2 (0.144)	1.79 (10.08)	−0.082–0.482	0.103	
	Trained Facilitator	12 (32)	0.632 (0.155)	3.35 (9.76)	0.328–0.935	0.008	
	Trained Facilitator & Peers	4 (23)	0.128 (0.231)	0.582 (2.57)	−0.325–0.581	0.608	
	Not specified	4 (6)	1.379 (0.329)	2.68 (2.63)	0.734–2.025	0.086	
**Dosage**	Less than 3 months	16 (61)	0.378 (0.143)	2.648 (13.52)	0.098–0.657	0.02	*F*(df1 = 6, df2 = 259) = 26.15 *p* = .0002
	3–6 months	13 (39)	0.217 (0.174)	1.475 (10.37)	−0.124–0.557	0.17	
	7–12 months	13 (50)	0.48 (0.159)	4.382 (10.65)	0.169–0.792	0.001	
	12+ months	24 (104)	0.3 (0.109)	2.358 (21.68)	0.086–0.513	0.028	
	Mixed	5 (10)	0.175 (0.286)	0.747 (3.81)	−0.386–0.736	0.498	
	Not specified	1 (1)	−0.582 (0.816)	NA	−2.182–1.017	NA	
**Sex**	Male only	8 (17)	0.336 (0.232)	1.46 (5.77)	−0.119–0.79	0.196	*F*(df1 = 2, df2 = 263) = 24.31, *p* < 0.0002
	Male and Female	64 (248)	0.321 (0.068)	4.72 (56.78)	0.188–0.454	<0.001	
**Age**	Adolescents only	14 (51)	0.392 (0.153)	2.97 (11.5)	0.093–0.691	0.012	*F*(df1 = 3, df2 = 262) = 24.36, *p* < 0.0001
	Adults only	20 (89)	0.346 (0.125)	2.67 (16.5)	0.101–0.591	0.017	
	Both age groups	38 (125)	0.287 (0.09)	3.13 (33.3)	0.11–0.463	0.004	
**Setting**	Community	25 (82)	0.322 (0.113)	2.724 (21.41)	0.101–0.544	0.013	*F*(df1 = 9, df2 = 256) = 29.4395, *p* = 0.0005
	Healthcare	17 (72)	0.283 (0.131)	1.876 (14.18)	0.025–0.54	0.081	
	Home only	3 (6)	0.33 (0.373)	0.9 (1.93)	−0.401–1.06	0.466	
	Home & Community	8 (36)	0.388 (0.193)	3.46 (6.01)	0.01–0.765	0.013	
	Home & Healthcare	1 (4)	0.802 (0.493)	463.944 (1)	−0.164–1.768	0.001	
	Schools	11 (42)	0.372 (0.167)	3 (9.1)	0.045–0.7	0.015	
	Schools & Healthcare	4 (14)	0.484 (0.275)	1.955 (2.59)	−0.055–1.023	0.160	
	Community & Schools	1 (1)	−0.582 (0.81)	NA	−2.17–1.005	NA	
	Not specified	2 (8)	−0.292 (0.387)	−0.456 (1)	−1.05–0.466	0.727	
**Region**	Asia	2 (5)	1.226 (0.426)	5.565 (1)	0.392–2.06	0.112	*F*(df1 = 16, df2 = 249) = 45.3192, *p* = 0.0001
	Caribbean (Americas)	1 (2)	0.407 (0.572)	53.259 (1)	−0.714–1.528	0.012	
	Central Africa	1 (4)	0.197 (0.476)	1024.592 (1)	−0.735–1.129	<0.001	
	Central America	2 (8)	0.304 (0.35)	3.188 (1)	−0.382–0.989	0.194	
	Central Asia	1 (2)	−0.767 (0.713)	−57.865 (1)	−2.164–0.629	0.011	
	East Africa	19 (79)	0.226 (0.118)	3.163 (16.23)	−0.006–0.457	0.006	
	East Africa, Southern Africa	1 (4)	−0.021 (0.468)	−99.528 (1)	−0.939–0.897	0.006	
	East Asia	6 (17)	0.488 (0.222)	1.178 (4.35)	0.053–0.922	0.299	
	Middle East (Africa)	3 (21)	0.137 (0.27)	1.604 (1.81)	−0.392–0.666	0.263	
	North America	4 (21)	0.568 (0.27)	2.071 (2.27)	0.039–1.096	0.159	
	South America	3 (11)	0.43 (0.289)	0.929 (1.75)	−0.137–0.997	0.463	
	South America, Central America	2 (3)	−0.08 (0.517)	−0.183 (1)	−1.093–0.935	0.885	
	South America, South Asia	1 (7)	−0.704 (0.439)	−7.177 (1)	−1.563–0.156	0.088	
	South Asia	9 (31)	0.558 (0.182)	2.154 (6.94)	0.201–0.915	0.069	
	Southern Africa	6 (21)	0.112 (0.216)	1.065 (4.49)	−0.311–0.535	0.341	
	West Africa	11 (29)	0.458 (0.176)	3.844 (8.69)	0.114–0.801	0.004	
**Intervention design**	Community Based Educational	56 (193)	0.349 (0.072)	4.692 (48.27)	0.208–0.491	<0.001	*F*(df1 = 3, df2 = 106) = 31.2429, *p* < 0.0001
	Contraceptive Counselling	12 (50)	0.066 (0.148)	0.467 (9.6)	−0.223–0.356	0.651	
	Maternal & Child Health Programme	4 (22)	0.705 (0.254)	5.708 (2.42)	0.207–1.202	0.019	

*Note*: Effect sizes in bold are statistically significantly different from zero at alpha level *α* = 0.05 with df > 4. The results also indicated that some variables relating to **study context and design characteristics** were predictors of contraceptive use. First, studies that took place in the *regions* of West Africa (estimate = 0.46, *p* = 0.004) and East Africa (estimate = 0.23, *p* = 0.006), showed a statistically significant moderating effect. Further, y*ear of publication* emerged as important, with more recent studies published between 2000 and 2009 (estimate = 0.40, *p* < 0.001) and 2010–2019 (estimate = 0.17, *p* = 0.04) showing statistically significant effects. Finally, *study design* was highlighted as a moderator, with RCTs (estimate = 0.38, *p* < 0.001) and quasi‐experiments (estimate = 0.27, *p* = 0.003) showing statistically significant moderating effects. Cluster RCTs did not show statistically significant effects (*p* = 0.74).

Abbreviations: CI, 95% confidence interval; *k*, number of effect estimates; *n*, number of studies.

#### 5.3.3 Review questions 4 and 5: What characteristics and combinations of characteristics are associated with positive FP‐related outcomes? Do outcomes vary by context and participant characteristics?

##### All included studies (*n* = 127)

In Table 10, we present ten potential moderators of contraceptive use using robust variance estimates. This exploratory analysis used a single‐variable, no‐intercept model. Estimates presented are ORs.

**Table 10:** Summary of correlated effects meta‐regression results on extrinsic, methodological, and substantive variables

Variables	Categories	*n* (*k*)	estimate (SE)	*t* (df Satt)	CI	*p* Value (Satt)	Test of moderators
**Extrinsic variables**							
Year	1960–1969	1 (1)	0.73 (0.693)	NA	−0.628–2.089	NA	*F*(df1 = 6, df2 = 259) = 36.17, *p* ≤ 0.0001
	1970–1979	2 (13)	0.34 (0.327)	1.166 (1)	−0.301–0.98	0.451	
	1980–1987	1 (1)	2.157 (0.805)	NA	0.58–3.735	NA	
	1980–1989	3 (17)	0.128 (0.262)	0.282 (1.99)	−0.387–0.642	0.804	
	1990–1999	7 (20)	0.58 (0.204)	1.925 (5.49)	0.179–0.98	0.107	
	**2000**–**2009**	**27 (100)**	**0.403 (0.102)**	**4.002 (23.57)**	**0.203–0.603**	**<0.001**	
	**2010**–**2019**	**31 (113)**	**0.17 (0.1)**	**2.24 (25.27)**	**−0.025–0.365**	**0.034**	
**Methodological variables**							
Study design	c‐RCT	4 (15)	0.114 (0.267)	0.362 (2.8)	−0.409–0.638	0.743	*F*(df1 = 3, df2 = 262) = 25.42, *p* < 0.0001
	**RCT**	**27 (109)**	**0.383 (0.088)**	**3.905 (35.8)**	**0.211–0.555**	**<0.001**	
	**QE**	**41 (141)**	**0.266 (0.107)**	**3.306 (22.7)**	**0.055–0.476**	**0.003**	
**Substantive variables**							
Behaviour change theory	**TOC present**	**20 (67)**	**0.388 (0.128)**	**2.86 (16.9)**	**0.137–0.639**	**0.011**	*F*(df1 = 2, df2 = 263) = 24.86, *p* < 0.001
	**TOC not present**	**52 (198)**	**0.299 (0.075)**	**4.05 (45.2)**	**0.151–0.447**	**<0.001**	
Intervention Provider	Media	2 (7)	0.184 (0.347)	0.328 (1)	−0.496–0.864	0.798	*F*(df1 = 10, df2 = 255) = 47.78, *p* ≤ 0.001
	Mhealth	4 (17)	0.341 (0.271)	0.836 (2.24)	−0.189–0.871	0.483	
	**Peers**	**9 (22)**	**0.219 (0.177)**	**2.72 (6.89)**	**−0.128–0.566**	**0.030**	
	Peer & Media	1 (6)	0.001 (0.407)	4.25E + 15 (1)	−0.796–0.798	<0.001	
	**Professionals**	**21 (102)**	**0.284 (0.103)**	**2.82 (17.53)**	**0.082**–**0.485**	**0.011**	
	Professionals & Media	2 (8)	−0.208 (0.333)	−1.19 (1)	−0.861–0.445	0.444	
	Professionals & Peers	13 (42)	0.2 (0.144)	1.79 (10.08)	−0.082–0.482	0.103	
	**Trained Facilitator**	**12 (32)**	**0.632 (0.155)**	**3.35 (9.76)**	**0.328–0.935**	**0.008**	
	Trained Facilitator & Peers	4 (23)	0.128 (0.231)	0.582 (2.57)	−0.325–0.581	0.608	
	Not specified	4 (6)	1.379 (0.329)	2.68 (2.63)	0.734–2.025	0.086	
Dosage	**Less than 3 months**	**16 (61)**	**0.378 (0.143)**	**2.648 (13.52)**	**0.098–0.657**	**0.02**	*F*(df1 = 6, df2 = 259) = 26.15 *p* = 0.0002
	3–6 months	13 (39)	0.217 (0.174)	1.475 (10.37)	−0.124–0.557	0.17	
	**7**–**12 months**	**13 (50)**	**0.48 (0.159)**	**4.382 (10.65)**	**0.169–0.792**	**0.001**	
	**12**+ **months**	**24 (104)**	**0.3 (0.109)**	**2.358 (21.68)**	**0.086–0.513**	**0.028**	
	Mixed	5 (10)	0.175 (0.286)	0.747 (3.81)	−0.386–0.736	0.498	
	Not specified	1 (1)	−0.582 (0.816)	NA	−2.182–1.017	NA	
Sex	Male only	8 (17)	0.336 (0.232)	1.46 (5.77)	−0.119–0.79	0.196	*F*(df1 = 2, df2 = 263) = 24.31, *p* < 0.0002
	**Male and Female**	**64 (248)**	**0.321 (0.068)**	**4.72 (56.78)**	**0.188–0.454**	**<0.001**	
Age	**Adolescents only**	**14 (51)**	**0.392 (0.153)**	**2.97 (11.5)**	**0.093–0.691**	**0.012**	*F*(df1 = 3, df2 = 262) = 24.36, *p* < 0.0001
	**Adults only**	**20 (89)**	**0.346 (0.125)**	**2.67 (16.5)**	**0.101–0.591**	**0.017**	
	**Both age groups**	**38 (125)**	**0.287 (0.09)**	**3.13 (33.3)**	**0.11–0.463**	**0.004**	
Setting	**Community**	**25 (82)**	**0.322 (0.113)**	**2.724 (21.41)**	**0.101–0.544**	**0.013**	*F*(df1 = 9, df2 = 256) = 29.4395, *p* = 0.0005
	Healthcare	17 (72)	0.283 (0.131)	1.876 (14.18)	0.025–0.54	0.081	
	Home only	3 (6)	0.33 (0.373)	0.9 (1.93)	−0.401–1.06	0.466	
	**Home & Community**	**8 (36)**	**0.388 (0.193)**	**3.46 (6.01)**	**0.01–0.765**	**0.013**	
	Home & Healthcare	1 (4)	0.802 (0.493)	463.944 (1)	−0.164–1.768	0.001	
	**Schools**	**11 (42)**	**0.372 (0.167)**	**3 (9.1)**	**0.045–0.7**	**0.015**	
	Schools & Healthcare	4 (14)	0.484 (0.275)	1.955 (2.59)	−0.055–1.023	0.160	
	Community & Schools	1 (1)	−0.582 (0.81)	NA	−2.17–1.005	NA	
	Not specified	2 (8)	−0.292 (0.387)	−0.456 (1)	−1.05–0.466	0.727	
Region	Asia	2 (5)	1.226 (0.426)	5.565 (1)	0.392–2.06	0.112	*F*(df1 = 16, df2 = 249) = 45.3192, *p* = 0.0001
	Caribbean (Americas)	1 (2)	0.407 (0.572)	53.259 (1)	−0.714–1.528	0.012	
	Central Africa	1 (4)	0.197 (0.476)	1024.592 (1)	−0.735–1.129	<0.001	
	Central America	2 (8)	0.304 (0.35)	3.188 (1)	−0.382–0.989	0.194	
	Central Asia	1 (2)	−0.767 (0.713)	−57.865 (1)	−2.164–0.629	0.011	
	**East Africa**	**19 (79)**	**0.226 (0.118)**	**3.163 (16.23)**	**−0.006–0.457**	**0.006**	
	East Africa, Southern Africa	1 (4)	−0.021 (0.468)	−99.528 (1)	−0.939–0.897	0.006	
	East Asia	6 (17)	0.488 (0.222)	1.178 (4.35)	0.053–0.922	0.299	
	Middle East (Africa)	3 (21)	0.137 (0.27)	1.604 (1.81)	−0.392–0.666	0.263	
	North America	4 (21)	0.568 (0.27)	2.071 (2.27)	0.039–1.096	0.159	
	South America	3 (11)	0.43 (0.289)	0.929 (1.75)	−0.137–0.997	0.463	
	South America, Central America	2 (3)	−0.08 (0.517)	−0.183 (1)	−1.093–0.935	0.885	
	South America, South Asia	1 (7)	−0.704 (0.439)	−7.177 (1)	−1.563–0.156	0.088	
	South Asia	9 (31)	0.558 (0.182)	2.154 (6.94)	0.201–0.915	0.069	
	Southern Africa	6 (21)	0.112 (0.216)	1.065 (4.49)	−0.311–0.535	0.341	
	**West Africa**	**11 (29)**	**0.458 (0.176)**	**3.844 (8.69)**	**0.114–0.801**	**0.004**	
**Intervention design**	**Community Based Educational**	**56 (193)**	**0.349 (0.072)**	**4.692 (48.27)**	**0.208–0.491**	**<0.001**	*F*(df1 = 3, df2 = 106) = 31.2429, *p* < 0.0001
	Contraceptive Counselling	12 (50)	0.066 (0.148)	0.467 (9.6)	−0.223–0.356	0.651	
	Maternal & Child Health Programme	4 (22)	0.705 (0.254)	5.708 (2.42)	0.207–1.202	0.019	

*Note*: Effect sizes in bold are statistically significantly different from zero at alpha level *α* = 0.05 with df > 4.

Abbreviations: CI, 95% confidence interval; *k*, number of effect estimates; *n*, number of studies.

Significant differences in effects were associated with year of publication (*F*_6,259_ = 36.17, *p* < 0.0001), study design (*F*_3,262_ = 25.42, *p* < 0.0001), whether or not there was a behaviour change theory present (*F*_2,263_ = 24.86, *p* < 0.0001), who the intervention provider was (*F*_10,255_ = 47.78, *p* < 0.001), dosage (*F*_6,259_ = 26.15, *p* = 0.0002), sex of intervention recipients (*F*_2,263_ = 24.31, *p* < 0.0002), age of intervention recipients (*F*_3,262_ = 24.36, *p* < 0.001), the setting in which the intervention was delivered (*F*_9,256_ = 29.44, *p* < 0.0005), the region in which the intervention was implemented (*F*_16,249_ = 45.32, *p* = 0.0001), and the intervention design (*F*_3,106_ = 31.24, *p* ≤ 0.0001).

These results suggest that several **intervention characteristics** predicted contraceptive use. Firstly, *intervention design* appeared to act as a moderator of effect, with community based educational interventions showing statistically significant effects (estimate = 0.35, *p* = 0.001). Interventions with designs primarily focused on contraceptive counselling (estimate = 0.07, *p* = 0.65) did not show significant differences in effect and maternal and child health programmes were not powered to detect trustworthy differences (*n* = 4, df = 2.4, *p* = 0.019). *Dosage* was also a significant moderator of effect with intervention durations of <3 months (estimate = 0.38, *p* = 0.02); 7–12 months (estimate = 0.48, *p* = 0.0001); >12 months (estimate = 0.30, *p* = 0.028) showing statistically significant effects. Interventions with a mid‐range duration of 3–6months did not show significant effects (*p* = 0.17). Both interventions based on a *theory of change* (estimate = 0.39; *p* = 0.011) and those not based on a theory of change (estimate = 0.30; *p* < 0.001) were significant moderators of effects on contraceptive use. In relation to the *setting* in which the intervention was delivered, those delivered in community only (estimate = 0.32, *p* = 0.013), home and community (estimate = 0.39, *p* = 0.013), and schools only (estimate = 0.37, *p* = 0.015) showed statistically significant moderating effects. The intervention provider or *who delivers* the intervention was also examined and revealed that interventions delivered by trained facilitators only (estimate = 0.63, *p* = 0.008), professionals only (estimate = 0.28, *p* = 0.011), and peers only (estimate = 0.22, *p* = 0.030) were significant.

The meta‐regression also highlighted that **participant characteristics** predicted contraceptive use. Across 72 studies (*k* = 265), all *age* categories of intervention recipients were statistically significant moderators: adolescents only (estimate = 0.39, *p* = 0.012); adults only (estimate = 0.35 m, *p* = 0.017); both age groups (estimate = 0.287, 0.004). Further, the *sex* of intervention recipients was a significant moderator of effects with interventions delivered to males and females (estimate = 0.32, *p* < 0.001) showing statistically significant effects. Intervention delivered to males only did not show significant effects (*p* = 0.20).

#### 5.3.4 Review question 6: What adverse impacts were reported?

None of the evaluation studies reported adverse outcomes, although one study (Harrington Elizabeth et al., 2019) did report potential negative consequences. Namely, some women were concerned male partners may suspect them of engaging in covert contraceptive use, and that factual information about potential bleeding and other side effects as a result of a LARC method may discourage male acceptance of these.

Four connected papers mentioned adverse consequences relating to involving men and boys in FP. Only two studies directly indicated evidence relating to a lack of adverse effects on family life and FP decision‐making (Daniele, 2017; Turan et al., 2001). While not directly implicated as an adverse outcome, one study (Harrington et al., 2016) discussed the possible negative implications (including the possibility of confrontation and violence) of covert contraceptive use among women. A further study (Harrington et al., 2019) also notes the potential negative consequences of male resistance to female empowerment and female contraceptive use in the context of unequal relationship power dynamics.

#### 5.3.5 Review question 7: What are the system‐ and process‐level barriers and facilitators of FP involving men and boys? A qualitative analysis

The aim of the analysis was to synthesise patterns or themes across included studies that related to author‐reported barriers and facilitators of impact. The aim was to use this data to help explain the outcome patterns identified in the quantitative analysis.

Findings of the qualitative analysis are presented below under the overarching categories of ‘system‐level’ and ‘process‐level’ barriers and facilitators of effective models of family planning involving men and boys. ‘System‐level’ refers to the characteristics of social systems in the broadest sense. These include environmental factors impacting upon where and how interventions are offered, such as economic and legal climate, as well as predominant values, norms, roles, and beliefs of individuals, families, communities, organisations, countries, and regions. ‘Process‐level’ refers to the operational aspects of research and intervention implementation processes.

System‐ and process‐ level categories were presented separately in the original review logic model (Supporting Information: Appendix 1.0) as ‘individual’, ‘external’, and ‘process’ factors, each with a number of sub‐categories, and are therefore also categorised as such below. Several new sub‐categories (themes) presented during the analysis process. These are highlighted below. Supporting Information: Appendix 6.0 provides examples of data relating to each of the themes.

##### System‐level barriers and facilitators

Findings relating to 14 individual‐level a priori categories (see below) emerged as author‐reported barriers and facilitators of effectiveness and impact in the connected papers. There was no information identified for five of the a priori categories (religion/religiosity, gender identity, sexual orientation, disability, or ethnicity). In relation to external‐level barriers and facilitators, findings related to three of the a priori categories were included in the connected papers (gender, cultural and religious norms; health systems and services; and FP supply chain). No information was retrieved relating to political and economic climate; legal and historical context; health policies and strategies; conflict; disaster; disease; or climate‐stress.

Three additional categories emerged from the thematic analysis that related to individual, interpersonal‐ and community‐level systems: (1) Knowledge about FP among individuals, couples, the wider family, and community; (2) FP communication and decision‐making norms and preferences, which emerged as a sub‐theme of the a priori ‘perceived gender and cultural norms’ category; and (3) Social network influences on decision‐making about family planning, which was closely linked with different modes of FP knowledge. Themes and sub‐themes are highlighted in bold below.

###### Barriers and facilitators affecting individuals

Analysis of the included connected papers revealed several system‐related barriers and facilitators at the individual level. Three studies mentioned **socioeconomic factors**, including educational attainment and women's employment outside the home (Bertrand et al., 1982; Mantell et al., 2014; Turan et al., 2001) as potential facilitators of FP use. Adding information on the importance of **age and life stage**, Turan et al. (2001) also reported that men who were older and more educated were more likely to engage with their FP intervention. Two studies mentioned the importance of **migrant status**, relating the negative impact of men working away from the household for periods of time as a barrier to FP uptake (Cooper et al., 2014; Daniele, 2017). One study (Bertrand et al., 1982) reported the advantage of **urban versus rural residence** when considering FP intervention implementation.

**Individual attitudes, values and beliefs about FP**, including attitudes about FP services, were indicated as important in eight studies (Cooper et al., 2014; Daniele, 2017; Doyle et al., 2014; Hartmann et al., 2012; Khan et al., 2008; Nair et al., 2019). Some reported that increased perceptions of risk caused by delayed initiation of contraception (Cooper et al., 2014) or beliefs that FP use would have economic advantages (Hartmann et al., 2012) were associated with positive impacts while misconceptions that contraception causes infertility (Khan et al., 2008) and negative attitudes about condom‐use within marriage had opposite effects (Ghule et al., 2015) Two studies noted that attitudes about reduced **sexual pleasure** acted as a barrier to condom use (Ghule et al., 2015; Khan et al., 2008). One study noted the facilitating effects of positive **past FP behaviours and experiences** indicating that a history of safe sexual practice was predictive of continued FP use (Daniele, 2017).

An additional category, relating to **knowledge about FP** was presented as relevant in seven studies (Cooper et al., 2014; M. Daniele, 2017; Harrington, 2017a; Harrington et al., 2019; Hartmann et al., 2012; Khan et al., 2008; McCarthy et al., 2018) Authors reported the beneficial impacts of increased knowledge (Cooper et al., 2014; Daniele, 2017; Harrington, 2017a; Hartmann et al., 2012) and some highlighted the damaging impacts of lack of knowledge or inaccurate knowledge on FP use (Ghule et al., 2015; Harrington, 2017a; Khan et al., 2008; O. L. McCarthy et al., 2018b). Two studies reported that knowledge played an important intermediary role in contributing to increased **couple communication** (Daniele, 2017; Hartmann et al., 2012).

Eight studies discussed the influence of **perceived gender and cultural norms** on acceptance and use of FP (M. Daniele, 2017; Ghule et al., 2015; Harrington et al., 2016; Jewkes et al., 2010; O. L. McCarthy et al., 2018b, 2019). These studies noted the inequalities that favoured men as household decision‐makers and stigmatised sex outside of marriage. **Male consent** or ‘permission’ for women's use of FP emerged as a sub‐theme of perceived gender and cultural norms (Daniele, 2017; Harrington, 2017a; Harrington et al., 2016, 2017). Some studies noted that women's acceptance of gender norms relating to FP were common (Daniele, 2017; Doyle et al., 2014; Jewkes et al., 2010) while one study highlighted women's responses to inequalities. These included ‘sweet talk’ with sexual partners or **concealed use** of contraception when they were experiencing a lack of congruence with cultural expectations on childbearing or thought joint‐decision‐making about FP unattainable (Harrington et al., 2016) One study reported an adverse impact of men ‘dominating conversations’ in couple counselling sessions (Daniele, 2017). A central barrier to couple communication about FP or promoting joint or female‐led decision‐making was perceived gender and cultural norms that saw women as responsible for family planning and cultural norms that **stigmatised men's move away from dominance** as the head of the household decision‐making (Doyle et al., 2014; Ghule et al., 2015; Harrington et al., 2016).

The importance of **communication about FP and decision‐making norms and preferences** emerged as an important theme that was not highlighted in the a priori framework. Ten studies (Daniele, 2017; K. Doyle et al., 2014; Ghule et al., 2015; Harrington, 2017a; Harrington et al., 2016; Hartmann et al., 2012; Jewkes et al., 2010; McCarthy et al., 2018b; Nair et al., 2019; Turan et al., 2001) referred to this. While some studies reported that **male decision‐making about FP** remained an accepted norm and preference for both women and men (Daniele, 2017; Ghule et al., 2015; Harrington et al., 2016, 2019), there were also reports of the positive influence of improved spousal communication and **joint decision‐making** about FP [(Daniele, 2017; Doyle et al., 2014; Harrington et al., 2017; Hartmann et al., 2012; Nair et al., 2019; Turan et al., 2001) or female led decision‐making on the contraceptive method used (Daniele, 2017). One study (Jewkes et al., 2010) reported positive impacts on cultural norms relating to **intergenerational communication** about sex. Another study reported barriers to FP relating to a lack of confidence due to prohibitive norms relating to **communicating about sex** with partners, parents, and FP service providers (O. L. McCarthy et al., 2018b).

Relatedly, three studies (M. Daniele, 2017; Harrington et al., 2019; Hartmann et al., 2012) highlighted the importance of **relationship status/quality** as a key determinant of FP use. While some noted positive impacts (Daniele, 2017), others noted the damaging impacts of unequal power dynamics in relationships (Harrington et al., 2019). Relatedly, three studies mentioned the influence **of marital status/type** on FP use (Daniele, 2017; Khan et al., 2008; McCarthy et al., 2018b). As noted, newly married couples were often subject to social expectations for early pregnancy (Khan et al., 2008; O. L. McCarthy et al., 2018b). One study (Daniele, 2017) alluded to the potential differences in men's willingness to engage with FP when they were in a monogamous versus polygamous marriage, with the latter proposed as leading to less investment in the healthcare of each wife. **HIV status** was mentioned as a key factor in two studies, with both noting that HIV positive status was associated with increased contraceptive use (Mantell et al., 2014; Ngure et al., 2012).

**Reproductive history** and intentions for future childbearing and the **sex of existing children** emerged as key influences on FP use (Ghule et al., 2015; Harrington et al., 2016; Nair et al., 2019; J. A. Ross & Bang, 1966). Two studies noted preferences for sons (Ghule et al., 2015; Nair et al., 2019) and three (Ghule et al., 2015; Harrington et al., 2016; J. A. Ross & Bang, 1966) reported the cultural significance of childbirth early in marriage. All noted that the absence of either would result in limited use of FP. Further, birth spacing norms were highlighted as important in one study (Khan et al., 2008).

While **co‐residence with extended family** (an a priori category) was not mentioned directly in any studies, mentioned in six studies was the strong influence of **perceptions of wider family expectations** on FP decision‐making (Cooper et al., 2014; M. Daniele, 2017; Ghule et al., 2015; Khan et al., 2008; McCarthy et al., 2018b; McCarthy, 2019). ‘Mothers of husbands’ were noted as particularly influential (Khan et al., 2008; O. L. McCarthy et al., 2018b). This theme appeared to be linked to the broader concept of community knowledge about FP.

###### Barriers and facilitators at the external system level

Four key themes relating to external systems emerged from the connected papers. Three of these were a priori categories (gender, cultural and religious norms; health systems and services; FP supply chain] and one additional category emerged from the thematic analysis (social network influences).

The positive influence of **social networks** beyond the family on FP uptake and use emerged as important across seven of the connected papers (Cooper et al., 2014; Daniele, 2017; Doyle et al., 2014; Ghule et al., 2015; Harrington et al., 2016; Hartmann et al., 2012; Jewkes et al., 2010). Male peers and ‘male motivators’ were seen as particularly influential facilitators.

Nine studies highlighted the broad influence of **gender, cultural and religious norms** on FP decisions (Daniele, 2017; Doyle et al., 2014; Ghule et al., 2015; Harrington et al., 2016; Jewkes et al., 2010; Khan et al., 2008; McCarthy et al., 2018b; Nair et al., 2019) Perceptions of these are noted under individual‐level factors above. Key highlighted norms included early childbearing for newly married couples (Ghule et al., 2015; Khan et al., 2008); religious beliefs norms condoning sex outside of marriage (McCarthy et al., 2018b) preferences for sons to act as heirs and provide for elderly relatives (Ghule et al., 2015; Nair et al., 2019) and males as dominant household decision‐makers (Harrington et al., 2016; Jewkes et al., 2010; Nair et al., 2019). As noted, there were suggestions that shifts in these norms resulted from engagement with the FP interventions.

**Health systems and services** was noted as an important factor by four studies (Baqui et al., 2018; Daniele, 2017; Doyle et al., 2014). Four studies discussed the impacts of incorporating FP services within existing maternal and child health (MCH) services (Baqui et al., 2018; Daniele, 2017; Doyle et al., 2014). One of these (Baqui et al., 2018) reported that the addition of FP services that engaged men and boys did not have adverse effects on existing services, while the others noted that men were concerned that they would not be welcome to attend MCH settings (Daniele, 2017) or did in fact experience barriers including overcrowded delivery rooms, as well as biased, undermining and negative attitudes from healthcare workers (Doyle et al., 2014; Nair et al., 2019).

Two studies indicated the importance of **FP supply chain**, the availability of contraceptives and services, in encouraging FP uptake and use (Ahmed et al., 2013; J. A. Ross & Bang, 1966).

###### Barriers and facilitators at the process level

Six a priori categories (intervention acceptability; intervention costs, sustainability, and replicability; quality of delivery; provider‐preparedness; participant recruitment, retention, and representativeness; and study design and characteristics) emerged as potentially important influencing factors. Two additional categories (reach and favourability of contraceptive method) also emerged as relevant.

Four studies (Akhter et al., 1993; Ghule et al., 2015; Harrington, 2017a; Harrington et al., 2019) noted the importance of **intervention acceptability**. Two studies noted the facilitating effects of culturally acceptable interventions (Harrington, 2017a; Harrington et al., 2019). Satisfaction with contraceptive methods was noted by two studies (Akhter et al., 1993; Ghule et al., 2015), with physical side‐effects presented as key barriers to female use of FP. Another (O. L. McCarthy et al., 2018b) noted the negative impact of **intervention costs**. Two studies (Ahmed et al., 2013; Nair et al., 2019) indicated the importance of **quality of delivery** on intervention outcomes. Five studies commented on **provider‐preparedness** to deliver FP (including provider characteristics), with the trustworthiness, knowledge, and flexibility of providers highlighted as key (Daniele, 2017; Khan et al., 2008; McCarthy et al., 2018b). One study noted challenges in engaging men when healthcare providers were female (Daniele, 2017).

Four studies mentioned participant **recruitment and retention** as potential influences on programme effectiveness, issues around engaging men in couple‐focused sessions or with MCH service settings highlighted as particularly challenging (Daniele, 2017; Harrington, 2017a; Nair et al., 2019; Turan et al., 2001) Further, five studies (Bertrand et al., 1982; Daniele, 2017; Harrington et al., 2019; Jewkes et al., 2010; McCarthy et al., 2018b) highlighted the importance of the specific **characteristics of the study design** as important. One study (Harrington, 2017a) noted contamination across intervention and control communities as a barrier to impact, while the others implicated particular aspects of their programme design (e.g., communications, assertiveness skills session, instant messages) as key. Relatedly, two studies (Bertrand et al., 1982; Ghule et al., 2015), discussed the importance of **reach**. These related to how study processes might ensure that they are able to reach those in the most rural or hard‐to‐reach areas. Finally, two studies (Akhter et al., 1993; Ghule et al., 2015) noted the importance of the **favourable attitudes** towards or satisfaction with the contraceptive method being used.

##### Sensitivity analysis

Only one of the connected papers was deemed to have a high risk of bias (McCarthy, 2019). This study contributed data to two of the themes (co‐residence with extended family and gender norms). When we removed it from the analysis the impact was considered negligible. This was because both themes were supported by evidence from several other studies.

## 6 DISCUSSION

### 6.1 Summary of main results

Our meta‐analysis of 72 studies and 265 measures of effects of interventions involving men and boys in FP found that, when compared with comparison groups, these interventions exert a moderate, yet statistically significant, positive impact on contraceptive use. The reader should note that this analysis does not compare interventions that involve men and boys *with those that do not*. Studies included in this review demonstrate effectiveness in increasing contraceptive use when they are compared to comparison groups, which received a range of interventions including no intervention (care as usual) and alternative interventions, which may also have included men and boys.

While the impact of these interventions on contraceptive use was clear, the causal chain was not. We found that across the range of proximal and distal outcome measures (including contraceptive use, desired family size, pregnancy, pregnancy timing, gender equitable attitudes, communication about FP, equitable decision‐making about FP, attitudes about FP, knowledge about contraceptives, and FP service use) there were few clear or consistent findings. Individual studies produced a mix of results, using highly heterogeneous interventions, composed of several components, and implemented in a variety of contexts, among diverse populations.

Our analysis revealed that the high heterogeneity of effects among included studies was mostly due to *within* study variability. We therefore sought to uncover the effective characteristics and combinations of characteristics of included interventions. However, such was the variability in relation to outcome measures used, that our analysis of 33 interventions including a male engagement component and contraceptive use outcome was only powered to detect the moderating potential of two intermediate outcome measures (*contraceptive attitudes* and *contraceptive knowledge*) and it emerged that only *attitudes about contraception* had a significant moderating impact on contraceptive use outcomes.

The next step in our analysis involved examining the effective components of included studies. Multi‐variate meta‐regression of 72 included studies that reported contraceptive use outcomes indicated that none of the eight intervention components identified were statistically significantly more effective than the others in improving contraceptive use. This is perhaps not surprising given that all interventions included multiple components. We found 33 different combinations of components in use across the 72 studies reporting contraceptive use outcomes and none of the combinations emerged as statistically significantly more effective than the others. When we examined a subset of 33 of the included studies, those involving active engagement of men and boys in FP and the outcome of contraceptive use, we found that the impact of these studies on contraceptive use was not statistically significantly better than those that merely involved men as programme recipients. While at first glance these findings may appear frustratingly inconclusive, and perhaps of little value to programme planners, the findings indicate that a number of different components and combinations of components are possible and effective. This review presents the first collated categorisation of existing interventions involving men and boys in family planning and their components. Given the variety of contexts in which such interventions are implemented, and the recognition that one size does not fit all, this range of possibilities is welcome.

The moderator analysis was able to disentangle some of the complexity relating to the characteristics of included interventions, with clear indications emerging regarding promising methods of engaging programme participants and approaches to implementing interventions that involve men and boys. Meta‐regression across 72 studies highlighted the positive impact of community‐based educational FP interventions, delivered to women as well as men, by trained facilitators, professionals, or peers in community, home and community, or school settings. Programmes that primarily involved contraceptive counselling did not statistically significantly moderate effects on contraceptive use and the analysis was not powered to detect the impact of maternal and child health focused programmes. The evidence also supported approaches targeting adolescents or adults alone, as well as those that targeted both age groups. In contrast to the findings of previous research (Lopez et al., 2009) our analysis found that both interventions based on an explicitly named theory of change and those not reported to be based on a theory of change were effective in positively impacting contraceptive use.

In relation to dosage, the analysis suggests that both short interventions of less than 3 months and those of 7 months or longer are effective moderators of positive impacts on contraceptive use, however, interventions of intermediate timeframes of between 3 and 6 months did not show statistically significant effects. While this finding may appear peculiar, and is difficult to explain definitively, it is possible that content‐ and behaviour‐related issues are at play. For example, shorter interventions generally target simpler behaviours, while longer interventions tend to target more complex behaviours over a longer period. One possible explanation for this finding is that some mid‐length interventions may attempt to address complex behaviours in too short a timeframe.

The analysis suggested that the field of involving men and boys has improved in relation to its capacity to show impact in the last twenty years, with studies published from 2000 onwards emerging as positive moderators of impact. The results also suggest that there may be lessons to learn from programmes implemented in Western and Eastern Africa where positive effects were statistically significantly more pronounced. The reader should note, however, that given the diversity of studies included and the lack of studies in some regions, the analysis was not powered to detect the impact of studies conducted in some regions including South and Central America, Central Asia, and Central Africa. Further, this analysis does not compare interventions that involve men and boys *with those that do not*. Studies included in this review demonstrate effectiveness in increasing contraceptive use when they are compared to comparison groups, which received a range of interventions including no intervention (care as usual) and alternative interventions, which may also have included men and boys.

Our qualitative syntheses of findings from 23 connected qualitative and process evaluation papers related to 34 male engagement studies with a contraceptive use outcome revealed several potential barriers and facilitators of effective models of FP involving men and boys. Central here were system‐ and process‐level barriers and facilitators that echoed findings reported in the quantitative synthesis including the importance of promoting positive attitudes about contraceptives, involving trained peers as programme facilitators, and the value of community‐based educational programmes.

Reflecting the finding relating to the importance of attitudes about contraceptives as a moderator of contraceptive use, the connected papers reported the facilitative effect of changing attitudes of not only individual men and women, but also those of the wider social network, including family members and peers. Repeatedly, social norms and expectations that encouraged early childbearing, preferences for sons, encouragement of male dominance in decision‐making and stigmatisation of their engagement in FP, were highlighted as barriers. Conversely, and reflective perhaps of the positive effects of peer facilitated and community‐based interventions, the positive attitudes of the wider family, peers and community relating to FP were reported as key facilitators.

Though knowledge was not a significant outcome in the quantitative analysis, the crucial importance of accurate knowledge about FP as a facilitator was highlighted in the connected papers, with some studies noting the mediating effect of knowledge of communication about FP. Further, the inclusion of an ‘information and education’ component in almost all included interventions and the moderating effect of interventions focused on a community‐based educational model was reflective of this. In addition, the positive impacts of using trained facilitators or peers and professionals to deliver interventions may reflect the key goal of interventions to impart accurate knowledge about FP.

Communication between couples, joint FP decision‐making, and perceptions of gender and cultural norms emerged as important facilitators in the connected papers, although components specifically targeting these outcomes and reports of their measurement appeared much less frequently than attitudinal and behaviour level contraceptive use or pregnancy‐related outcomes. Equally, the focus on pregnancy related outcomes and female contraceptive use suggests that many of the included FP interventions see men as facilitators of women's contraceptive use rather than FP users themselves.

Key facilitators of FP use were socioeconomic factors including older life stage, women's employment outside the home, and men's education level.

#### 6.1.1 Revised logic model

Based on the available evidence and the input collected during our stakeholder meeting, we revised the initial review logic model in the following ways (see Figure 17):

- All information that was not evidenced (i.e., not significant or not included) in the included evaluation studies and connected papers was changed from black to grey font to highlight areas for future research to consider.
- Intervention component headings were changed to reflect more appropriately terms used in the literature. In particular, ‘gender dialogue’ was changed to ‘gender transformative’; information was changed to ‘information and education’, ‘skills‐building and problem‐solving’ were combined, ‘social support’ was changed to ‘social/peer mentor support’, ‘incentivisation’ was changed to ‘subsidisation’ and incentivisation’, ‘Communication’ was changed to ‘Communication about FP’ and ‘male involvement’ was changed to ‘male engagement’. Additionally, ‘free contraceptives’ was added to ‘subsidisation' and incentivisation’ and ‘subsidised or free FP methods’ removed from ‘health service enhancement’.
- Under intervention characteristics only evidence‐based characteristics after the sub‐headings were left in place. The ‘why’ and ‘tailoring & modifications’ headings were removed. The remaining elements that were not reported or evidenced were changed to grey coloured font.
- Under potential negative outcomes ‘male resistance to FP leading to covert use and unmet need’ was added and the remaining items which were not reported were changed to grey font.
- Under process metrics, reach and favourability of contraceptives were added.
- For the remaining sections, all information not reported was greyed out and information reported left in place.

### 6.2 Overall completeness and applicability of evidence

We followed a pre‐registered peer‐reviewed protocol that was developed in consultation with expert stakeholders and methods experts. A comprehensive search was conducted to identify relevant studies and two reviewers worked independently to select studies using the predetermined eligibility criteria and extract outcome data using a standardised data extraction form. To the best of our knowledge, the evidence presented in this review represents the totality of experimental and quasi‐experimental research from LMICs on the impacts of FP interventions involving men and boys on FP outcomes. We include the broadest range of information available on the nature, extent, and characteristics of experimental evidence in this field from 127 experimental and quasi‐experimental evaluation studies and 23 connected process evaluations and qualitative studies relating to a subset of 33 of the evaluation studies. The large number of studies included in this review meant that data extraction and analysis were more time consuming than expected (*searches were conducted in August 2020*). However, given the slow‐moving nature of publication in this field, the review is timely and, therefore, applicable to those involved in the current development and implementation of FP interventions involving men and boys in FP in LMICs.

Some possible limitations on the applicability of the findings should be noted. First, as noted above this analysis does not compare interventions that involve men and boys *with those that do not*. We also noted that most of the included studies targeted older adolescents and men, so these findings cannot be reliably applied to younger adolescents or children. Country range in the included evaluation studies was narrow within regions with fewer studies from the Americas and Asia than from Africa. Further, as we did not conduct analyses regarding urban and rural settings for intervention delivery, it was not possible to conclude whether findings from rural areas are applicable to urban areas and vice‐versa. Given potential differences in these settings and the implications for intervention implementation, for example, some interventions may require the availability of facilities only present in urban areas or community networks only evident in rural areas, this is an important consideration.

We also found that there was an absence of comparable measures for some outcomes that may be of particular interest to some practitioners. For example, some studies used bespoke measures of gender equitable behaviours and beliefs and joint‐decision‐making about FP, making it difficult to explore these important outcomes in‐depth. This is particularly relevant when we consider the potential for FP interventions that involve men and boys to negatively impact the already skewed existing power dynamics. Understanding, for example, what ‘gender‐equitable decision‐making’ involves and ensuring consistent measurement across studies would allow us to scrutinise and work to eliminate any possible adverse effects. There was also a lack of data on more distal outcomes such as pregnancy, birth spacing, fertility rates, and met need for FP and related outcomes such as intimate partner violence. This may equally be considered a limitation of the current review, and of the extant evidence in the area.

Finally, as noted, the included studies were highly heterogeneous in nature, combining a range of components and characteristics. Although we endeavoured to disentangle these, we ultimately found too many different combinations of characteristics and components to clearly determine the causal chain. We have, however, made progress in this regard and we anticipate that the detailed results and revised review logic model may provide much needed clarity in relation to promising practices for engaging men and boys in FP in ways that promote health and wellbeing for both women and men.

### 6.3 Quality of the evidence

Risk of Bias analyses indicated that 69 of the 127 studies (54%) had a high risk of bias with serious concerns, while 5 studies (4%) had a critical risk of bias. Fifty‐two studies (41%) were accessed as having a moderate risk of bias with some concerns, and only one study was determined to have a low risk of bias. The majority of the 15 process evaluations and 8 qualitative studies were judged to have moderate risk of bias (78%) while only one study was judged to have a high risk of bias and four studies (17%) to have a low risk of bias.

The risk of bias findings should not necessarily be considered indicative of poor study design quality, rather that the majority were conducted in challenging contexts, which affected the proper implementation of the study. The LMIC settings in which these studies were conducted might in part contribute to the need to take pragmatic steps to improve implementation and evaluation. This finding is reminiscent of a previous review of male engagement and sexual and reproductive health and rights interventions published by some of the study authors (Ruane‐McAteer et al., 2020). Forty percent of the studies included in this review were RCTs, with the remaining using quasi‐experimental designs. While some might argue that quasi‐experimental studies are potentially lower in quality than the ‘gold‐standard’ RCT, it should also be noted that quasi‐experimental studies are often a valid alternative in contexts in which ethical or resource constraints prevent the use of RCTs (Thomas, 2016).

### 6.4 Limitations and potential biases in the review process

Our inclusion criteria led to a larger than expected number of included studies. Our decision, due to resource restraints, to focus some of the analysis on a subset of studies (namely first those that included a contraceptive use outcome (72 studies) and second those that had an active male engagement component and contraceptive use outcomes (33 studies)) reduced the number of studies and associated effect sizes that could be examined as part of the meta‐analysis, limiting our ability to examine some elements of the causal chain in more depth. Further, the use of a sample of associated connected process evaluations and qualitative studies instead of a full search for all relevant papers in the field may have biased our findings.

We included all contraception use in the contraceptive use outcome. This included the very small number of studies that included withdrawal as a method of contraception. We used systematic review process methods to minimise bias during the review process. As noted, a deviation from protocol was implemented in relation to dual extraction of data relating to study characteristics, intervention characteristics, and risk of bias assessments. Although this process introduces the possibility of bias, we are confident that the reliability of this approach is in line with accepted standards (Landis & Koch, 1977; McHugh, 2012).

As noted, the analysis does not compare interventions that involve men and boys with those that do not. There was large heterogeneity in the comparison groups of the studies. Also, as noted only studies that included a comparison group were included in this systematic review. This has the potential to exclude important evaluations conducted by practitioners (e.g., before‐after evaluations), which are often done without comparison groups. Given the expense involved in conducting controlled experiments, and the fact that this study focused on low resource settings, this could be considered a limitation. However, from the perspective of methodological rigour, RCTs and quasi‐experimental studies with a comparison group ensure assessment of the effectiveness of interventions.

While information was available in most studies to calculate effect sizes, this was not always the case. The study team contacted authors to request additional data to facilitate this, however, no authors responded with the required information. It is possible that this resulted in biased findings. Also, we did not extract data on the urban/rural breakdown, a limitation of our review, considering that there was some evidence that this distinction is important.

Moderator analyses are exploratory in nature and should always be interpreted with caution (Borenstein et al., 2009). Additionally, these types of analyses generally have low statistical power owing to missing data in the primary research due to the incomplete reporting of many of the variables of interest. Analyses are restricted considerably due to this issue and robust conclusions from these analyses are constrained.

### 6.5 Agreements and disagreements with other studies or reviews

Overall, the findings of this review reinforce and expand the findings from prior research in this field. Our finding relating the effectiveness of FP interventions involving men and boys confirm and expand on those of a review by Phiri and colleagues (2015a), which involved a narrative synthesis of findings from ten randomised controlled trials. Building on findings of prior reviews conducted by members of the current review team (Robinson et al., 2021; Ruane‐McAteer et al., 2019, 2020) and others (Sahay et al., 2021), our review uncovers the complexity of characteristics and components of a subset of interventions that involve male engagement in SRHR, adding multivariate statistical analyses to help uncover effective characteristics. Our findings of the significant positive effect of the following intervention characteristics, namely, multi‐component community based educational interventions; interventions targeted to both males and females; and interventions of longer duration (at least seven months) delivered by professionals and or trained facilitators or peers are consistent with those identified in a review of engaging men and boys in gender‐transformative SRHR interventions (Ruane‐McAteer et al., 2020). In addition, however our review has identified that brief interventions of less than three months in the field of family planning also demonstrate effectiveness. In support of findings of a recent review from Sahay and colleagues (2021) and an analysis of the FP2020 commitments made by several LMICs in relation to involving males in FP programmes (Hook et al., 2021), this review confirms the importance of improving knowledge and attitudes related to contraception as a means of increasing its uptake and use. Further, in common with the evidence and gap map of engaging men and boys relating to all SRHR outcomes (Ruane‐McAteer et al., 2019), we found that addressing gender inequitable norms was not ubiquitous among these programmes. While in their map, they estimated that only 8% of evaluated male engagement interventions across all SRHR outcomes included a gender‐transformative approach, we estimated that 23% of the evaluated interventions in this review of FP interventions adopted gender‐transformative components. Likewise, both reviews identified that few interventions that have been evaluated using experimental methods include broader structural components.

Our finding that both interventions with and without a clearly specified theory of change are effective moderators is different to a previous review by Lopez et al. (2009) which found theory‐based interventions to be more effective. It is important to note, however, that although some of our included studies did not clearly indicate that the intervention was based on a theory of change, it is possible that the theory was not reported or recognised. Indeed, the use of behaviour change theory in FP programmes is argued to be under reported and under detailed (Robinson et al., 2021). While the incorporation of explicit theoretical grounding may serve to advance the field, this may not be sufficient in isolation, with calls for evidence‐led programme development also (Raj et al., 2016). These results indicate successful programme development and implementation may therefore be theory‐ or data‐driven, and prompt recommendation that both approaches be incorporated.

## 7 AUTHORS CONCLUSIONS

Family planning interventions that involve men and boys alongside women and girls are effective in improving uptake and use of contraceptives. Programmers across the world have developed and evaluated a wide range of interventions, as rich and varied as the contexts in which they are delivered. This variability, while necessary to some degree, also has implications for evidence synthesis. Heterogeneity of components, characteristics and outcomes meant that some meta‐analyses were not possible with the current data set. This review did, however, unravel some parts of the causal chain, highlighting effective characteristics of existing interventions, and determining that there was no significant difference in the size of effect of eight different components or combinations of components on contraceptive use. The implications of this for practice and research are outlined below.

### 7.1 Implications for practice and policy

Stakeholder involvement was central in this project, with an international advisory group of more than 30 expert members from 9 different countries around the world participating in planning the review, developing the initial and revised versions of the review logic model, interpreting the implications for findings, and disseminating evidence.

The evidence suggests that existing effective interventions should be adapted and implemented across LMICs where there is unmet need for family planning. While approaches to involving men and boys in family planning are complex, the research indicates that practitioners should utilise multi‐component interventions and can choose from a variety of different components depending on the population and setting in which it will be delivered. The mixed methods evidence examined in this review suggests that programme planners should consider the following points when seeking out, adapting, evaluating, and implementing interventions:

1. **Promote gender equity in FP** and reduce the negative impact of harmful masculinities by involving women as well as men in programmes and implementing interventions that use gender transformative elements. Programmes can do this by facilitating communication about FP and joint FP decision‐making among couples, promoting female empowerment to decide on FP method use, and encouraging men as supporters of FP, but also users of FP in their own right. There is also a role for practitioners in reducing stigma around the involvement of men in FP and providing FP services that welcome men as well as women.
2. **Harness the power of positive role models** by empowering trained peer mentors and trained facilitators and professionals to implement culturally adapted interventions. Use community‐based educational approaches that improve accurate knowledge and positively change attitudes and FP among the wider social network in order to positively impact restrictive social and cultural norms.
3. **Consider the use of multi‐component, multi‐level interventions** adapted and matched to meet local needs and addressing relevant system‐ and process‐level barriers to effective FP intervention. This review identified the use of eight different components and 33 different combinations of these that were effective moderators of impact on contraceptive use. While all of the different combinations of components were effective in moderating contraceptive use, none of the different combinations stood out as more effective than the others. This list should not be considered exhaustive and creative ways of developing and implementing new components and combinations of components are encouraged. While the evidence presented here suggests that interventions based on a clearly identified behaviour‐change theory have no more impact on contraceptive use outcomes then those that do not, we recommend that practitioners consider theories of change as a fundamental aspect of programme planning.
4. **Addressing socioeconomic inequalities**. Policy that aims to improve women's education and opportunities for employment outside the home as well as the provision of free or subsidised FP services and contraception would go some way towards encouraging uptake of FP and addressing unmet need in LMICs.
5. **Carefully consider proximal and distal outcomes**. The evidence presented in this review revealed a gap in interventions that move beyond the interpersonal level to impact community, organisation/service, and structural level outcomes. There is also a need to consider and measure the longer terms impacts of FP interventions and more uniform methods of outcome measurement. This will facilitate drawing conclusions on the effectiveness of FP approaches in future.

### 7.2 Implications for research

The analysis identified some gaps in evidence in relation to our review questions that have implications for future research.

First, in relation to the populations under study, few studies were available from South and Central America, the Middle East, and Northern Africa. Within regions, research tended to be focused on particular countries, with only 17 LMICs represented in the review. Given the importance of local cultural norms as barriers or facilitators of uptake of FP, much more evaluation research is needed internationally, with research funds targeted at countries in which unmet need for FP and robust evidence is lacking. It would also be valuable for future reviews to collate data on whether studies are conducted in urban or rural settings. Data collection for this study occurred during the COVID‐19 pandemic so we examined the data for studies that took place during disease outbreaks. We found none. Equally, we found no studies that took place in conflict, disaster, or climate stressed contexts. Given the continued impact of these factors across the world and their potential implications for increasing unmet need for FP, further research in these settings is urgently needed.

In relation to intervention and study characteristics, we found that reporting within studies was variable, with many studies not following recommended reporting guidelines. We extracted *PROGRESS Plus* criteria (O'Neill et al., 2014) when it was available but these details were too sporadically reported to include in the analysis. Similarly, some studies provided insufficient or unclear information on intervention characteristics, with a variety of terms used for the same components. This made it very difficult to code and categorise data. Future intervention evaluation studies should use recognised behaviour change terminology such as that proposed by Michie et al. (2011) and also ensure to use appropriate intervention reporting guidelines such as TiDiER.

On an outcome level there were few studies that examined interventions delivered beyond the individual or interpersonal levels. There is much room for programme planners and evaluators to intervene as these levels as recommended elsewhere (Ruane‐McAteer et al., 2020). Further, none of the included studies reported the use of participatory designs, an approach that is recommended for future work to ensure the relevance of intervention and study designs for particular contexts. More research is also needed on intervention designs based on incorporating male involvement in FP with maternal and child health programmes. Some studies included reported promising results, but the studies were too few to conduct meaningful analysis.

A related recommendation for researchers relates to how outcomes are measured across included studies. We established high heterogeneity in relation to how outcomes were measured across different studies, using different assessment methods, outcome measures, timings and methods of reporting. Very few studies distinguished between primary and secondary outcomes. This makes synthesis and meta‐analysis of results challenging. Research using standardised measures is highly recommended and reporting of experimental studies should follow the CONSORT checklists.

A further recommendation relates to the absence of economic evaluations of these interventions. Only three of the included studies (Bertrand et al., 1982; Diop et al., 2004; Townsend et al., 1987) examined cost‐effectiveness, so research exploring this important factor is urgently needed.

A final methodologically focused recommendation relates to the development and adaptation of interventions and conduct of experimental research in this field. As noted, we faced significant challenges in our attempts to unpack the causal chain, in part because of the complex nature of the included interventions. This has implications for future development and evaluations in this field. While FP interventions that involve men and boys include an ‘intervention package’ that consists of multiple components and that may independently contribute (either positively or negatively) to overall effects, it is extremely difficult to understand the individual and combined impact of different components using classical experimental methods. Further research should therefore consider alternative methods that are capable of uncovering the individual and combined effects of intervention components. One such approach is the Multiphase Optimisation Strategy (MOST) (Collins, 2018), which involves a three‐phase process to prepare, optimise (using factorial experimental designs) and evaluate complex behavioural interventions. None of the studies included in this review included this approach.

The legacy of a focus on population control and global patriarchal norms has undeniably influenced the state of current FP interventions, which centre around birth limiting and birth spacing and women's central role as contraceptive users. While we intended to study unmet FP need, we found that the most common included outcome across all studies was contraceptive use and unmet family planning need was rarely studied. Areas and topics of interest for future FP interventions should include engaging men as contraceptive users, supporters, and agents in helping to achieve desired family size but also in fertility promotion interventions, safe conception interventions (i.e., HIV positive conception), and family planning decision making in non‐heterosexual relationships.

Similarly, all interventions included in this review adhered to binary and cis‐normative concepts of gender identity and sexuality. Family planning remains a pertinent issue for those identifying as LGBTQI+, with authors noting that even those who have transitioned socially or hormonally are in need of support to ensure they can achieve their desired family size (Francis et al., 2018). The experiences of transgender individuals remain critically under‐investigated in relation to family planning, hence given the novel and unmet need for this group further research is called for into the need to involving transgender men in family planning.

Finally, notably absent from the interventions included in this review were behavioural interventions that support those who do not ever wish to become parents. Given the reported pressures placed on young couples to engage in childbearing noted in this review and increasing trends of individuals deciding to delay or avoid parenthood (Mauceri & Valentini, 2010; Nomaguchi & Milkie, 2020; Umberson et al., 2010), it is likely that this subgroup of people represent a significant yet neglected population that deserve the attention of future research.

## CONTRIBUTIONS OF AUTHORS

Principal Investigators: ÁA, ML

Expert advice and guidance: MC, MT, FO, CB

Searches and Screening: MR, JeH, JaH, ÁA, ML

Data Extraction: MR, JaH, ER, ÁA,

Content: ÁA, ML, MR, ER, CB

Analysis: MR, CK, JeH, ÁA, ML, ER

Synthesis: ÀA, MR, JeH, ML

Dissemination: FO, MT, ÁA, ML

## DECLARATION OF INTEREST

This project was funded by the Centre of Excellence for Development Impact and Learning (CEDIL) with support from UK Aid from the UK government. The funding body had no role in the study design, or decision to publish findings. The authors have no conflicts of interest to declare.

## PRELIMINARY TIMEFRAME

The preliminary timeframe for submission of the completed review was one year following protocol publication. This was delayed by two months (the protocol was published in January 2021 and the completed review submitted in March 2022).

## PLANS TO UPDATE THIS SYSTEMATIC REVIEW

The authors seek support to update the results of this review in line with emerging evidence in the field. We anticipate that the need for the next update will be considered in 5 years.

## DIFFERENCES BETWEEN PROTOCOL AND REVIEW

Deviations from the review protocol are presented in Section 4.2.1.

## PUBLISHED NOTES

None.

## Supporting information

Supporting information.
