# Supplementary material for: Involving men and boys in family planning: A systematic review of the effective components and characteristics of complex interventions in low‐ and middle‐income countries
Source: Campbell Syst Rev. 2023 Jan 13;19(1):e1296. doi: 10.1002/cl2.1296 (PMC9837728; doi:10.1002/cl2.1296)
Supplement: Supplementary file 1 — Supporting information. [file CL2-19-e1296-s001.docx]

# 7.0 Appendices

### Appendix 1.0: Initial Review Logic Model


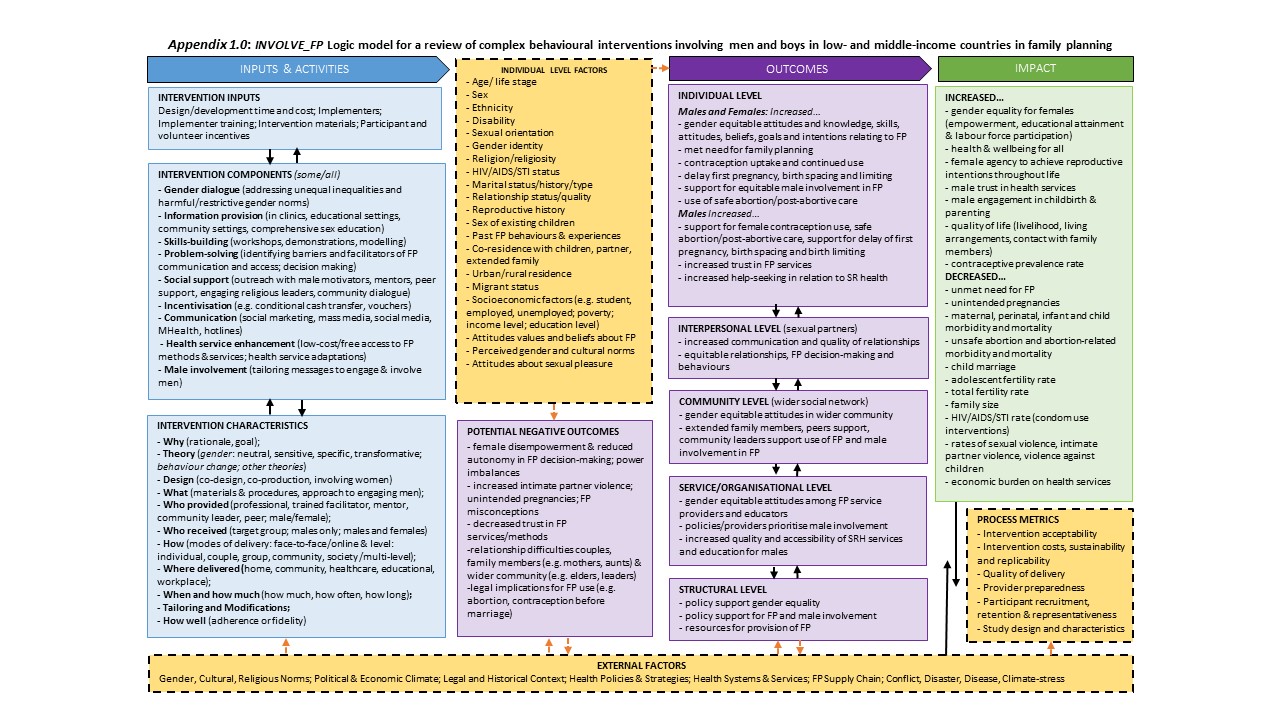


### Appendix 2.0 Search and Development Testing

The search was developed and tested in Medline. Searches were tested and adjusted as necessary to account for the unique indexing, field codes and truncation for each database.

Population: men and boys

Men or man or male or males or boy or boys or masculin* or father* or husband.ti,ab,fx,kf,hw.

This search string was adapted from Ruane‐McAteer et al., (2018) by adding “husband”. The addition of “partner” and gender equality terms was tested but these did not add unique relevant records and added irrelevant records and were removed.

Condition of interest: Family Planning

Family planning or ((unintended or unwanted or unplanned or planned or wanted or intended) ADJ Pregnan*) or Contracepti* or birth control or adolescent pregnancy or birth spacing or birth interval* or child spacing or pregnancy interval* or delay pregnancy or abortion or abortions).ti,ab,fx,kf,hw.

This string was adapted from Ruane-McAteer et al. 2018 and Robinson et al. (in process). Each term was tested using Mesh headings to identify relevant overlapping concepts and terms. Each potential term and varitants of the term (e.g. abortion, abort*, abortion or abortions) was tested to ensure it added unique records not captured by other terms using NOT. Only terms that added unique records were included in the final string.

We also discussed and tested the addition of terms relating to fertility and infertility. The team concluded that, as the review is focused on family planning in the sense of preventing unintended pregnancy we decided not to include specific search terms for fertility or infertility. We felt that we could not do justice to considerations around infertility treatment within the context of this review and we believe that it warrants its own review. This is particularly because men are so often left out of discourses on fertility which is damaging for both men and women as it shifts focus and responsibility for reproduction, and by extension child rearing, solely to women.

Study Design

randomized controlled trial or controlled clinical trial or pragmatic clinical trial or multicenter study).pt.

non-randomized controlled trials as topic/

interrupted time series analysis/

controlled before-after studies/

(randomis* or randomiz* or randomly).ti,ab.

groups.ab.

(trial or multicenter or multi center or multicentre or multi centre).ti.

(intervention? or effect? or impact? or controlled or control group? or (before adj5 after) or (pre adj5 post) or ((pretest or pre test) and (posttest or post test)) or quasiexperiment* or quasi experiment* or evaluat* or time series or time point? or repeated measur* or ((nonequivalent or non equivalent) adj3 control*)).ti,ab.

(program* and evaluat*).ti,ab,kw.

This string was developed by adapting the sample string produced by Cochrane EPOC (2017) adding (((nonequivalent or non equivalent) adj3 control$) and (program* and evaluat*).ti,ab,kw which added at least 3 potentially relevant records not otherwise captured.

Low- and Middle-Income Countries

(afghanistan or albania or algeria or american samoa or angola or "antigua and barbuda" or antigua or barbuda or argentina or armenia or armenian or aruba or azerbaijan or bahrain or bangladesh or barbados or republic of belarus or belarus or byelarus or belorussia or byelorussian or belize or british honduras or benin or dahomey or bhutan or bolivia or "bosnia and herzegovina" or bosnia or herzegovina or botswana or bechuanaland or brazil or brasil or bulgaria or burkina faso or burkina fasso or upper volta or burundi or urundi or cabo verde or cape verde or cambodia or kampuchea or khmer republic or cameroon or cameron or cameroun or central african republic or ubangi shari or chad or chile or china or colombia or comoros or comoro islands or iles comores or mayotte or democratic republic of the congo or democratic republic congo or congo or zaire or costa rica or "cote d’ivoire" or "cote d’ ivoire" or cote divoire or cote d ivoire or ivory coast or croatia or cuba or cyprus or czech republic or czechoslovakia or djibouti or french somaliland or dominica or dominican republic or ecuador or egypt or united arab republic or el salvador or equatorial guinea or spanish guinea or eritrea or estonia or eswatini or swaziland or ethiopia or fiji or gabon or gabonese republic or gambia or "georgia (republic)" or georgian or ghana or gold coast or gibraltar or greece or grenada or guam or guatemala or guinea or guinea bissau or guyana or british guiana or haiti or hispaniola or honduras or hungary or india or indonesia or timor or iran or iraq or isle of man or jamaica or jordan or kazakhstan or kazakh or kenya or "democratic people’s republic of korea" or republic of korea or north korea or south korea or korea or kosovo or kyrgyzstan or kirghizia or kirgizstan or kyrgyz republic or kirghiz or laos or lao pdr or "lao people's democratic republic" or latvia or lebanon or lebanese republic or lesotho or basutoland or liberia or libya or libyan arab jamahiriya or lithuania or macau or macao or "macedonia (republic)" or macedonia or madagascar or malagasy republic or malawi or nyasaland or malaysia or malay federation or malaya federation or maldives or indian ocean islands or indian ocean or mali or malta or micronesia or federated states of micronesia or kiribati or marshall islands or nauru or northern mariana islands or palau or tuvalu or mauritania or mauritius or mexico or moldova or moldovian or mongolia or montenegro or morocco or ifni or mozambique or portuguese east africa or myanmar or burma or namibia or nepal or netherlands antilles or nicaragua or niger or nigeria or oman or muscat or pakistan or panama or papua new guinea or new guinea or paraguay or peru or philippines or philipines or phillipines or phillippines or poland or "polish people's republic" or portugal or portuguese republic or puerto rico or romania or russia or russian federation or ussr or soviet union or union of soviet socialist republics or rwanda or ruanda or samoa or pacific islands or polynesia or samoan islands or navigator island or navigator islands or "sao tome and principe" or saudi arabia or senegal or serbia or seychelles or sierra leone or slovakia or slovak republic or slovenia or melanesia or solomon island or solomon islands or norfolk island or norfolk islands or somalia or south africa or south sudan or sri lanka or ceylon or "saint kitts and nevis" or "st. kitts and nevis" or saint lucia or "st. lucia" or "saint vincent and the grenadines" or saint vincent or "st. vincent" or grenadines or sudan or suriname or surinam or dutch guiana or netherlands guiana or syria or syrian arab republic or tajikistan or tadjikistan or tadzhikistan or tadzhik or tanzania or tanganyika or thailand or siam or timor leste or east timor or togo or togolese republic or tonga or "trinidad and tobago" or trinidad or tobago or tunisia or turkey or "turkey (republic)" or turkmenistan or turkmen or uganda or ukraine or uruguay or uzbekistan or uzbek or vanuatu or new hebrides or venezuela or vietnam or viet nam or middle east or west bank or gaza or palestine or yemen or yugoslavia or zambia or zimbabwe or northern rhodesia or global south or africa south of the sahara or sub-saharan africa or subsaharan africa or africa, central or central africa or africa, northern or north africa or northern africa or magreb or maghrib or sahara or africa, southern or southern africa or africa, eastern or east africa or eastern africa or africa, western or west africa or western africa or west indies or indian ocean islands or caribbean or central america or latin america or "south and central america" or south america or asia, central or central asia or asia, northern or north asia or northern asia or asia, southeastern or southeastern asia or south eastern asia or southeast asia or south east asia or asia, western or western asia or europe, eastern or east europe or eastern europe or developing country or developing countries or developing nation? or developing population? or developing world or less developed countr* or less developed nation? or less developed population? or less developed world or lesser developed countr* or lesser developed nation? or lesser developed population? or lesser developed world or under developed countr* or under developed nation? or under developed population? or under developed world or underdeveloped countr* or underdeveloped nation? or underdeveloped population? or underdeveloped world or middle income countr* or middle income nation? or middle income population? or low income countr* or low income nation? or low income population? or lower income countr* or lower income nation? or lower income population? or underserved countr* or underserved nation? or underserved population? or underserved world or under served countr* or under served nation? or under served population? or under served world or deprived countr* or deprived nation? or deprived population? or deprived world or poor countr* or poor nation? or poor population? or poor world or poorer countr* or poorer nation? or poorer population? or poorer world or developing econom* or less developed econom* or lesser developed econom* or under developed econom* or underdeveloped econom* or middle income econom* or low income econom* or lower income econom* or low gdp or low gnp or low gross domestic or low gross national or lower gdp or lower gnp or lower gross domestic or lower gross national or lmic or lmics or third world or lami countr* or transitional countr* or emerging economies or emerging nation?).ti,ab,sh,kf.

We used the search string developed and tested by Cochrane EPOC (EPOC LMIC filters 2020 (v.3)) retrieved from <https://epoc.cochrane.org/lmic-filters> on 29th June 2020. These filters are based on the World Bank list of countries (2019), classified as low-income, lower-middle-income or upper-middle-income economies and were prepared by Cochrane Effective practice and organisation of care group.

Intervention

Given the very broad range of potential interventions we did not to limit the search by intervention terms in the initial stages. We developed this search string as follows:

1. Search for the combination of the terms for population AND family planning AND study design AND LMIC in two databases (psych info and medline).
2. Scan the first 200 records retrieved in each database to quickly identify studies that appear to meet our eligibility criteria (400 records screened).
3. We used this selection of studies to develop and test a comprehensive list of intervention terms.
4. We will then screened a further selection of 200 records in each database to identify a new set of potentially eligible studies. This new set was then used to verify that the newly developed string captures the second set of potentially eligible studies and did not exclude any potentially relevant study.
5. The first set of intervention terms failed to capture one potentially relevant study identified in step 4. The intervention term list was expanded to capture the relevant term (in this case training) and the process above was repeated once more. All relevant records were identified in the next round. We were therefore satisfied that adding intervention terms improved search specificity without adversely affecting sensitivity.

**Intervention terms**

(educat* or behav* or peer or community or psycho* or social* or Counselling or counseling or media or communication or marketing or provision or provide or provided or delivery or delivered or deliver or delivery or delivered or deliver or distribution or distributed or distribute or train or training or trained or mhealth or program* or intervention*).mp. [mp=title, abstract, original title, name of substance word, subject heading word, floating sub-heading word, keyword heading word, organism supplementary concept word, protocol supplementary concept word, rare disease supplementary concept word, unique identifier, synonyms]

Search Results:

Database: Ovid MEDLINE(R) ALL <1946 to August 25, 2020>

4874 - 24 duplicates removed by endnote = 4850 unique records

Search Strategy:

1 (Men or man or male or males or boy or boys or masculin* or father* or husband).ti,ab,fx,kf,hw. (9027690)

2 (Family planning or ((unintended or unwanted or unplanned or planned or wanted or intended) adj Pregnan*) or Contracepti* or birth control or adolescent pregnancy or birth spacing or birth interval* or child spacing or pregnancy interval* or delay pregnancy or abortion or abortions).ti,ab,fx,kf,hw. (196558)

3 (afghanistan or albania or algeria or american samoa or angola or "antigua and barbuda" or antigua or barbuda or argentina or armenia or armenian or aruba or azerbaijan or bahrain or bangladesh or barbados or republic of belarus or belarus or byelarus or belorussia or byelorussian or belize or british honduras or benin or dahomey or bhutan or bolivia or "bosnia and herzegovina" or bosnia or herzegovina or botswana or bechuanaland or brazil or brasil or bulgaria or burkina faso or burkina fasso or upper volta or burundi or urundi or cabo verde or cape verde or cambodia or kampuchea or khmer republic or cameroon or cameron or cameroun or central african republic or ubangi shari or chad or chile or china or colombia or comoros or comoro islands or iles comores or mayotte or democratic republic of the congo or democratic republic congo or congo or zaire or costa rica or "cote d’ivoire" or "cote d’ ivoire" or cote divoire or cote d ivoire or ivory coast or croatia or cuba or cyprus or czech republic or czechoslovakia or djibouti or french somaliland or dominica or dominican republic or ecuador or egypt or united arab republic or el salvador or equatorial guinea or spanish guinea or eritrea or estonia or eswatini or swaziland or ethiopia or fiji or gabon or gabonese republic or gambia or "georgia (republic)" or georgian or ghana or gold coast or gibraltar or greece or grenada or guam or guatemala or guinea or guinea bissau or guyana or british guiana or haiti or hispaniola or honduras or hungary or india or indonesia or timor or iran or iraq or isle of man or jamaica or jordan or kazakhstan or kazakh or kenya or "democratic people’s republic of korea" or republic of korea or north korea or south korea or korea or kosovo or kyrgyzstan or kirghizia or kirgizstan or kyrgyz republic or kirghiz or laos or lao pdr or "lao people's democratic republic" or latvia or lebanon or lebanese republic or lesotho or basutoland or liberia or libya or libyan arab jamahiriya or lithuania or macau or macao or "macedonia (republic)" or macedonia or madagascar or malagasy republic or malawi or nyasaland or malaysia or malay federation or malaya federation or maldives or indian ocean islands or indian ocean or mali or malta or micronesia or federated states of micronesia or kiribati or marshall islands or nauru or northern mariana islands or palau or tuvalu or mauritania or mauritius or mexico or moldova or moldovian or mongolia or montenegro or morocco or ifni or mozambique or portuguese east africa or myanmar or burma or namibia or nepal or netherlands antilles or nicaragua or niger or nigeria or oman or muscat or pakistan or panama or papua new guinea or new guinea or paraguay or peru or philippines or philipines or phillipines or phillippines or poland or "polish people's republic" or portugal or portuguese republic or puerto rico or romania or russia or russian federation or ussr or soviet union or union of soviet socialist republics or rwanda or ruanda or samoa or pacific islands or polynesia or samoan islands or navigator island or navigator islands or "sao tome and principe" or saudi arabia or senegal or serbia or seychelles or sierra leone or slovakia or slovak republic or slovenia or melanesia or solomon island or solomon islands or norfolk island or norfolk islands or somalia or south africa or south sudan or sri lanka or ceylon or "saint kitts and nevis" or "st. kitts and nevis" or saint lucia or "st. lucia" or "saint vincent and the grenadines" or saint vincent or "st. vincent" or grenadines or sudan or suriname or surinam or dutch guiana or netherlands guiana or syria or syrian arab republic or tajikistan or tadjikistan or tadzhikistan or tadzhik or tanzania or tanganyika or thailand or siam or timor leste or east timor or togo or togolese republic or tonga or "trinidad and tobago" or trinidad or tobago or tunisia or turkey or "turkey (republic)" or turkmenistan or turkmen or uganda or ukraine or uruguay or uzbekistan or uzbek or vanuatu or new hebrides or venezuela or vietnam or viet nam or middle east or west bank or gaza or palestine or yemen or yugoslavia or zambia or zimbabwe or northern rhodesia or global south or africa south of the sahara or sub-saharan africa or subsaharan africa or africa, central or central africa or africa, northern or north africa or northern africa or magreb or maghrib or sahara or africa, southern or southern africa or africa, eastern or east africa or eastern africa or africa, western or west africa or western africa or west indies or indian ocean islands or caribbean or central america or latin america or "south and central america" or south america or asia, central or central asia or asia, northern or north asia or northern asia or asia, southeastern or southeastern asia or south eastern asia or southeast asia or south east asia or asia, western or western asia or europe, eastern or east europe or eastern europe or developing country or developing countries or developing nation? or developing population? or developing world or less developed countr* or less developed nation? or less developed population? or less developed world or lesser developed countr* or lesser developed nation? or lesser developed population? or lesser developed world or under developed countr* or under developed nation? or under developed population? or under developed world or underdeveloped countr* or underdeveloped nation? or underdeveloped population? or underdeveloped world or middle income countr* or middle income nation? or middle income population? or low income countr* or low income nation? or low income population? or lower income countr* or lower income nation? or lower income population? or underserved countr* or underserved nation? or underserved population? or underserved world or under served countr* or under served nation? or under served population? or under served world or deprived countr* or deprived nation? or deprived population? or deprived world or poor countr* or poor nation? or poor population? or poor world or poorer countr* or poorer nation? or poorer population? or poorer world or developing econom* or less developed econom* or lesser developed econom* or under developed econom* or underdeveloped econom* or middle income econom* or low income econom* or lower income econom* or low gdp or low gnp or low gross domestic or low gross national or lower gdp or lower gnp or lower gross domestic or lower gross national or lmic or lmics or third world or lami countr* or transitional countr* or emerging economies or emerging nation?).ti,ab,sh,kf. (1936228)

4 1 and 2 and 3 (11573)

5 (randomized controlled trial or controlled clinical trial or pragmatic clinical trial or multicenter study).pt. (791622)

6 non-randomized controlled trials as topic/ (741)

7 interrupted time series analysis/ (948)

8 controlled before-after studies/ (541)

9 (randomis* or randomiz* or randomly).ti,ab. (913150)

10 groups.ab. (2086045)

11 (trial or multicenter or multi center or multicentre or multi centre).ti. (266555)

12 (intervention? or effect? or impact? or controlled or control group? or (before adj5 after) or (pre adj5 post) or ((pretest or pre test) and (posttest or post test)) or quasiexperiment* or quasi experiment* or evaluat* or time series or time point? or repeated measur* or ((nonequivalent or non equivalent) adj3 control*)).ti,ab. (9763673)

13 (program* and evaluat*).ti,ab,kw. (200292)

14 5 or 6 or 7 or 8 or 9 or 10 or 11 or 12 or 13 (10875754)

15 exp Animals/ (23390128)

16 Humans/ (18661630)

17 15 not 16 (4728498)

18 14 not 17 (8786728)

19 4 and 18 (5534)

20 educat*.mp. (1037479)

21 behav*.mp. (1724240)

22 peer.mp. [mp=title, abstract, original title, name of substance word, subject heading word, floating sub-heading word, keyword heading word, organism supplementary concept word, protocol supplementary concept word, rare disease supplementary concept word, unique identifier, synonyms] (87318)

23 community.mp. [mp=title, abstract, original title, name of substance word, subject heading word, floating sub-heading word, keyword heading word, organism supplementary concept word, protocol supplementary concept word, rare disease supplementary concept word, unique identifier, synonyms] (568842)

24 psycho*.mp. (1846104)

25 social*.mp. (797852)

26 (Counselling or counseling).mp. [mp=title, abstract, original title, name of substance word, subject heading word, floating sub-heading word, keyword heading word, organism supplementary concept word, protocol supplementary concept word, rare disease supplementary concept word, unique identifier, synonyms] (121031)

27 (media or communication or marketing).mp. [mp=title, abstract, original title, name of substance word, subject heading word, floating sub-heading word, keyword heading word, organism supplementary concept word, protocol supplementary concept word, rare disease supplementary concept word, unique identifier, synonyms] (881001)

28 (provision or provide or provided).mp. [mp=title, abstract, original title, name of substance word, subject heading word, floating sub-heading word, keyword heading word, organism supplementary concept word, protocol supplementary concept word, rare disease supplementary concept word, unique identifier, synonyms] (2069732)

29 (delivery or delivered or deliver).mp. [mp=title, abstract, original title, name of substance word, subject heading word, floating sub-heading word, keyword heading word, organism supplementary concept word, protocol supplementary concept word, rare disease supplementary concept word, unique identifier, synonyms] (745010)

30 (distribution or distributed or distribute).mp. [mp=title, abstract, original title, name of substance word, subject heading word, floating sub-heading word, keyword heading word, organism supplementary concept word, protocol supplementary concept word, rare disease supplementary concept word, unique identifier, synonyms] (1283507)

31 (train or training or trained).mp. [mp=title, abstract, original title, name of substance word, subject heading word, floating sub-heading word, keyword heading word, organism supplementary concept word, protocol supplementary concept word, rare disease supplementary concept word, unique identifier, synonyms] (553019)

32 mhealth.mp. (4952)

33 (program* or intervention*).mp. [mp=title, abstract, original title, name of substance word, subject heading word, floating sub-heading word, keyword heading word, organism supplementary concept word, protocol supplementary concept word, rare disease supplementary concept word, unique identifier, synonyms] (1914006)

34 20 or 21 or 22 or 23 or 24 or 25 or 26 or 27 or 28 or 29 or 30 or 31 or 32 or 33 (9067942)

35 19 and 34 (4874)

***************************

Database: APA PsycInfo <1806 to August Week 3 2020>

Search Results:

580 - 332 = 248 unique records

Search Strategy:

1 (Family planning or ((unintended or unwanted or unplanned or planned or wanted or intended) adj Pregnan*) or Contracepti* or birth control or adolescent pregnancy or birth spacing or birth interval* or child spacing or pregnancy interval* or delay pregnancy or abortion or abortions).ti,ab,sh,hw,id,mh. (21587)

2 (Men or man or male or males or boy or boys or masculin* or father* or husband).ti,ab,sh,hw,id. (672242)

3 randomized controlled trial/ or controlled clinical trial/ or pragmatic clinical trial/ or multicenter study/ (502)

4 clinical trials/ or "treatment outcome clinical trial".md. or ((randomi?ed adj7 trial*) or ((single or doubl* or tripl* or treb*) and (blind* or mask*)) or (controlled adj3 trial*) or (clinical adj2 trial*)).ti,ab,id. (104072)

Annotation: https://libguides.sph.uth.tmc.edu/search_filters/ovid_psycinfo_filters

5 program evaluation/ or educational program evaluation/ or mental health program evaluation/ or ((program development/ or educational program planning/) and (Evaluation Criteria/ or evaluation/ or treatment effectiveness evaluation/ or vocational evaluation/)) or ((pre- adj5 post-) or (pretest adj5 posttest) or (program* adj6 evaluat*)).ti,ab,id. (80815)

Annotation: https://libguides.sph.uth.tmc.edu/search_filters/ovid_psycinfo_filters

6 (intervention or effectiveness).ti,ab,id. (375599)

7 (randomis* or randomiz* or randomly).ti,ab. (149536)

8 groups.ab. (494136)

9 (trial or multicenter or multi center or multicentre or multi centre).ti. (33267)

10 (intervention? or effect? or impact? or controlled or control group? or (before adj5 after) or (pre adj5 post) or ((pretest or pre test) and (posttest or post test)) or quasiexperiment* or quasi experiment* or evaluat* or time series or time point? or repeated measur* or ((nonequivalent or non equivalent) adj3 control*)).ti,ab. (1957407)

11 3 or 4 or 5 or 6 or 7 or 8 or 9 or 10 (2279711)

12 educat*.mp. (653675)

13 behav*.mp. (1257634)

14 (peer or community).mp. [mp=title, abstract, heading word, table of contents, key concepts, original title, tests & measures, mesh] (338255)

15 (psycho* or social*).mp. [mp=title, abstract, heading word, table of contents, key concepts, original title, tests & measures, mesh] (2046178)

16 (Counselling or counseling).mp. [mp=title, abstract, heading word, table of contents, key concepts, original title, tests & measures, mesh] (105472)

17 (media or communication or marketing).mp. [mp=title, abstract, heading word, table of contents, key concepts, original title, tests & measures, mesh] (361408)

18 (provision or provide or provided).mp. [mp=title, abstract, heading word, table of contents, key concepts, original title, tests & measures, mesh] (590720)

19 (delivery or delivered or deliver).mp. [mp=title, abstract, heading word, table of contents, key concepts, original title, tests & measures, mesh] (107705)

20 (distribution or distributed or distribute).mp. [mp=title, abstract, heading word, table of contents, key concepts, original title, tests & measures, mesh] (104844)

21 (train or training).mp. [mp=title, abstract, heading word, table of contents, key concepts, original title, tests & measures, mesh] (294067)

22 mhealth.mp. (996)

23 (program* or intervention*).mp. [mp=title, abstract, heading word, table of contents, key concepts, original title, tests & measures, mesh] (737839)

24 12 or 13 or 14 or 15 or 16 or 17 or 18 or 19 or 20 or 21 or 22 or 23 (3585694)

25 (afghanistan or albania or algeria or american samoa or angola or "antigua and barbuda" or antigua or barbuda or argentina or armenia or armenian or aruba or azerbaijan or bahrain or bangladesh or barbados or republic of belarus or belarus or byelarus or belorussia or byelorussian or belize or british honduras or benin or dahomey or bhutan or bolivia or "bosnia and herzegovina" or bosnia or herzegovina or botswana or bechuanaland or brazil or brasil or bulgaria or burkina faso or burkina fasso or upper volta or burundi or urundi or cabo verde or cape verde or cambodia or kampuchea or khmer republic or cameroon or cameron or cameroun or central african republic or ubangi shari or chad or chile or china or colombia or comoros or comoro islands or iles comores or mayotte or democratic republic of the congo or democratic republic congo or congo or zaire or costa rica or "cote d’ivoire" or "cote d’ ivoire" or cote divoire or cote d ivoire or ivory coast or croatia or cuba or cyprus or czech republic or czechoslovakia or djibouti or french somaliland or dominica or dominican republic or ecuador or egypt or united arab republic or el salvador or equatorial guinea or spanish guinea or eritrea or estonia or eswatini or swaziland or ethiopia or fiji or gabon or gabonese republic or gambia or "georgia (republic)" or georgian or ghana or gold coast or gibraltar or greece or grenada or guam or guatemala or guinea or guinea bissau or guyana or british guiana or haiti or hispaniola or honduras or hungary or india or indonesia or timor or iran or iraq or isle of man or jamaica or jordan or kazakhstan or kazakh or kenya or "democratic people’s republic of korea" or republic of korea or north korea or south korea or korea or kosovo or kyrgyzstan or kirghizia or kirgizstan or kyrgyz republic or kirghiz or laos or lao pdr or "lao people's democratic republic" or latvia or lebanon or lebanese republic or lesotho or basutoland or liberia or libya or libyan arab jamahiriya or lithuania or macau or macao or "macedonia (republic)" or macedonia or madagascar or malagasy republic or malawi or nyasaland or malaysia or malay federation or malaya federation or maldives or indian ocean islands or indian ocean or mali or malta or micronesia or federated states of micronesia or kiribati or marshall islands or nauru or northern mariana islands or palau or tuvalu or mauritania or mauritius or mexico or moldova or moldovian or mongolia or montenegro or "montenegro (republic)" or morocco or ifni or mozambique or portuguese east africa or myanmar or burma or namibia or nepal or netherlands antilles or nicaragua or niger or nigeria or oman or muscat or pakistan or panama or papua new guinea or new guinea or paraguay or peru or philippines or philipines or phillipines or phillippines or poland or "polish people's republic" or portugal or portuguese republic or puerto rico or romania or russia or russian federation or ussr or soviet union or union of soviet socialist republics or rwanda or ruanda or samoa or pacific islands or polynesia or samoan islands or navigator island or navigator islands or "sao tome and principe" or saudi arabia or senegal or serbia or seychelles or sierra leone or slovakia or slovak republic or slovenia or melanesia or solomon island or solomon islands or norfolk island or norfolk islands or somalia or south africa or south sudan or sri lanka or ceylon or "saint kitts and nevis" or "st. kitts and nevis" or saint lucia or "st. lucia" or "saint vincent and the grenadines" or saint vincent or "st. vincent" or grenadines or sudan or suriname or surinam or dutch guiana or netherlands guiana or syria or syrian arab republic or tajikistan or tadjikistan or tadzhikistan or tadzhik or tanzania or tanganyika or thailand or siam or timor leste or east timor or togo or togolese republic or tonga or "trinidad and tobago" or trinidad or tobago or tunisia or turkey or "turkey (republic)" or turkmenistan or turkmen or uganda or ukraine or uruguay or uzbekistan or uzbek or vanuatu or new hebrides or venezuela or vietnam or viet nam or middle east or west bank or gaza or palestine or yemen or yugoslavia or zambia or zimbabwe or northern rhodesia or global south or africa south of the sahara or "sub saharan africa" or subsaharan africa or africa, central or central africa or africa, northern or north africa or northern africa or magreb or maghrib or sahara or africa, southern or southern africa or africa, eastern or east africa or eastern africa or africa, western or west africa or western africa or west indies or indian ocean islands or caribbean region or caribbean islands or caribbean or central america or latin america or "south and central america" or south america or asia, central or central asia or asia, northern or north asia or northern asia or asia, southeastern or southeastern asia or south eastern asia or southeast asia or south east asia or asia, western or western asia or europe, eastern or east europe or eastern europe or developing country or developing countries or developing nation? or developing population? or developing world or less developed countr* or less developed nation? or less developed population? or less developed world or lesser developed countr* or lesser developed nation? or lesser developed population? or lesser developed world or under developed countr* or under developed nation? or under developed population? or under developed world or underdeveloped countr* or underdeveloped nation? or underdeveloped population? or underdeveloped world or middle income countr* or middle income nation? or middle income population? or low income countr* or low income nation? or low income population? or lower income countr* or lower income nation? or lower income population? or underserved countr* or underserved nation? or underserved population? or underserved world or under served countr* or under served nation? or under served population? or under served world or deprived countr* or deprived nation? or deprived population? or deprived world or poor countr* or poor nation? or poor population? or poor world or poorer countr* or poorer nation? or poorer population? or poorer world or developing econom* or less developed econom* or lesser developed econom* or under developed econom* or underdeveloped econom* or middle income econom* or low income econom* or lower income econom* or low gdp or low gnp or low gross domestic or low gross national or lower gdp or lower gnp or lower gross domestic or lower gross national or lmic or lmics or third world or lami countr* or transitional countr* or emerging economies or emerging nation?).mp. [mp=title, abstract, heading word, table of contents, key concepts, original title, tests & measures, mesh] (270847)

26 1 and 2 and 11 (2437)

27 1 and 2 and 11 and 24 and 25 (580)

Database: Embase <1974 to 2020 August 25>

Search results:

194 returned - 37 duplicates removed = 157 unique records

Search Strategy:

1 (Men or man or male or males or boy or boys or masculin* or father* or husband).ti,ab,sh,kw,hw. (9594063)

2 (Family planning or ((unintended or unwanted or unplanned or planned or wanted or intended) adj Pregnan*) or Contracepti* or birth control or adolescent pregnancy or birth spacing or birth interval* or child spacing or pregnancy interval* or delay pregnancy or abortion or abortions).ti,ab,sh,kw,hw. (257491)

3 (afghanistan or albania or algeria or american samoa or angola or "antigua and barbuda" or antigua or barbuda or argentina or armenia or armenian or aruba or azerbaijan or bahrain or bangladesh or barbados or republic of belarus or belarus or byelarus or belorussia or byelorussian or belize or british honduras or benin or dahomey or bhutan or bolivia or "bosnia and herzegovina" or bosnia or herzegovina or botswana or bechuanaland or brazil or brasil or bulgaria or burkina faso or burkina fasso or upper volta or burundi or urundi or cabo verde or cape verde or cambodia or kampuchea or khmer republic or cameroon or cameron or cameroun or central african republic or ubangi shari or chad or chile or china or colombia or comoros or comoro islands or iles comores or mayotte or democratic republic of the congo or democratic republic congo or congo or zaire or costa rica or "cote d’ivoire" or "cote d’ ivoire" or cote divoire or cote d ivoire or ivory coast or croatia or cuba or cyprus or czech republic or czechoslovakia or djibouti or french somaliland or dominica or dominican republic or ecuador or egypt or united arab republic or el salvador or equatorial guinea or spanish guinea or eritrea or estonia or eswatini or swaziland or ethiopia or fiji or gabon or gabonese republic or gambia or "georgia (republic)" or georgian or ghana or gold coast or gibraltar or greece or grenada or guam or guatemala or guinea or guinea bissau or guyana or british guiana or haiti or hispaniola or honduras or hungary or india or indonesia or timor or iran or iraq or isle of man or jamaica or jordan or kazakhstan or kazakh or kenya or "democratic people’s republic of korea" or republic of korea or north korea or south korea or korea or kosovo or kyrgyzstan or kirghizia or kirgizstan or kyrgyz republic or kirghiz or laos or lao pdr or "lao people's democratic republic" or latvia or lebanon or lebanese republic or lesotho or basutoland or liberia or libya or libyan arab jamahiriya or lithuania or macau or macao or "macedonia (republic)" or macedonia or madagascar or malagasy republic or malawi or nyasaland or malaysia or malay federation or malaya federation or maldives or indian ocean islands or indian ocean or mali or malta or micronesia or federated states of micronesia or kiribati or marshall islands or nauru or northern mariana islands or palau or tuvalu or mauritania or mauritius or mexico or moldova or moldovian or mongolia or montenegro or "montenegro (republic)" or morocco or ifni or mozambique or portuguese east africa or myanmar or burma or namibia or nepal or netherlands antilles or nicaragua or niger or nigeria or oman or muscat or pakistan or panama or papua new guinea or new guinea or paraguay or peru or philippines or philipines or phillipines or phillippines or poland or "polish people's republic" or portugal or portuguese republic or puerto rico or romania or russia or russian federation or ussr or soviet union or union of soviet socialist republics or rwanda or ruanda or samoa or pacific islands or polynesia or samoan islands or navigator island or navigator islands or "sao tome and principe" or saudi arabia or senegal or serbia or seychelles or sierra leone or slovakia or slovak republic or slovenia or melanesia or solomon island or solomon islands or norfolk island or norfolk islands or somalia or south africa or south sudan or sri lanka or ceylon or "saint kitts and nevis" or "st. kitts and nevis" or saint lucia or "st. lucia" or "saint vincent and the grenadines" or saint vincent or "st. vincent" or grenadines or sudan or suriname or surinam or dutch guiana or netherlands guiana or syria or syrian arab republic or tajikistan or tadjikistan or tadzhikistan or tadzhik or tanzania or tanganyika or thailand or siam or timor leste or east timor or togo or togolese republic or tonga or "trinidad and tobago" or trinidad or tobago or tunisia or turkey or "turkey (republic)" or turkmenistan or turkmen or uganda or ukraine or uruguay or uzbekistan or uzbek or vanuatu or new hebrides or venezuela or vietnam or viet nam or middle east or west bank or gaza or palestine or yemen or yugoslavia or zambia or zimbabwe or northern rhodesia or global south or africa south of the sahara or "sub saharan africa" or subsaharan africa or africa, central or central africa or africa, northern or north africa or northern africa or magreb or maghrib or sahara or africa, southern or southern africa or africa, eastern or east africa or eastern africa or africa, western or west africa or western africa or west indies or indian ocean islands or caribbean region or caribbean islands or caribbean or central america or latin america or "south and central america" or south america or asia, central or central asia or asia, northern or north asia or northern asia or asia, southeastern or southeastern asia or south eastern asia or southeast asia or south east asia or asia, western or western asia or europe, eastern or east europe or eastern europe or developing country or developing countries or developing nation? or developing population? or developing world or less developed countr* or less developed nation? or less developed population? or less developed world or lesser developed countr* or lesser developed nation? or lesser developed population? or lesser developed world or under developed countr* or under developed nation? or under developed population? or under developed world or underdeveloped countr* or underdeveloped nation? or underdeveloped population? or underdeveloped world or middle income countr* or middle income nation? or middle income population? or low income countr* or low income nation? or low income population? or lower income countr* or lower income nation? or lower income population? or underserved countr* or underserved nation? or underserved population? or underserved world or under served countr* or under served nation? or under served population? or under served world or deprived countr* or deprived nation? or deprived population? or deprived world or poor countr* or poor nation? or poor population? or poor world or poorer countr* or poorer nation? or poorer population? or poorer world or developing econom* or less developed econom* or lesser developed econom* or under developed econom* or underdeveloped econom* or middle income econom* or low income econom* or lower income econom* or low gdp or low gnp or low gross domestic or low gross national or lower gdp or lower gnp or lower gross domestic or lower gross national or lmic or lmics or third world or lami countr* or transitional countr* or emerging economies or emerging nation?).ti,ab,sh,kw. (2181173)

4 1 and 2 and 3 (11202)

5 randomized controlled trial/ or controlled clinical trial/ or pragmatic clinical trial/ or multicenter study/ (956527)

6 non-randomized controlled trials as topic/ (10979)

7 interrupted time series analysis/ (192885)

8 controlled before-after studies/ (198241)

9 (randomis* or randomiz* or randomly).ti,ab. (1282390)

10 groups.ab. (2901366)

11 (trial or multicenter or multi center or multicentre or multi centre).ti. (374161)

12 (intervention? or effect? or impact? or controlled or control group? or (before adj5 after) or (pre adj5 post) or ((pretest or pre test) and (posttest or post test)) or quasiexperiment* or quasi experiment* or evaluat* or time series or time point? or repeated measur* or ((nonequivalent or non equivalent) adj3 control*)).ti,ab. (12488085)

13 (program* and evaluat*).ti,ab,kw. (294440)

14 5 or 6 or 7 or 8 or 9 or 10 or 11 or 12 or 13 (14043122)

15 exp Animals/ (25932092)

16 Humans/ (13718888)

17 15 not 16 (12215392)

18 14 not 17 (7799224)

19 4 and 18 (3343)

20 educat*.mp. (1325311)

21 behav*.mp. (2033145)

22 (peer or community).mp. [mp=title, abstract, heading word, drug trade name, original title, device manufacturer, drug manufacturer, device trade name, keyword, floating subheading word, candidate term word] (797071)

23 (psycho* or social*).mp. [mp=title, abstract, heading word, drug trade name, original title, device manufacturer, drug manufacturer, device trade name, keyword, floating subheading word, candidate term word] (2300677)

24 (Counselling or counseling).mp. [mp=title, abstract, heading word, drug trade name, original title, device manufacturer, drug manufacturer, device trade name, keyword, floating subheading word, candidate term word] (204435)

25 (media or communication or marketing).mp. [mp=title, abstract, heading word, drug trade name, original title, device manufacturer, drug manufacturer, device trade name, keyword, floating subheading word, candidate term word] (1117138)

26 (provision or provide or provided).mp. [mp=title, abstract, heading word, drug trade name, original title, device manufacturer, drug manufacturer, device trade name, keyword, floating subheading word, candidate term word] (2577759)

27 (delivery or delivered or deliver).mp. [mp=title, abstract, heading word, drug trade name, original title, device manufacturer, drug manufacturer, device trade name, keyword, floating subheading word, candidate term word] (1058550)

28 (distribution or distributed or distribute).mp. [mp=title, abstract, heading word, drug trade name, original title, device manufacturer, drug manufacturer, device trade name, keyword, floating subheading word, candidate term word] (1681376)

29 (train or training).mp. [mp=title, abstract, heading word, drug trade name, original title, device manufacturer, drug manufacturer, device trade name, keyword, floating subheading word, candidate term word] (660980)

30 mhealth.mp. (4483)

31 (program* or intervention*).mp. [mp=title, abstract, heading word, drug trade name, original title, device manufacturer, drug manufacturer, device trade name, keyword, floating subheading word, candidate term word] (2725444)

32 20 or 21 or 22 or 23 or 24 or 25 or 26 or 27 or 28 or 29 or 30 or 31 (11166754)

33 4 and 18 and 32 (2789)

34 limit 33 to exclude medline journals (194)

### Appendix 3.0: Data Extraction Form

| Study Characteristics |
| --- |
| - - Author Primary Author Name |
| - - Author Contact Contact details for study first (or corresponding) author. |
| - - Publication Year |
| - - Article Title |
| - - Publication Type Type of record publication, e.g. academic journal, grey literature report, thesis |
| - - Funder |
| - - Study Design |
| - - Study/Intervention Aim The stated aim of the intervention or study. |
| - - Country Country(ies) of implementation |
| - - Sample Size (Intervention Group) |
| - - Sample Size (Control Group) |
| - - Sample Characteristics  Description of the study sample, e.g. adolescents, adults, married. |
| - - Stage of Programme Stage of programme in reporting, e.g. Pilot, Scale-up, Evaluation, Transfer, Adaptation. |
| **Intervention Characteristics** Intervention characteristics as per the TIDiER guidelines. Check-off any information present and include details provide by authors. |
| - - Details of Intervention Inputs Details of Programme Inputs Provided (e.g. Time and Cost; Implementers; Intervention Materials; Participant and Volunteer Incentives) |
| - - Intervention Components What categories do the intervention components address (select all that apply). |
| - - - Gender  Addressing unequal inequalities and harmful/restrictive gender norms |
| - - - Information e.g. Information provision in clinics, educational settings, community settings, comprehensive sex education |
| - - - Skills e.g. workshops, demonstrations, behaviour modelling |
| - - - Problem Solving Identifying barriers and facilitators of FP communication and access; decision making |
| - - - Social Support e.g. outreach with male motivators, mentors, peer support, engaging religious leaders, community dialogue |
| - - - Incentives e.g. conditional cash transfer, vouchers for FP services, free contraceptives |
| - - - Communication e.g. social marketing, mass media, social media, MHealth, hotlines |
| - - - Health Service Enhancement e.g. low cost/free access to FP methods services; health service adaptation |
| - - - Male Involvement e.g. explicit tailoring messages to engage & involve men |
| - - Intervention Name Stated name of the interventions programme(s) where available. |
| - - Goal/Rationale Describe primary rationale or intended goal of the central elements to the intervention (e.g. Increased knowledge and use of contraceptives). |
| - - Theories Applied List/Describe any Theories of Behaviour change underpinning the intervention. |
| - - Intervention Design The design of the intervention as described by the study authors. (e.g. Comprehensive Sex Education, Skills Building Workshops) |
| - - Comparison Characteristics What condition the Control or Comparison group(s) received |
| - - - No Intervention Control group allocated to a waiting list, received no intervention, or received standard treatment available to both arms (e.g. national sexual education curriculum) |
| - - - Attention Control Comparison group received an alternative intervention unrelated to FP. |
| - - - Alternative Intervention Comparison group received another intervention related to Family Planning. |
| - - Materials and Procedure Describe any activities used in the interventions. Description of any physical or informational materials used in the intervention, including those provided to participants or used in intervention delivery or in training of intervention providers. |
| - - Who Provided Note each category of intervention provider (e.g. psychologist, nursing assistant). Describe any expertise, background and specific training given. |
| - - Who Received  Brief Description of participants who took part in the intervention. Record characteristics according to PROGRESS Plus criteria using child codes. |
| - - - Place Place of Residence (e.g. Rural/urban, country/state, housing characteristics) |
| - - - Race Ethnic background |
| - - - Occupation Professional, skilled, unskilled, unemployed etc. |
| - - - Gender Male, Female, Both |
| - - - Religion Religious background |
| - - - Education Years in and/or level of education attained, school type |
| - - - Social Capital Neighbourhood / community / family support |
| - - - Socio-Economic Status Income, means tested benefits/welfare, affluence measures, etc. |
| - - - Age Age range |
| - - - Disability Existence of physical or emotional/mental disability |
| - - - Sexual Orientation Heterosexual, gay, lesbian, bisexual, transgender |
| - - - Other Vulnerable Group e.g. School non-attenders, looked after young people, young people in criminal justice system, victims of abuse, runaways, teenage parents. |
| - - Mode of Delivery Describe the modes of delivery (e.g. face-to-face or by some other mechanism, such as internet or telephone) of the intervention and whether it was provided individually or in a group. |
| - - Delivery Setting Describe the type(s) of location(s) where the intervention occurred, including any necessary infrastructure or relevant features mentioned (e.g. Schools, Health Centres). |
| - - Delivery Dosage Note the number of times the intervention was delivered and over what period of time including the number of sessions, their schedule, and their duration, intensity or dose.  Allocate to dosage category.     - Less than 3 months     - 3-6 months     - 7-12 months     - Over 12 months |
| - - Details of Tailoring Describe any planned iteration or adaptation in delivery. Note any details provided of tailoring (what, why, when, and how). |
| - - Details of Modification If the intervention was modified during the course of the study, describe the changes (what, why, when, and how). |
| - - Details of Fidelity Assessment Was the intervention delivered as planned? If intervention adherence or fidelity was assessed, describe how and by whom, and if any strategies were used to maintain or improve fidelity, describe them. |
| **Moderators** Record any potential moderators of intervention effect considered by the study. |
| - - Individual Level Moderators (e.g. Age/ life stage; Sex; Ethnicity; Disability; Sexual orientation; Gender identity; Religion/religiosity; HIV/AIDS/STI status; Marital status /history/type; Relationship status/quality;Reproductive history; Sex of existing children; Past FP behaviours & experiences; Co residence with children, partner, extended family; Urban/rural residence; Migrant status; Socioeconomic factors; Attitudes values and beliefs about FP; Perceived gender and cultural norms; Attitudes about sexual pleasure) |
| - - External Moderators (e.g. Gender Norms; Cultural Norms; Religious Norms; Political and Economic Climate; Legal Context; Current Health Policy and Strategies; Health Systems and Services; FP Supply Chain; Conflict; Disaster; Disease Outbreak; Climate-Stressed Environment; OTHER) |
| - - Process Metrics (e.g. Intervention Acceptability; Intervention Cost; Intervention Sustainability; Quality of Delivery; Provider Preparedness; Participant Recruitment and Retention; Study Design and Characteristics) |
| **Outcomes** List any outcome(s) described by the study under the relevant categories. |
| - - Measure(s) Used Details of any measures or scales used to capture outcomes (e.g. Self-report, Interviewer Administered, Scale names). |
| - - Mixed Methods Used Note if this evaluation/study also included qualitative analysis. |
| - - Individual Level Outcomes (Gender Equitable Attitudes; Knowledge related to FP; Attitudes related to FP; Skills related to FP; Contraceptive Uptake and Use; Support for Female Partner Contraceptive Uptake and Use; Delayed Pregnancy; Birth Spacing and Limiting; Support for Male Involvement in FP, Other) |
| - - - Gender Equitable Beliefs More gender equitable beliefs around relationships. |
| - - - Sexual Behaviours Participation in safe sexual behaviours, Abstinence. |
| - - - Sexual Behaviour Attitudes Views on sexuality, Norms around sexual practices and abstinence. |
| - - - Reproductive Health Knowledge Understanding of reproductive biology and health issues (e.g. pregnancy). |
| - - - Contraceptive Knowledge Knowledge related to contraceptives and their use. |
| - - - Contraceptive Use Behaviours Contraceptive uptake and (continued) use. |
| - - - Contraceptive Attitudes & Support Attitudes/support for contraceptive use and family planning services. Male support for partner's contraceptive use. |
| - - - Birth Spacing Spacing, limiting, or delay of births and pregnancies. |
| - - - Abortion Use of safe abortion or abortive care. |
| - - - FP Service Attitudes Improved trust, perceptions, or satisfaction with Family Planning Services. |
| - - - Service Use Increased help-seeking or Family Planning service use |
| - - Interpersonal Level Outcomes (Communication and Quality of Relationships; Equitable Relationships and FP Decision-making, Other) |
| - - - Communication Increased communication about Family Planning. |
| - - - External Support Support from community members (e.g. family members, opinion leaders, peers) for Family Planning. |
| - - - Equitable Relationships More equitable romantic relationships. |
| - - - Joint FP Decision Making Joint decision making between men and women on family planning use. |
| - - Service/Organisation Level Outcomes (Service Provider and Educator’s Gender Equitable Attitudes; Provision and Polices Involving Males; Quality and Accessibility of FP Services for Males, Other) |
| - - - Gender Equitable Attitudes Increased gender equitable attitudes among family planning service providers/educators. |
| - - - Policies Policies that prioritise male involvement in Family Planning. |
| - - - Provider Engagement Increased provider engagement of males. |
| - - - Service Accessibility Increased quality or accessibility of services for males. |
| - - Structural Level Outcomes (Policy Support for Gender Equality; Policy Support for Male Involvement in FP; Resources for FP Provision, Other) |
| - - - Gender Equality Increased policy support for gender equality. |
| - - - Male Involvement Increased policy support for male involvement in Family Planning. |
| - - - Resources Increased resource provision for Family Planning. |
| - Impacts Note any Distal Outcomes or Impacts of intervention described. |
| Results |
| - - Overall Results Summary Summarise or copy the overall conclusion of the study. Note whether the overall results were Positive, Negative, or Mixed using child codes. |
| - - - Positive Overall intervention effect was Positive on intended outcomes. |
| - - - Mixed Overall intervention effect was Mixed on intended outcomes. |
| - - - Negative Overall intervention effect was Negative on intended outcomes. |

### Appendix 4.0: Characteristics of Included Studies

### Appendix 4.1 Characteristics Evaluation Studies

| Authors, Year | Study Title | Study Design | Recipients | Country | Result  Positive: Effects in intended direction  Negative: No effects  Mixed: Some effects in intended direction for some outcomes and no effects for some outcomes |
| --- | --- | --- | --- | --- | --- |
| (Acharya, 2014) | Measuring the effectiveness of teachings sex education in Nepalese secondary schools: An outcome from a Randomised Controlled Trial (RCT) | RCT | Male and Female Adolescents, School Students | Nepal | Positive |
| (Agha, 2002) | A quasi-experimental study to assess the impact of four adolescent sexual health interventions in sub-Saharan Africa | QE | Male and Female Adolescents and their Parents | Cameroon, South Africa, Botswana, Guinea | Positive |
| (Agha et al., 2007) | The impact of a reproductive health franchise on client satisfaction in rural Nepal | QE | Male and Female Adults, Healthcare Clients | Nepal | Mixed |
| (Ahmed et al., 2015) | The Effect of Integrating Family Planning with a Maternal and Newborn Health Program on Postpartum Contraceptive Use and Optimal Birth Spacing in Rural Bangladesh | QE | Pregnant and Post-partum Women and Their Male Partners | Bangladesh | Positive |
| (Ajuwon & Brieger, 2007) | Evaluation of a school-based reproductive health education program in rural South Western, Nigeria | QE | Male and Female Adolescents & Young People, School Students | Nigeria | Positive |
| (Almeida et al., 2019) | Effects of an Educational Intervention on Angolan Adolescents' Knowledge of Human Reproduction: A Quasi-Experimental Study | QE | Male and Female Adolescents, School Students | Angola | Positive |
| (Amatya et al., 1994) | The effect of husband counseling on NORPLANT contraceptive acceptability in Bangladesh | QE | Male Husbands of Female NORPLANT Trial Participants | Bangladesh | Mixed |
| (Amin & Suran, 2005) | Program Efforts to Delay Marriage Through Improved Opportunities: Some Evidence from Rural Bangladesh | QE | Male and Female Adolescents and Community Opinion Leaders | Bangladesh | Mixed |
| (Andrade et al., 2009) | Changes in sexual behavior following a sex education program in Brazilian public schools | QE | Male and Female Adolescents, School Students. Students' families, and the Local Community within the school environment | Brazil | Mixed |
| (Aninanya et al., 2015) | Effects of an adolescent sexual and reproductive health intervention on health service usage by young people in northern Ghana: a community-randomised trial | RCT | Male and Female Adolescents, Community Leaders (Chiefs and Religious Leaders) | Ghana | Mixed |
| (Atwood et al., 2012) | Reducing sexual risk taking behaviors among adolescents who engage in transactional sex in post-conflict Liberia | RCT | Male and Female Adolescents | Liberia | Mixed |
| (Bachanas et al., 2016) | Delivering Prevention Interventions to People Living with HIV in Clinical Care Settings: Results of a Cluster Randomized Trial in Kenya, Namibia, and Tanzania | cRCT | Male and Female Adults, Living with HIV | Kenya, Namibia, Tanzania | Mixed |
| (Bertrand et al., 1987) | Evaluation of a communication program to increase adoption of vasectomy in Guatemala | QE | Male Adolescents and Adults, targeted by Communications | Guatemala | Mixed |
| (Black & Harvey, 1976) | A report on a contraceptive social marketing experiment in rural Kenya | QE | Male and Female Adolescents and Adults | Kenya | Positive |
| (Boti et al., 2019) | Effects of comprehensive sexuality education on the comprehensive knowledge and attitude to condom use among first-year students in Arba Minch University: a quasi-experimental study | QE | Male and Female Adults, University Students | Ethiopia | Positive |
| (Brieger et al., 2001) | West African Youth Initiative: outcome of a reproductive health education program | QE | Male and Female Adolescents and Young People | Nigeria, Ghana | Mixed |
| (Caceres et al., 1994) | Evaluating a school-based intervention for STD/AIDS prevention in Peru | RCT | Male and Female Adolescents and Young People, School Students | Peru | Positive |
| (Campero et al., 2011) | A quasi-experimental evaluation of parents as sexual health educators resulting in delayed sexual initiation and increased access to condoms | QE | Male and Female Adults, Parents of Adolescents | Mexico | Positive |
| (Campero et al., 2019) | Intervention for the comprehension of menstrual cycle, body function and pregnancy in adolescents from rural context | QE | Male and Female Adolescents | Mexico | Positive |
| (Chi et al., 2015) | The effect of comprehensive sexual education program on sexual health knowledge and sexual attitude among college students in Southwest China | QE | Male and Female Adults, University Students | China | Mixed |
| (Chong et al., 2013) | Effectiveness and Spillovers of Online Sex Education: Evidence from a Randomized Evaluationin Colombian Public Schools | RCT | Male and Female Adolescents | Colombia | Positive |
| (Coffman et al., 2011) | Effects of HealthWise South Africa on condom use self-efficacy | RCT | Male and Female Adolescents | South Africa | Positive |
| (Conboy et al., 2003) | A cluster randomized trial of a sex education programmed in Belize, Central America | cRCT | Male and Female Adolescents | Belize | Mixed |
| (Cowan et al., 2010) | The Regai Dzive Shiri project: results of a randomized trial of an HIV prevention intervention for youth | RCT | Male and Female Adolescents & Young People, Parents, Health Clinic Staff | Zimbabwe | Mixed |
| (Daniel & Nanda, 2012) | The Effect of Reproductive Health Communication Interventions on Age at Marriage and First Birth in Rural Bihar, India: A retrospective study | QE | Male and Female Adolescents | India | Positive |
| (M. A. Daniele et al., 2018) | Involving male partners in maternity care in Burkina Faso: a randomized controlled trial | RCT | Pregnant Women (15-45) and their Male Partners | Burkina Faso | Positive |
| (Debpuur et al., 2002) | The impact of the Navrongo Project on contraceptive knowledge and use, reproductive preferences, and fertility | QE | Males and Females of Reproductive Age | Ghana | Mixed |
| (Decat et al., 2014) | Community-based interventions promoting adolescents' sexual health in three Latin-American cities: impact and impact modifying factors | RCT | Male and Female Adolescents | Bolivia, Ecuador, Nicaragua | Mixed |
| (Decat, 2015) | Addressing the unmet contraceptive need of adolescents and unmarried youth: Act or Interact Learning from comprehensive interventions in China and Latin America | QE | Male and Female Adolescents, their Parents/Adult Family Members, Healthcare Professionals | Bolivia, Ecuador, Nicaragua | Mixed |
| (Diop et al., 2004) | Improving the reproductive health of adolescents in Senegal | QE | Male and Female Adolescents | Senegal | Mixed |
| (Doyle et al., 2011) | The long-term impact of the MEMA kwa Vijana adolescent sexual and reproductive health intervention: effect of dose and time since intervention exposure | RCT | Male and Female Adolescents and Young Adults | Tanzania | Mixed |
| (Doyle et al., 2018) | Gender-transformative Bandebereho couples' intervention to promote male engagement in reproductive and maternal health and violence prevention in Rwanda: Findings from a randomized controlled trial | RCT | Male and Female Adults, Couples | Rwanda | Positive |
| (Eggleston et al., 2000) | Evaluation of a sexuality education program for young adolescents in Jamaica | QE | Male and Female Adolescents School Students | Jamaica | Negative |
| (El‐Khoury et al., 2016) | Counseling Women and Couples on Family Planning: A Randomized Study in Jordan | RCT | Male and Female Adults, Married and Co-Habiting Couples | Jordan | Positive |
| (Erulkar et al., 2004) | Behavior change evaluation of a culturally consistent reproductive health program for young Kenyans | QE | Male and Female Adolescents and Young People, and Opinion Leaders | Kenya | Mixed |
| (Exner et al., 2009) | Mobilizing men as partners: the results of an intervention to increase dual protection among Nigerian men | QE | Male Adults | Nigeria | Positive |
| (Firestone et al., 2016) | Intensive Group Learning and On-Site Services to Improve Sexual and Reproductive Health Among Young Adults in Liberia: A Randomized Evaluation of HealthyActions | RCT | Male and Female Adolescents and Young People | Liberia | Positive |
| (Fisek & Sumbuloglu, 1978) | The effects of husband and wife education on family planning in rural Turkey | QE | Males of Reproductive Age, Married Males and Females | Turkey | Mixed |
| (Fitzgerald et al., 1999) | Use of Western-based HIV risk-reduction interventions targeting adolescents in an African setting | RCT | Male and Female Adolescents | Namibia | Mixed |
| (Fleming et al., 2018) | Can a Gender Equity and Family Planning Intervention for Men Change Their Gender Ideology? Results from the CHARM Intervention in Rural India | RCT | Male and Female Adults, Married Couples | India | Mixed |
| (Foreit et al., 1992) | A comparison of the performance of male and female CBD distributors in Peru | QE | Males and Females of Reproductive Age | Peru | Positive |
| (Gallegos et al., 2008) | Intervention to reduce adolescents sexual risk behaviors: a randomized controlled trial | RCT | Male and Female Adolescents | Mexico | Positive |
| (Gao et al., 2001) | AIDS and sex education for young people in China | RCT | Male and Female Adolescents and Young People, University Students | China | Mixed |
| (Ha et al., 2005b) | Increasing male involvement in family planning decision making: trial of a social-cognitive intervention in rural Vietnam | QE | Male Adults, Married | Vietnam | Positive |
| (Hallman & Roca, 2011) | Siyakha Nentsha: Building economic, health, and social capabilities among highly vulnerable adolescents in KwaZulu-Natal, South Africa | QE | Male and Female Adolescents, School Students | South Africa | Positive |
| (Halpern et al., 2008) | Effectiveness of web-based education on Kenyan and Brazilian adolescents' knowledge about HIV/AIDS, abortion law, and emergency contraception: Findings from TeenWeb | QE | Male and Female Adolescents, School Students | Kenya, Brazil | Mixed |
| (Harrington, 2017b) | Evaluation of an mHealth SMS Dialogue Strategy to Meet Women's and Couples' Postpartum Contraceptive Needs in Kenya (Mobile WACh XY): a Randomized Controlled Trial | RCT | Pregnant Females and their Male Partners | Kenya | Mixed |
| (Harrington et al., 2019) | An mHealth SMS intervention on Postpartum Contraceptive Use Among Women and Couples in Kenya: A Randomized Controlled Trial | RCT | Pregnant Females and their Male Partners | Kenya | Mixed |
| (He et al., 2012) | Promoting Contraceptive Use More Effectively Among Unmarried Male Migrants in Construction Sites in China: A Pilot Intervention Trial | QE | Male Adolescents and Young Adults, Migrant Workers | China | Positive |
| (Jewkes et al., 2008) | Impact of stepping stones on incidence of HIV and HSV-2 and sexual behaviour in rural South Africa: cluster randomised controlled trial | cRCT | Male and Female Adolescents and Young Adults | South Africa | Negative |
| (Johnson et al., 2017) | A randomized controlled trial of the impact of a family planning mHealth service on knowledge and use of contraception | RCT | Male and Female Adults | Kenya | Mixed |
| (Kaljee et al., 2012) | A sexual health program for Vietnamese parents of adolescents: A preliminary report on changes in parental knowledge, communication, and self-efficacy for condom use | RCT | Male and Female Adolescents, and their Parents | Vietnam | Positive |
| (Kanesathasan et al., 2008) | Catalyzing change: Improving youth sexual and reproductive health through DISHA, an integrated program in India | QE | Male and Female Adolescents and Adults | India | Mixed |
| (Katz et al., 1998) | Increasing access to family planning services in rural Mali through community-based distribution | QE | Male and Female Adolescents and Adults | Mali | Positive |
| (Kemigisha et al., 2019) | Evaluation of a school based comprehensive sexuality education program among very young adolescents in rural Uganda | RCT | Male and Female Adolescents, School Students | Uganda | Mixed |
| (Khanm & Huber, 1976) | Door-to-door distribution working in Bangladesh | QE | Married Females and their Husbands | Bangladesh | Positive |
| (Khatun et al., 2011) | Assessing public-private reproductive health efforts to reach young married couples in rural Bangladesh | QE | Male and Female Adolescents and Young Adults, Married Couples | Bangladesh | Mixed |
| (Kim et al., 2001) | Promoting sexual responsibility among young people in Zimbabwe | QE | Male and Female Adolescents and Young Adults | Zimbabwe | Positive |
| Kulathinal (2019) | Mobile Helpline and Reversible Contraception: lessons From a Controlled Before-and-After Study in Rural India | QE | Males and Females Adolescents and Young Adults, Married People | India | Mixed |
| (Lemani et al., 2017) | Contraceptive uptake after training community health workers in couples counseling: a cluster randomized trial | cRCT | Female and Male Adults | Malawi | Negative |
| (C. Lou et al., 2004) | Effects of a community-based sex education and reproductive health service program on contraceptive use of unmarried youths in Shanghai | QE | Male and Female Adolescents and Young Adults | China | Positive |
| (Lou et al., 2006) | Can the Internet be used effectively to provide sex education to young people in China? | QE | Male and Female Adolescents and Young Adults | China | Mixed |
| (Luck et al., 2000) | Mobilizing demand for contraception in rural Gambia | QE | Males and Females of Reproductive Age | Gambia | Positive |
| (Lutalo et al., 2010) | A randomized community trial of enhanced family planning outreach in Rakai, Uganda | RCT | Males and Females of Reproductive Age | Uganda | Mixed |
| (Magnani et al., 2001) | Impact of an integrated adolescent reproductive health program in Brazil | QE | Male and Female Adolescents | Brazil | Negative |
| (Mantell et al., 2006) | The Mpondombili Project: preventing HIV/AIDS and unintended pregnancy among rural South African school-going adolescents | QE | Male and Female Adolescents, School Students | South Africa | Mixed |
| (Mantell et al., 2017) | Emtonjeni-A Structural Intervention to Integrate Sexual and Reproductive Health into Public Sector HIV Care in Cape Town, South Africa: Results of a Phase II Study | RCT | Male and Female Adults, Living with HIV | South Africa | Positive |
| (Mark et al., 2007) | Contraception among HIV concordant and discordant couples in Zambia: a randomized controlled trial | RCT | Male and Female Adults, Living with HIV | Zambia | Mixed |
| (Martiniuk et al., 2003) | A cluster randomized trial of a sex education programme in Belize, Central America | cRCT | Male and Female Adolescents, School Students | Belize | Mixed |
| (Mathur et al., 2004) | Youth reproductive health in Nepal: is participation the answer? | QE | Male and Female Adolescents, and Adults | Nepal | Mixed |
| (Mba et al., 2007) | The impact of health education on reproductive health knowledge among adolescents in a rural Nigerian community | QE | Male and Female Adolescents, School Students | Nigeria | Positive |
| (Mbizvo et al., 1997) | Effects of a randomized health education intervention on aspects of reproductive health knowledge and reported behaviour among adolescents in Zimbabwe | RCT | Male and Female Adolescents, School Students | Zimbabwe | Positive |
| (Mbonye, 2003) | Disease and health seeking patterns among adolescents in Uganda | QE | Male and Female Adolescents | Uganda | Positive |
| (McCarthy et al., 2018) | A randomized controlled trial of an intervention delivered by mobile phone app instant messaging to increase the acceptability of effective contraception among young people in Tajikistan | RCT | Male and Female Adolescents and Young Adults | Tajikistan | Negative |
| (Medina et al., 1980) | Comparative evaluation of two methods of natural family planning in Columbia | RCT | Male and Female Adults, Couples | Colombia | Negative |
| (Ngure et al., 2009) | Successful increase in contraceptive uptake among Kenyan HIV-1-serodiscordant couples enrolled in an HIV-1 prevention trial | QE | Male and Female Adults, HIV serodiscordant couples | Kenya | Positive |
| (Odeyemi & Ibude, 2011) | Promoting Male Participation in Family Planning in Rural Nigeria: A Community-based Intervention | QE | Male Adults, Married | Nigeria | Positive |
| (Ojengbede et al., 2009) | Psycho-social support in labour, as a catalyst for contraceptive uptake in Nigeria: preliminary analysis of a randomised controlled trial | RCT | Pregnant Women and their Male Partners | Nigeria | Positive |
| (Onono et al., 2015) | Integrating family planning and HIV services in western Kenya: the impact on HIV-infected patients’ knowledge of family planning and male attitudes toward family planning | RCT | Male and Female Adults | Kenya | Mixed |
| (Oyugi et al., 2017) | Accessibility of long-term family planning methods: a comparison study between Output Based Approach (OBA) clients verses non-OBA clients in the voucher supported facilities in Kenya | QE | Males and Females of Reproductive Age | Kenya | Positive |
| (Ozcebe & Akin, 2003) | Effects of Peer Education on Reproductive Health Knowledge for Adolescents Living in Rural Areas of Turkey | QE | Male and Female Adolescents | Turkey | Positive |
| (Ozgur et al., 2000) | The effects of family planning education provided to different gender groups | QE | Males and Females of Reproductive Age | Turkey | Mixed |
| (Pham et al., 2012) | Evaluation of three adolescent sexual health programs in ha noi and khanh hoa province, Vietnam | RCT | Male and Female Adolescents, and their Parents, and Healthcare providers | Vietnam | Mixed |
| (Phillips et al., 2012) | The long-term fertility impact of the Navrongo project in northern Ghana | QE | Male and Female Adolescents and Adults | Ghana | Mixed |
| (Pinchoff et al., 2019) | The evaluation of the Woman's Condom marketing approach: what value did peer-led interpersonal communication add to the promotion of a new female condom in urban Lusaka? | RCT | Community intervention, Targeted Male and Female Adolescents and Young people | Zambia | Mixed |
| (Pineda et al., 1983; Pitt et al., 1999) | Increasing the effectiveness of community workers through training of spouses: a family planning experiment in Guatemala | QE | Male and Female Adults, Contraceptive distributors and their partners | Guatemala | Positive |
| (Pitt et al., 1999) | Credit programs for the poor and reproductive behavior in low-income countries: are the reported causal relationships the result of heterogeneity bias? | QE | Male and Female Adults | Bangladesh | Mixed |
| (Pivatti et al., 2019) | The use of educational strategies for promotion of knowledge, attitudes and contraceptive practices among teenagers - A randomized clinical trial | RCT | Male and Female Adolescents, School Students | Brazil | Positive |
| (Prasertsawat & Koktatong, 2002) | Hands-on is better than look-on: condom use | RCT | Male Adolescents and Young Adults | Thailand | Mixed |
| (Raj et al., 2016) | Cluster Randomized Controlled Trial Evaluation of a Gender Equity and Family Planning Intervention for Married Men and Couples in Rural India | cRCT | Male and Female Adults, Married couples | India | Positive |
| (Rogers et al., 1999) | Effects of an entertainment-education radio soap opera on family planning behavior in Tanzania | QE | Male and Female Adolescents and Adults | Tanzania | Positive |
| (Ross et al., 2007) | Biological and behavioural impact of an adolescent sexual health intervention in Tanzania: a community-randomized trial | RCT | Male & Female Adolescents and Young Adults | Tanzania | Mixed |
| (Rusakaniko et al., 1997) | Trends in reproductive health knowledge following a health education intervention among adolescents in Zimbabwe | RCT | Male and Female Adolescents, School Students | Zimbabwe | Positive |
| (Sahip & Turan, 2007) | Education for expectant fathers in workplaces in Turkey | QE | Male Adults, Expectant Fathers | Turkey | Negative |
| (Schuler, 2012) | Interactive workshops to promote gender equity and family planning in rural communities of Tanzania: Results of a field test | RCT | Male and Female Adolescents and Adults, Couples | Tanzania | Mixed |
| (Schuler et al., 2015) | Interactive Workshops to Promote Gender Equity and Family Planning in Rural Communities of Guatemala: Results of a Community Randomized Study | RCT | Male and Female Adolescents and Adults, Couples | Guatemala | Mixed |
| (Sebastian et al., 2010) | Promoting healthy spacing between pregnancies in India: need for differential education campaigns | QE | Male and Female Adolescents and Adults.  Pregnant Women, Male Partners, and Mothers-in-law | India | Positive |
| (Shattuck et al., 2011) | Encouraging Contraceptive Uptake by Motivating Men to Communicate About Family Planning: The Malawi Male Motivator Project | RCT | Male Adults | Malawi | Mixed |
| (Shuey et al., 1999) | Increased sexual abstinence among in-school adolescents as a result of school health education in Soroti district, Uganda | RCT | Male and Female Adolescents, School Students | Uganda | Positive |
| (Singh et al., 2016) | Supportive supervision for volunteers to deliver reproductive health education: a cluster randomized trial | cRCT | Males and Females of Reproductive Age | Uganda | Positive |
| (Speizer et al., 2001) | An evaluation of the "Entre Nous Jeunes" peer-educator program for adolescents in Cameroon | QE | Male and Female Adolescents and Young Adults | Cameroon | Positive |
| (Stephenson et al., 2011) | A randomized controlled trial to promote long-term contraceptive use among HIV-serodiscordant and concordant positive couples in Zambia | RCT | Male and Female Adults, HIV concordant and discordant couples | Zambia | Positive |
| (Subramanian et al., 2018) | Increasing Contraceptive Use Among Young Married Couples in Bihar, India: Evidence From a Decade of Implementation of the PRACHAR Project | QE | Male and Female Adolescents and Young Adults, Their Family, Community Opinion Leaders, Health Service Providers | India | Mixed |
| (Tang et al., 2009) | Improving reproductive health knowledge in rural China--a web-based strategy | RCT | Male and Female Adolescent and Adults, Healthcare Providers and Educators | China | Positive |
| (Taylor et al., 2014) | Effects of a teenage pregnancy prevention program in KwaZulu-Natal, South Africa | RCT | Male and Female Adolescents, School Students | South Africa | Positive |
| (Terefe & Larson, 1993) | Modern contraception use in Ethiopia: does involving husbands make a difference? | RCT | Female and Male Adolescents and Adults, Married | Ethiopia | Positive |
| (Thato et al., 2008) | Effects of the culturally-sensitive comprehensive sex education programme among Thai secondary school students | QE | Male and Female Adolescents, School Students | Thailand | Mixed |
| (Tilahun et al., 2015) | Couple based family planning education: changes in male involvement and contraceptive use among married couples in Jimma Zone, Ethiopia | QE | Males and Females of Reproductive Age | Ethiopia | Positive |
| (Timol et al., 2016) | Addressing adolescents' risk and protective factors related to risky behaviours: findings from a school-based peer-education evaluation in the Western Cape | QE | Male and Female Adolescents, School Students | South Africa | Mixed |
| (Torres et al., 2006) | A novel school based strategy for the prevention of HIV/AIDS, sexually transmitted disease (STDs), and teen pregnancies | RCT | Male and Female Adolescents, School Students | Mexico | Positive |
| (Townsend et al., 1987) | Sex education and family planning services for young adults: alternative urban strategies in Mexico | QE | Males and Females, Adolescents and Young Adults | Mexico | Positive |
| (Tu et al., 2008) | Long-term effects of a community-based program on contraceptive use among sexually active unmarried youth in Shanghai, China | QE | Males and Females, Adolescents and Young Adults | China | Negative |
| (Tuladhar & Stoeckel, 1982) | The relative impacts of vertical and integrated FP/MCH programs in rural Nepal | QE | Males and Females of Reproductive Age | Nepal | Mixed |
| (Turan, 1997) | The design and evaluation of an intervention to promote postpartum health in Istanbul: Does including fathers make a difference? | RCT | Male and Female Adults, First Time Parents | Turkey | Positive |
| (UERD, 1999) | An evaluation of the impact of community health field stations in Burkina Faso | QE | Males and Females of Reproductive Age | Burkina Faso | Positive |
| (Van et al., 2019) | Extra-curricular activities improved reproductive health knowledge of ethnic minority high school students in Vietnam | QE | Male and Female Adolescents, School Students | Vietnam | Positive |
| (van Rossem & Meekers, 1999) | An Evaluation of the Effectiveness of Targeted Social Marketing to Promote Adolescent Reproductive Health in Guinea | QE | Male and Female Adolescents | Guinea | Mixed |
| (Vernon & Dura, 2004) | Improving the reproductive health of youth in Mexico | QE | Males and Females of Reproductive Age | Colombia | Negative |
| (Villarruel et al., 2010) | Examining long-term effects of Cuidate-a sexual risk reduction program in Mexican youth | RCT | Males and Females of Reproductive Age: youth, parents, health service providers, schoolteachers, and other decision-makers in the community | Mexico | Mixed |
| (Walker et al., 2006) | HIV prevention in Mexican schools: prospective randomised evaluation of intervention | RCT | Male and Female Adolescents, School Students | Mexico | Negative |
| (Wall, 2012) | Promotion and integration of couples' HIV voluntary counseling and testing (CVCT) with other health services for HIV prevention | RCT | Male and Female Adults, HIV Treatment Seeking | Zambia | Mixed |
| (C. C. Wang et al., 1998) | Reducing pregnancy and induced abortion rates in China: family planning with husband participation | RCT | Males and Females of Reproductive Age | China | Positive |
| (B. Wang et al., 2005) | The potential of comprehensive sex education in China: findings from suburban Shanghai | QE | Male and Female Adolescents and Young Adults | China | Mixed |
| (WHO, 1987) | A prospective multicenter trial of the ovulation method of natural family planning. | QE | Males and Females of Reproductive Age | El Salvador, India, Philippines, Australia, Ireland | Mixed |
| (Yang et al., 1965) | Fertility and family planning in rural Korea | QE | Males and Females of Reproductive Age | China | Positive |
| (Zhang et al., 2017) | Effects of post-abortion family planning services on preventing unintended pregnancy and repeat abortion (INPAC): a cluster randomised controlled trial in 30 Chinese provinces | cRCT | Females and their Male Partners, Seeking Abortion | China | Positive |
| (Zhu et al., 2009) | Impact of post-abortion family planning services on contraceptive use and abortion rate among young women in China: a cluster randomised trial | cRCT | Females and their Male Partners, Seeking Abortion | China | Positive |

### Appendix 4.2: Study Characteristics Connected Papers

| First Author & Date | Connected Evaluation Study | Country | Population | Study Design | Study Aim | Intervention  (if named) | Risk of Bias Assessment |
| --- | --- | --- | --- | --- | --- | --- | --- |
| Ahmed (2013) | Ahmed (2015) | Bangladesh | Pregnant and Post-partum Women and Their Male Partners | Process Evaluation | Operations research to add postpartum family planning to maternal and neonatal health to improve birth spacing in Sylhet District, Bangladesh |  | Moderate |
| Akhter (1993) | Amatya (1994) | Bangladesh | Male Husbands of Female NORPLANT Trial Participants | Process Evaluation | A five-year clinical evaluation of NORPLANTR contraceptive subdermal implants in Bangladeshi acceptors | NORPLANT Counselling | Moderate |
| Baqui (2018) | Ahmed (2015) | Bangladesh | Pregnant and Post-partum Women and Their Male Partners | Process Evaluation | Impact of integrating a postpartum family planning program into a community-based maternal and newborn health program on birth spacing and preterm birth in rural Bangladesh |  | Low |
| Bertrand (1982) | Bertrand (1982) | Guatemala | Male Adolescents and Adults, targeted by Communications | Process Evaluation | Family planning communications and contraceptive use in Guatemala, El Salvador, and Panama |  | Moderate |
| Cooper (2014) | Ahmed (2015) | Bangladesh | Pregnant and Post-partum Women and Their Male Partners | Qualitative | Findings from the use of a narrative story and leaflet to influence shifts along the behavior change continuum toward postpartum contraceptive uptake in Sylhet District, Bangladesh |  | Moderate |
| Daniele (2017) | Daniele (2018) | Burkina Faso | Pregnant Women (aged 15-45) and their Male Partners | Process Evaluation | Involving Men in Maternity Care in Burkina Faso:An Intervention Study |  | Low |
| Doyle (2014) | Doyle (2018) | Rwanda | Male and Female Adults, Couples | Process Evaluation | Transforming gender roles in domestic andcaregiving work: preliminary findings fromengaging fathers in maternal, newborn, and childhealth in Rwanda | Bandebero | Moderate |
| Ghule (2015) | Raj (2016) | India | Male and Female Adults, Married couples | Qualitative | Barriers to use contraceptive methods among rural young married couples in Maharashtra, India: Qualitative findings | CHARM | Moderate |
| Harrington (2016) | Harrington (2019) | Kenya | Pregnant Females and their Male Partners | Qualitative | Gendered power dynamics and women's negotiation of family planning in a high HIV prevalence setting: a qualitative study of couples in western Kenya | MobileWatchXY | Low |
| Harrington (2017a) | Harrington (2019) | Kenya | Pregnant Females and their Male Partners | Process Evaluation | Evaluation of an mHealth SMS Dialogue Strategy to Meet Women's and Couples' Postpartum Contraceptive Needs in Kenya (Mobile WACh XY): a Randomized Controlled Trial | MobileWatchXY | Moderate |
| Harrington (2017b) | Harrington (2019) | Kenya | Pregnant Females and their Male Partners | Process Evaluation | Experience including men in a novel short message service (SMS) approach to improve postpartum family planning education and counseling in Kenya | MobileWatchXY | Moderate |
| Harrington (2019) | Harrington (2019) | Kenya | Pregnant Females and their Male Partners | Qualitative | Engaging men in an mHealth approach to support postpartum family planning among couples in Kenya: a qualitative study | MobileWatchXY | Moderate |
| Hartmann (2012) | Shattuck (2011) | Malawi | Male Adults | Qualitative | Changes in couples' communication as a result of a male-involvement family planning intervention | Malawi Male Motivators | Moderate |
| Jewkes (2010) | Jewkes (2008) | South Africa | Male and Female Adolescents and Young Adults | Qualitative | ‘I woke up after I joined Stepping Stones’: meanings of an HIV behavioural intervention in rural South African young people's lives | Stepping Stones | Moderate |
| Khan (2008) | Sebastian (2010) | India | Male and Female Adolescents and Adults.  (Pregnant Women, Male Partners, and Mothers-in-law) | Process Evaluation | Promoting healthy timing and spacing of births in India through a community-based approach |  | Moderate |
| Mantell (2014) | Mantell (2017) | South Africa | Male and Female Adults Living with HIV | Process Evaluation | Pregnancy Intent Among a Sample of Recently Diagnosed HIV-Positive Women and Men Practicing Unprotected Sex in Cape Town, South Africa | Emtonjeni | Low |
| McCarthy (2018a) | McCarthy (2018) | Tajikistan | Male and Female Adolescents and Young Adults | Process Evaluation | App instant messaging to increase acceptability of effective contraception among young people in Tajikistan: results from a randomised controlled trial | TFPA’s ‘Healthy Lifestyles’ app | Moderate |
| McCarthy (2018b) | McCarthy (2018) | Tajikistan | Male and Female Adolescents and Young Adults | Process Evaluation | Development of an intervention delivered by mobile phone aimed at decreasing unintended pregnancy among young people in three lower middle income countries | TFPA’s ‘Healthy Lifestyles’ app | Moderate |
| McCarthy (2019) | McCarthy (2018) | Tajikistan | Male and Female Adolescents and Young Adults | Qualitative | Changing young people's attitudes towards effective contraception using mobile phone messaging | TFPA’s ‘Healthy Lifestyles’ app | High |
| Nair (2019) | Raj (2016) | India | Male and Female Adults, Married couples | Qualitative | Health care providers' perspectives on delivering gender equity focused family planning program for young married couples in a cluster randomized controlled trial in rural Maharashtra, India | CHARM | Moderate |
| Ngure (2012) | Ngure (2009) | Kenya | Male and Female Adults, HIV serodiscordant couples | Process Evaluation | Correlates of contraceptive use among HIV discordant couples in Kenya |  | Moderate |
| Ross (1966) | Yang (1965) | China | Males and Females of Reproductive Age | Process Evaluation | The AID computer programme, used to predict adoption of family planning in Koyang | The Koyang Study | Moderate |
| Turan (2001) | Sahip (2007) | Turkey | Male Adults, Expectant Fathers | Process Evaluation | Including expectant fathers in antenatal education programmes in Istanbul, Turkey |  | Moderate |

### Appendix 5.0: Risk of Bias Assessments

### Appendix 5.1: Risk of Bias in Included Randomised Controlled Trials

| First Author & Year | Domain 1 Randomisation Process | Domain 2a Intervention Assignment | Domain 2b Intervention Adherence | Domain 3 Missing Data | Domain 4 Outcome Measurement | Domain 5 Selective Reporting | Overall Judgement |
| --- | --- | --- | --- | --- | --- | --- | --- |
| Acharya (2014) | Low | Some concerns | Low | Some concerns | Some concerns | Some concerns | Some Concerns |
| Aninanya (2015) | Low | Some concerns | Some concerns | Some concerns | Some concerns | Some concerns | Some Concerns |
| Atwood (2012) | Some concerns | Low | Low | Some concerns | Some concerns | Some concerns | Some Concerns |
| Bachanas (2016) | Some concerns | Some concerns | Some concerns | Some concerns | Some concerns | Some concerns | Some Concerns |
| Caceres  (1994) | Some concerns | Some concerns | Some concerns | Low | Some concerns | Some concerns | Some Concerns |
| Chong  (2013) | Low | Some concerns | Some concerns | Some concerns | Some concerns | Some concerns | Some Concerns |
| Coffman (2011) | Some concerns | Some concerns | Some concerns | Low | Some concerns | Some concerns | Some Concerns |
| Conboy  (2003) | Some concerns | Some concerns | Some concerns | Low | High | Some concerns | High |
| Cowan  (2010) | Some concerns | Low | Some concerns | Low | Some concerns | Some concerns | Some Concerns |
| Daniele  (2018) | Low | Low | Some concerns | Low | Some concerns | Low | Some Concerns |
| Decat  (2014) | High | Some concerns | Some concerns | High | Some concerns | Some concerns | High |
| Doyle  (2011) | Some concerns | Some concerns | Some concerns | Some concerns | Some concerns | Low | Some Concerns |
| Doyle  (2018) | Some concerns | Some concerns | Low | Low | Some concerns | Low | Some Concerns |
| El‐Khoury (2016) | Low | Low | Some concerns | Low | Some concerns | Some concerns | Some Concerns |
| Firestone (2016) | Low | Some concerns | High | Some concerns | Some concerns | Some concerns | High |
| Fitzgerald (1999) | Low | Low | Some concerns | Low | Some concerns | Some concerns | Some Concerns |
| Fleming (2018) | Low | Low | Some concerns | Low | Low | Low | Some Concerns |
| Gallegos (2008) | Some concerns | Low | Low | Low | Some concerns | Low | Some Concerns |
| Gao  (2001) | High | Low | Low | Some concerns | Some concerns | Some concerns | High |
| Harrington (2017) | Low | Low | Low | Low | Some concerns | Low | Some Concerns |
| Harrington (2019) | Low | Low | Low | Low | Some concerns | Low | Some Concerns |
| Jewkes  (2008) | Some concerns | Low | High | Low | Some concerns | Low | High |
| Johnson  (2017) | Some concerns | Some concerns | Some concerns | High | Some concerns | Some concerns | High |
| Kaljee  (2012) | Low | Some concerns | High | Low | Some concerns | Some concerns | High |
| Kemigisha (2019) | Some concerns | Low | Some concerns | Low | Some concerns | Low | Some Concerns |
| Lemani  (2017) | High | Low | Low | Low | Some concerns | Low | High |
| Lutalo  (2010) | High | Some concerns | High | Some concerns | Some concerns | Some concerns | High |
| Mantell  (2017) | Low | Low | Low | Low | Low | Low | Low |
| Mark  (2007) | Low | Some concerns | Low | Low | Some concerns | Some concerns | Some Concerns |
| Martiniuk (2003) | High | Low | High | Some concerns | Some concerns | Some concerns | High |
| Mbizvo  (1997) | Some concerns | Some concerns | High | Low | Some concerns | Low | High |
| McCarthy (2018) | Low | High | Some concerns | Low | Some concerns | Low | High |
| Medina  (1980) | Some concerns | Low | Some concerns | High | Low | Some concerns | High |
| Ojengbede (2009) | Some concerns | Low | Low | Low | Some concerns | Some concerns | Some Concerns |
| Onono  (2015) | Some concerns | Low | Low | Low | Some concerns | Some concerns | Some Concerns |
| Pham  (2012) | Some concerns | Low | High | Some concerns | Some concerns | High | High |
| Pinchoff (2019) | Some concerns | Low | Some concerns | Low | Some concerns | Low | Some Concerns |
| Pivatti  (2019) | Low | Some concerns | Some concerns | Low | Some concerns | Low | Some Concerns |
| Prasertsawat (2002) | High | Some concerns | Some concerns | Low | High | Some concerns | High |
| Raj  (2016) | Low | Low | Low | Low | Some concerns | Low | Some Concerns |
| Ross  (2007) | Some concerns | Some concerns | Some concerns | Some concerns | Some concerns | Low | Some Concerns |
| Rusakaniko (1997) | Some concerns | Some concerns | High | Low | Some concerns | Some concerns | High |
| Schuler  (2012) | Some concerns | High | High | High | Some concerns | Low | High |
| Schuler  (2015) | Some concerns | High | High | High | Some concerns | Some concerns | High |
| Shattuck (2011) | Low | Some concerns | Some concerns | Low | Some concerns | Some concerns | Some Concerns |
| Shuey  (1999) | Low | Some concerns | Some concerns | Some concerns | Some concerns | Some concerns | Some Concerns |
| Singh  (2016) | Low | Low | Low | Some concerns | Some concerns | Some concerns | Some Concerns |
| Stephenson (2011) | Some concerns | Low | Some concerns | Low | Low | Some concerns | Some Concerns |
| Tang  (2009) | Low | Low | Low | Low | Some concerns | Some concerns | Some Concerns |
| Taylor  (2014) | High | Some concerns | Some concerns | High | Some concerns | Some concerns | High |
| Terefe  (1993) | Low | Some concerns | Some concerns | Low | Some concerns | Some concerns | Some Concerns |
| Torres  (2006) | Low | Some concerns | Some concerns | Low | Some concerns | Low | Some Concerns |
| Turan  (1997) | Some concerns | Some concerns | Some concerns | Low | Some concerns | Some concerns | Some Concerns |
| Villarruel (2010) | Some concerns | Low | Some concerns | Low | Some concerns | Some concerns | Some Concerns |
| Walker  (2006) | Some concerns | Some concerns | Some concerns | Some concerns | Some concerns | Some concerns | Some Concerns |
| Wall  (2012) | Some concerns | Some concerns | Low | Some concerns | Some concerns | Low | Some Concerns |
| Wang  (1998) | Some concerns | Some concerns | Some concerns | Low | Some concerns | High | High |
| Zhang  (2017) | Some concerns | Some concerns | Some concerns | Some concerns | Some concerns | Low | Some Concerns |
| Zhu  (2009) | Some concerns | Some concerns | High | High | Some concerns | Some concerns | High |

Legend:

| Low | The study is judged to be at low risk of bias for all domains for this result. |
| --- | --- |
| Some concerns | The study is judged to raise some concerns in at least one domain. For this result, but not to be at High risk of bias for any domain. |
| High | The study is judged to be at high risk of bias in at least one domain for this result.  Or  The study is judged to have some concerns for multiple domains  in a way that substantially lowers confidence in the result. |

### Appendix 5.2: Risk of Bias in Quasi-Experimental Studies

| First Author & Year | Domain 1 Confounding | Domain 2 Participant Selection | Domain 3 Classification of Intervention | Domain 4 Deviation from Intervention | Domain 5 Missing Data | Domain 6 Outcome Measurement | Domain 7 Selective Reporting | Overall Judgement |
| --- | --- | --- | --- | --- | --- | --- | --- | --- |
| Agha (2002) | Moderate | Serious | Moderate | Serious | Moderate | Moderate | Low | Serious |
| Agha (2007) | Moderate | Low | Moderate | Moderate | Serious | Serious | Serious | Serious |
| Ahmed (2015) | Moderate | Low | Low | Moderate | Low | Moderate | Low | Moderate |
| Ajuwon (2007) | Serious | Low | Low | Moderate | Serious | Moderate | Low | Serious |
| Almeida (2019) | Serious | Low | Low | Moderate | Serious | Moderate | Low | Serious |
| Amatya (1994) | Serious | Serious | Serious | Moderate | Serious | Moderate | Low | Serious |
| Amin (2005) | Low | Low | Low | Moderate | Serious | Moderate | Low | Serious |
| Andrade (2009) | Moderate | Moderate | Serious | Moderate | Serious | Moderate | Low | Serious |
| Bertrand (1982) | Serious | Low | Low | Serious | Moderate | Moderate | Low | Serious |
| Black (1976) | Serious | Low | Serious | Serious | Moderate | Moderate | Low | Serious |
| Boti  (2019) | Moderate | Low | Low | Moderate | Low | Moderate | Low | Moderate |
| Brieger (2001) | Serious | Low | Moderate | Serious | Moderate | Moderate | Low | Serious |
| Campero (2011) | Moderate | Low | Serious | Moderate | Serious | Moderate | Moderate | Serious |
| Campero (2019) | Moderate | Low | Moderate | Moderate | Serious | Moderate | Low | Serious |
| Chi  (2015) | Moderate | Moderate | Serious | Moderate | Moderate | Moderate | Low | Serious |
| Daniel (2012) | Moderate | Low | Moderate | Serious | Serious | Moderate | Low | Serious |
| Debpuur (2002) | Moderate | Low | Moderate | Serious | Moderate | Moderate | Low | Serious |
| Decat (2015) | Moderate | Moderate | Moderate | Moderate | Serious | Moderate | Low | Serious |
| Diop (2004) | Moderate | Low | Low | Moderate | Moderate | Moderate | Low | Moderate |
| Eggleston (2000) | Moderate | Low | Moderate | Moderate | Moderate | Moderate | Low | Moderate |
| Erulkar (2004) | Moderate | Moderate | Low | Low | Low | Low | Moderate | Moderate |
| Exner (2009) | Moderate | Low | Low | Moderate | Moderate | Moderate | Low | Moderate |
| Fisek (1978) | Moderate | Moderate | Serious | Moderate | Serious | Serious | Low | Serious |
| Foreit (1992) | Critical | No information | Low | No information | No information | Serious | Low | Critical |
| Ha  (2005) | Moderate | Low | Moderate | No information | No information | Moderate | Moderate | Moderate |
| Hallman (2011) | Serious | Low | Low | Moderate | No information | Moderate | Serious | Serious |
| Halpern (2008) | Moderate | Low | Moderate | Serious | Moderate | Moderate | Low | Moderate |
| He  (2012) | Moderate | Low | Low | Moderate | Moderate | Moderate | Low | Moderate |
| Kanesathasan (2008) | Low | Low | Serious | Moderate | Serious | Moderate | Serious | Serious |
| Katz (1998) | Serious | Low | Low | No information | Moderate | Moderate | Low | Serious |
| Khan (1976) | Critical | No information | Low | No information | Critical | Critical | Critical | Critical |
| Khatun (2011) | Serious | Low | Low | No information | Low | Moderate | Low | Serious |
| Kim  (2001) | Moderate | Low | Low | Moderate | Low | Moderate | Low | Moderate |
| Kulathinal (2019) | Moderate | Low | Low | Moderate | Moderate | Moderate | Low | Moderate |
| Lou  (2004) | Moderate | Low | Low | Low | Low | Moderate | Low | Moderate |
| Lou  (2006) | Moderate | Low | Low | Moderate | Moderate | Moderate | Moderate | Moderate |
| Luck (2000) | Moderate | Low | Moderate | Moderate | Moderate | Moderate | Low | Moderate |
| Magnani (2001) | Moderate | Low | Low | Serious | Serious | Low | Moderate | Serious |
| Mantell (2006) | Serious | Serious | Moderate | Serious | Serious | Moderate | Moderate | Critical |
| Mathur (2004) | Serious | Serious | Low | Serious | No information | Moderate | Low | Serious |
| Mba (2007) | Serious | Low | Low | Low | Low | Moderate | Low | Serious |
| Mbonye (2003) | Serious | Serious | Serious | Serious | Serious | Moderate | Low | Serious |
| Ngure (2009) | Moderate | Serious | Low | Serious | Serious | Moderate | Moderate | Serious |
| Odeyemi (2011) | Serious | Low | Low | Moderate | Low | Moderate | Serious | Serious |
| Oyugi (2017) | Serious | Low | Moderate | Moderate | Serious | Moderate | Low | Serious |
| Ozcebe (2003) | Serious | Serious | Moderate | No information | No information | Moderate | Low | Serious |
| Ozgur (2000) | Serious | Low | Low | Moderate | Moderate | Moderate | Low | Serious |
| Phillips (2012) | Moderate | Low | Moderate | Serious | Moderate | Moderate | Low | Serious |
| Pineda (1983) | Serious | Serious | Low | Moderate | Low | Low | Low | Serious |
| Pitt  (1999) | Moderate | Moderate | Serious | Moderate | Serious | Moderate | Low | Serious |
| Rogers (1999) | Moderate | Moderate | Moderate | Moderate | Serious | Moderate | Low | Serious |
| Sahip (2007) | Serious | Serious | Moderate | Serious | Serious | Moderate | Low | Serious |
| Sebastian (2010) | Serious | Serious | Moderate | Moderate | Serious | Moderate | Low | Serious |
| Speizer (2001) | Moderate | Low | Moderate | Moderate | Serious | Moderate | Low | Serious |
| Subramanian (2018) | Moderate | Moderate | Moderate | Serious | Serious | Moderate | Low | Serious |
| Thato (2008) | Moderate | Moderate | Moderate | Moderate | Serious | Moderate | Low | Serious |
| Tilahun (2015) | Serious | Moderate | Moderate | Serious | Low | Moderate | Moderate | Serious |
| Timol (2016) | Serious | Serious | Low | Serious | Moderate | Moderate | Low | Serious |
| Townsend (1987) | Serious | Serious | Serious | Serious | Serious | Moderate | Low | Serious |
| Tu  (2008) | Moderate | Low | Low | Serious | Serious | Moderate | Moderate | Serious |
| Tuladhar (1982) | Moderate | Moderate | Moderate | Moderate | Serious | Moderate | Moderate | Serious |
| UERD (1999) | No information | No information | No information | No information | No information | No information | No information | No Informarion/Critical |
| Van Hung (2019) | Serious | Low | Low | Moderate | Serious | Moderate | Low | Serious |
| Van Rossem (1999) | Moderate | Serious | Moderate | Serious | Moderate | Moderate | Moderate | Serious |
| Vernon (2004) | Moderate | Moderate | Low | Serious | Moderate | Moderate | Moderate | Serious |
| Wang (2005) | Moderate | Moderate | Moderate | Low | Moderate | Low | Low | Moderate |
| WHO (1987) | Critical | Critical | Critical | Serious | Critical | Moderate | Low | Critical |
| Yang (1965) | Serious | Low | Serious | Serious | Moderate | Moderate | Serious | Serious |

Legend:

| Low | The study is comparable to a well-performed randomized trial overall. |
| --- | --- |
| Moderate | The study is sound for a non-randomized study overall. |
| Serious | The study has some important problems. |
| Critical | The study is too problematic to provide any useful evidence on the effects of intervention. |
| No Information | Insufficient information to make a judgement about risk of bias for this study. |

### Appendix 5.3: Risk of Bias in Connected Papers Process Evaluations

| First Author & Year | | Ahmed (2013) | | Akhter (1993) | | Baqui (2018) | | Bertrand (1982) | | Daniele (2017) | | Doyle (2014) | | Harr’ton (2017a) | Harr’ton (2017b) | Khan (2008) | Mantell (2014) | McCarthy (2018) | McCarthy (2018) | Ngure (2012) | Ross (1966) | Turan (2001) |
| --- | --- | --- | --- | --- | --- | --- | --- | --- | --- | --- | --- | --- | --- | --- | --- | --- | --- | --- | --- | --- | --- | --- |
| EEPI RoB Tool Categories |  | | | | | | | | | | | | | | | | | | | | | |
| Is the context of the study adequately described? | YES | | YES | | YES | | YES | | YES | | YES | | YES | | YES | YES | YES | YES | YES | YES | NO | YES |
| Are the aims of the study clearly reported? | YES | | YES | | YES | | YES | | YES | | YES | | YES | | YES | YES | YES | YES | YES | YES | YES | YES |
| Is there an adequate description of the sample used in the study and how the sample was identified and recruited? | YES | | YES | | YES | | YES | | YES | | YES | | YES | | YES | YES | YES | YES | YES | YES | YES | YES |
| Is there an adequate description of the methods used in the study to collect data? | YES | | YES | | YES | | NO | | YES | | YES | | YES | | YES | YES | YES | YES | YES | YES | NO | NO |
| Is there an adequate description of the methods of data analysis? | NO | | NO | | YES | | NO | | YES | | NO | | YES | | YES | YES | YES | YES | NO | YES | YES | NO |
| Is the study replicable from this report? | NO | | NO | | YES | | NO | | YES | | NO | | YES | | YES | YES | YES | YES | YES | YES | NO | NO |
| Do the authors avoid selective reporting bias? | YES | | YES | | YES | | YES | | YES | | YES | | YES | | YES | YES | YES | YES | YES | YES | YES | YES |
| Are there ethical concerns about the way the study was YYdone? | NO | | NO | | NO | | NO | | NO | | NO | | NO | | NO | NO | NO | NO | NO | NO | NO | NO |
| Is there sufficient justification for why the study was done the way it was? | YES | | YES | | YES | | YES | | YES | | YES | | YES | | YES | YES | YES | YES | YES | YES | YES | YES |
| Was the choice of research design appropriate for addressing the research question(s) posed? | YES | | YES | | YES | | YES | | YES | | YES | | YES | | YES | YES | YES | YES | YES | YES | YES | YES |
| Have sufficient attempts been made to establish the reliability of data collection methods and tools? | YES | | YES | | YES | | NO | | YES | | NO | | NO | | NO | YES | YES | NO | NO | NO | NO | NO |
| Have sufficient attempts been made to establish the validity of data collection tools and methods? | NO | | NO | | YES | | NO | | YES | | NO | | NO | | NO | NO | YES | NO | NO | NO | YES | NO |
| Have sufficient attempts been made to establish the reliability of data analysis? | YES | | NO | | YES | | NO | | YES | | NO | | NO | | NO | NO | YES | YES | NO | YES | YES | No |
| Have sufficient attempts been made to establish the validity of data analysis? | NO | | NO | | YES | | NO | | YES | | NO | | NO | | NO | NO | YES | YES | NO | NO | YES | NO |
| To what extent are the research design and methods employed able to rule out any other sources of error/bias which would lead to alternative explanations for the findings of the study? | A little | | A little | | A lot | | A lot | | A lot | | A little | | A little | | A little | A lot | A lot | A lot | A lot | A lot | A lot | A little |
| Overall Risk of Bias Judgement | MODERATE | | MODERATE | | LOW | | MODERATE | | LOW | | MODERATE | | MODERATE | | MODERATE | MODERATE | LOW | MODERATE | MODERATE | MODERATE | MODERATE | MODERATE |

### Appendix 5.4: Risk of Bias in Connected Papers Qualitative Studies

| **RoB Categories** | **Clear description of the study’s methods and procedures** | **Rigour of the qualitative study in conduct** | **Reportage of assumptions, values, biases** | **Attempts to address biases** | **Transferability of results** | **Data situated within political, institutional, cultural or social context** | **Overall Risk of Bias Judgement** |
| --- | --- | --- | --- | --- | --- | --- | --- |
| **First Author & Date** |  |  |  |  |  |  |  |
| Cooper (2014) | Yes | Partially | No | No | No | Yes | Moderate |
| Ghule (2015) | Yes | Partially | No | Partially | Partially | Partially | Moderate |
| Harrington (2016) | Yes | Yes | Partially | Yes | Yes | Yes | Low |
| Harrington (2019) | Yes | Yes | No | Partially | Yes | Partially | Moderate |
| Hartmann (2012) | Yes | No | No | No | Partially | Yes | Moderate |
| Jewkes (2010) | Yes | No | No | Partially | Partially | Yes | Moderate |
| McCarthy (2019) | Partially | No | No | No | Partially | Partially | High |
| Nair (2019) | Yes | Yes | No | Yes | Partially | Yes | Moderate |

Appendix 6.0: Examples of Data Relating to Barriers and Facilitators of Effective Models of FP in Connected Papers

| SYSTEM-LEVEL BARRIERS AND ENABLERS | | |
| --- | --- | --- |
| INDIVIDUAL SYSTEMS |  |  |
| Category | Studies Presenting Data | Examples of Data |
| Socioeconomic factors | Bertrand, 1982; Turan, 2001 | “This suggests that working women may have greater contact with the outside world, including the mass media carrying family planning m messages, or perhaps that they feel a greater need for this type of information." (Bertrand, 1982)  “Only 22 men from the couples’ group (26.2 per cent) said that they had attended one or more sessions. [These men] tended to be more educated, older and more likely to have some form of health insurance than men who did not participate (all p’s from x2 < .05). Their wives were also more likely to be more educated, be working outside the home and have wider social support networks than the wives of men who did not attend (all p’s from x2 < .05)." (Turan, 2001) |
| Attitudes values and beliefs about FP (including attitudes about services) | Cooper, 2014; Daniele, 2017; Doyle, 2014; Ghule 2015; Hartmann, 2012; Khan, 2008; McCarthy, 2019 | “Husbands reported that condom use was for outside of marriage and in extramarital affairs, for both pregnancy and HIV prevention. … Husbands in particular seemed to prefer natural methods to avoid pregnancy. This approach was viewed as healthier and simpler, and thus better.” (Ghule, 2015)  "In discussing method choice, men often described the reasons for choosing a particular method including cost, economic circumstances, and access. ‘I felt this method would not give me a tough time like going as far as the health centre for us to get an injection or even pills, yea, because these [condoms] are readily found’” (Hartmann, 2012)  No participants said that their behaviour changed as a result of the app or messages but reiterated that they improved their attitudes towards contraception. Most said that the content changed their intention or “future behaviour”, in that they intend to use contraception and talk to future wives/husbands about family planning." (McCarthy, 2019) |
| Perceived gender and cultural norms | Daniele, 2017; Doyle, 2014; Ghule, 2015; Harrington, 2016; Hartmann, 2012; Jewkes, 2010; McCarthy, 2018b; McCarthy 2019 | “In response to both perceived and expressed male resistance to family planning, many women did not feel that they could fulfil the gender role of initiating communication or interest in contraception for fear of consequences.” (Harrington, 2016)  “Over one quarter (11, or 29%) of women in the sample disclosed that they had intentionally concealed, or were currently concealing, contraceptive use from partners. … When discussing motivations for covert use, women emphasised men’s cultural expectations of childbearing and the difficulties in challenging these views." (Harrington, 2016)  “During the interviews, men indicated that they sometimes felt pressurised into sex by cultural scripts that dictated appropriate male behaviour in particular contexts, scripts that they often wanted to deviate from but in a polite manner." (Jewkes, 2010) |
| Attitudes about sexual pleasure | Ghule, 2015; Khan, 2008 | “Husbands also described how condoms inhibited sexual pleasure, and that prevented them from continuing use of condoms. … Sometimes husbands stopped condom use at the wife’s request, and for her enjoyment.” (Ghule, 2015)  “…apart from the general complaint of lack of sexual pleasure, many (particularly women) believe that a condom could cause an infection leading to stoppage of the menstrual cycle.” (Khan, 2008) |
| Migrant status  (men working away from home) | Cooper (2014); Daniele 2017 | “The most commonly cited factor preventing individuals from moving from this stage to the practicing stage, cited by twelve respondents, was that their husbands were currently working abroad." (Cooper, 2014)  “Lack of time due to work or being away was the chief reason given for not attending by men who did not attend one or more sessions.” (Daniele, 2017) |
| Urban/Rural Residence | Bertrand (1985) | “one might expect to find certain selective attention patterns among those groups for which family planning messages might have the greatest salience (women who are married or in consensual union, and well along in the reproductive cycle). While there is some evidence of such patterns, the most marked differences relate to place of residence, education, and employment status. This presents communicators with the classic challenge-how to reach the most isolated and least educated." (Bertrand, 1985) |
| Sex of Existing Children | Ghule, 2015; Nair, 2019; Ross, 1966 | “Sons are preferred over daughters by both husbands and wives. Family size and decisions about use of contraception often depend upon the sex of first child, with contraception being less likely if a boy has not been born." (Ghule, 2015)  “Providers observed that despite intervention efforts to reduce son preference, there had been very little impact on this issue as reported by respondents”. (Nair, 2019) |
| Reproductive History (and intentions for future childbearing) | Ghule, 2015; Harrington, 2016; Ross, 1966 | “Women are held responsible if a child is not born early in the marriage and can be mistreated for it. Such beliefs can affect contraceptive use in early marriage and prior to the birth of the first child”. (Ghule, 2015)  "Expectations of childbearing in marriage were seen as inherently limiting to family planning use and contributed to difficulty communicating about and negotiating family planning." (Harrington, 2016)  “Both men and women desired the second child three or more years after the birth of first child. The advantages of spacing between the first and second child appealed to all, including husbands and mothers-in-law.” (Khan, 2008) |
| Co-residence with extended family  (the influence of family) | Cooper (2014); Daniele (2017); Ghule, 2015; McCarthy 2018b; McCarthy 2019; Khan, 2008 | “Husbands and extended family may actively prohibit female contraception in their effort to ensure the birth of sons." (Ghule, 2015)  “Tajik providers commented on how … the mother-in-law has a great amount of influence on her daughter-in-law’s contraceptive use." (McCarthy, 2018b)  “Four participants did not want their parents or friends to know that they had the app on their phone. Three of these participants said that they had positive discussions with their parents about contraception.” (McCarthy, 2019) |
| Relationship status/quality | Daniele, 2017; Harrington 2019; Hartmann, 2012 | “Some women reported that their husbands had become more attentive as a result of their participation in the study. Similarly, some men felt that the project had contributed to improving and bringing harmony to their relationship” (Daniele, 2017)  “Similar proportions of male and female participants described new communication roles for men and women within their relationship. However, these roles differed in form. Men were described as taking new leadership roles and women, who were not previously conferred with, were now consulted.” (Hartmann, 2012)  "However, asking permission and “sweet talk” required direct communication with male partners about contraception. In many cases, women portrayed the power dynamics in their relationships as excluding such direct communication.” (Harrington, 2016) |
| Marital status/history/type | Daniele, 2017; Khan, 2008; McCarthy, 2018b | “Co-habitation was a pre-requisite for enrolment in this study, which may have meant that couples had a closer and more committed relationship, in which the man might have been more willingly become involved in the woman’s health care. In polygamous marriages, men may have felt less invested in the health care of each wife, or held more traditional attitudes, leading to a reluctance to participate.” (Daniele, 2017)  “Newly married couples experience huge pressure for an early pregnancy. Findings from the in-depth interviews allude to the circumstances in which women put pressure on their husbands for an early pregnancy or vice versa.” (Khan, 2008)  “In Palestine and Tajikistan, participants spoke of pressure to begin childbearing soon after marriage” (McCarthy, 2018b) |
| HIV/AIDS/STI status | Mantell, 2014; Ngure 2012 | “HIV positive status was associated with increased contraceptive use at each time point relative to those HIV negative. Among HIV negative women contraceptive use remains largely constant. Additional factors effecting women's reported use of non-barrier contraception were being married and having two or more children. Going through HIV treatment and being with a partner who was HIV positive were significant predictors of condom use.” (Ngure, 2012)  “Among women, using hormonal contraceptives was associated with lower odds of pregnancy intent (AOR = 0.08; P = 0.004), whereas main partner being aware of her HIV-positive status (AOR = 5.80; P = 0.050) and partner having immediate pregnancy intent were associated with higher odds (AOR = 13.24).” (Mantell, 2014) |
| Knowledge about FP | Cooper, 2014; Daniele, 2017; Harrington 2017a; Harrington, 2019; Hartmann, 2012; Khan, 2008; McCarthy, 2018b | “Qualitative studies from the study region, including formative work for the current study, have enumerated men’s concerns and misperceptions about contraceptive side effects and harms.” (Harrington 2017a)  "Both men and women cited concerns about contraceptive side effects and potential harms as the most prominent barrier to postpartum family planning. Several men asserted that repetitive messaging would be most effective at increasing awareness and knowledge among men." (Harrington, 2019)  "Besides a lack of information, inaccurate information was causing many unwanted pregnancies.” (Khan, 2008) |
| Communication and decision-making | Daniele 2017; Doyle, 2014; Ghule, 2015; Harrington, 2016; Harrington, 2017a; Hartmann, 2012, Jewkes, 2010; McCarthy, 2018b; Nair, 2019; Turan 2001 | “Most men and women reported communicating and deciding together about the use of a PPFP method. Some women felt that having their partner’s agreement on this issue was important for the couple’s wellbeing. … Regardless of who was most keen on commencing PPFP, however, in almost all cases the woman decided on which method to use.” (Daniele 2017)  "Despite men’s and women’s agreement that the decision to use contraception was ultimately male-dominated, women were expected to initiate interest in and take responsibility for family planning as the ones who “bear the burden” of pregnancy, childbirth, and the care of young children.” (Harrington, 2016)  “Male participants expressed an overwhelming preference for—and sense of entitlement to—inclusion in FP decision-making. … Men perceived that the imbalance of FP knowledge between men and women not only contributes to community FP stigma, but negatively affects couple communication.” (Harrington 2017b)  “The empowerment and greater communication skills opened up new possibilities for discussing sex with older people. … Young men spoke of talking to their parents and sharing discussions from the workshops, and being able to do so without appearing (culturally) inappropriately knowledgeable or disrespectful in the way they opened conversations. … Men and women participants explained that the improved communication brought ‘peace’ to their relationships " (Jewkes, 2010) |
| EXTERNAL SYSTEMS | | |
| Category | Studies Presenting Data | Examples of Data |
| Social Network Influences | Cooper, 2014; Daniele 2017; Doyle, 2014; Ghule, 2015; Harrington 2016; Hartmann, 2012; Jewkes, 2010; Turan, 2001 | “Many postpartum women, husbands, and mothers/mothers-in- law reported discussing Asma’s Story with spouses, friends, and other family members, encouraging them to practice the recommended PPFP behaviors. Eighteen percent of the 40 women interviewed were not only using a modern contraceptive method, but had also advocated for others to do so.” (Cooper, 2014)  “Half of the women noted that their husband had previously opted not to use family planning but reversed their decision after meeting with the motivator. ‘Yes the study helped us because my husband warned me against using contraception. Suddenly after interacting with the male motivator, he reversed his advice because he told me to start using injectable contraception.’ (Hartmann, 2012)  "Some of the participants talked of changes in their peer relations, the sharing of new knowledge and attitudes and new confidence in their ideas and ability to communicate these." (Jewkes, 2010) |
| Gender, cultural and religious norms | Jewkes, 2010; Daniele, 2017; Doyle, 2014; Ghule 2015; Harrington 2016; Khan, 2008; Nair, 2019; McCarthy, 2018b | “It seems likely that a key reason why Stepping Stones failed to impact on HIV incidence in women, even though the study was adequately powered to do this (unlike the case for men), lies in limited impact of the intervention in instilling a feminist consciousness in women. … While the workshops did potentially empower women by exposing them to new ideas on gender relations, these had to compete against a cultural backdrop which had provided messages, seeped in the prevailing patriarchal gender order, about how to be women for many years” (Jewkes, 2010)  “Despite men’s and women’s agreement that the decision to use contraception was ultimately male-dominated, women were expected to initiate interest in and take responsibility for family planning as the ones who “bear the burden” of pregnancy, childbirth, and the care of young children.” (Harrington, 2016)  “Tajik providers commented on how religion does not accept sexual activity before marriage and that the mother-in-law has a great amount of influence on her daughter-in-law’s contraceptive use." (McCarthy, 2018b) |
| Health Systems & Services | Bacqui (2018); Daniele (2017); Doyle, 2014; Nair, 2019 | “A concern related to FP-MNCH [maternal and child health] integration activities is that while adding family planning may improve FP outcomes, the addition of new tasks and activities may undermine MNH service delivery performance and affect outcomes. Our analyses indicate that adding family planning to the maternal and neonatal health intervention package did not negatively influence MNH coverage or selected newborn care practices.” (Bacqui, 2018)  “Several providers, and interviewed men and women said that men may have been put off accepting the invitation because the idea of them going to health centres was so unfamiliar. … Some men (all of whom nevertheless attended sessions) mentioned the general concern that they may not be well-received by health workers.” (Daniele, 2017) |
| FP Supply Chain | Ahmed, 2013; Ross, 1966 | “HFS-enrolled women began requesting contraceptives from CHWs, and so we added contraceptive distribution to the study design as an opportunity to potentially enhance intervention impact. In this culturally conservative area, women’s movement outside the home is curtailed, even more so than in other areas of Bangladesh, which limits their contraceptive access.” (Ahmed, 2013) |
| PROCESS-LEVEL BARRIERS AND FACILITATORS | | |
| Category | Studies Presenting Data | Examples of Data |
| Intervention acceptability | Harrington 2017a; Harrington, 2019 | “Home visits replaced phone screening for most men, as staff found that discussing family planning by phone was culturally inappropriate.” (Harrington 2017a)  “In addition to repetition, most men felt that they needed the “truth” about FP, and SMS could be a vehicle for accurate information. … A few women felt that in order for SMS to be well-received by men, they should be primed with in-person FP education; others simply felt men must not be “surprised” by FP-related messaging.” (Harrington, 2019) |
| Intervention costs, sustainability, replicability | McCarthy, 2018b | “A few participants in Tajikistan said that access was sometimes restricted due to insufficient funds to support Internet connectivity." (McCarthy, 2018b) |
| Quality of delivery | Ahmed (2013); Nair (2019); Daniele, 2017; Khan, 2008 | “Based on programmatic experience, the HFS team concluded that fewer visits would lead to gaps in continuation of breastfeeding and the transition from LAM to other modern contraceptive methods." (Ahmed, 2013)  "In several cases, men/couples took part in the sessions thanks to the flexibility of health workers and their willingness to accommodate the men’s wishes.” (Daniele, 2017)  “Despite extensive training and careful selection of providers for the intervention, a few providers held biases regarding contraception and gender equity, which affected their intervention delivery. Some felt that newly married couples did not need the intervention, as they would not desire contraception due to pronatalist preferences and expectations of these couples." (Nair, 2019) |
| Provider-preparedness (and provider characteristics) | Daniele (2017); Ghule, 2015; Harrington, 2019; McCarthy, 2018b; Khan, 2008; | “Most of the ANMs do not routinely counsel women on postpartum contraception or LAM during their ANC visits. Discussions on postpartum care or visiting women during the first week after delivery are also rare – less than 10 percent of the women surveyed had been educated about LAM or postpartum contraception by the health providers or AWs." (Khan, 2008)  “Other than the desire for accurate FP information, this was the most frequently-discussed benefit of a two-way SMS intervention… Participants in this study emphasized the need for FP information to be introduced by a trusted source from outside the couple, bypassing male distrust of women’s FP knowledge." (Harrington, 2019)  “Some participants in Tajikistan, however, did not want information in the form of stories because they value the advice from a specialist over advice from a peer." (McCarthy, 2018b) |
| Participant recruitment, retention & representativeness | Harrington 2017a; Daniele, 2017; Nair, 2019; Turan, 2001 | “Women with enrolled partners were more likely to share their mobile phones with their partner (26.2% vs. 8.4%, p=.001)" (Harrington, 2017a)  “Most providers experienced difficulty in organizing the couple’s session, and consequently participation of women in these sessions was limited." (Nair, 2019)  “The participants in this study generally agreed that it would not be easy to get men to participate in antenatal programmes, since it was asking them to do something outside their regular domain of responsibility.”(Turan, 2001) |
| Intervention Design & Characteristics | (Bertrand, 1982); Daniele (2017); Harrington, 2019; Jewkes, 2010; McCarthy 2018a | “The data […] demonstrate that exposure to family planning communications played a key role in the use of contraceptives. … In Guatemala, it was the second most important factor, following closely after educational achievement.” (Bertrand, 1982)  “Men and women consistently identified the potential for FP-related SMS to act as a prompt, or trigger, for couple communication around FP" (Harrington, 2019)  “There was evidence that the combination of the communication/assertiveness skills sessions and the experience of group discussion over several weeks built the participants’ confidence and gave them skills that they used in a range of different settings and with different people. (Jewkes, 2010) |
| Reach | Bertrand, 1982 | “One might expect to find certain selective attention patterns among those groups for which family planning messages might have the greatest salience (women who are married or in consensual union, and well along in the reproductive cycle). While there is some evidence of such patterns, the most marked differences relate to place of residence, education, and employment status. This presents communicators with the classic challenge-how to reach the most isolated and least educated." (Bertrand, 1982) |
| Favourability of contraceptive method | Akhter, 1993; Ghule, 2015 | “In 154 (45.3%) of these cases, early removal was requested because of menstrual problems, the majority of which were due to prolonged heavy bleeding. Most of the discontinuations due to menstrual problems occurred in the second year of the study then decreased steadily thereafter. […] A total of 165 (97.5%) of the 169 women described as favorable their overall experience with the implants.” (Akhter, 1993)  “Husbands and wives highlighted several physiological side effects of the OCP, based on both perceptions more often than experience. Women described nausea and abdominal pain.” (Ghule, 2015) |
| ADVERSE EFFECTS | | |
| Category | Studies Presenting Data | Examples of Data |
| None | Daniele, 2017; Turan, 2001 | “Women and men were asked a general question about whether they felt the intervention had had any impact on their family, both positive and negative. There were no responses indicating an adverse effect.” (Daniele, 2017)  “Though increased dominance by men in 120 decision-making where women have traditionally made the decisions was a risk we were concerned about, when, new mothers and fathers were asked who made household decisions on infant feeding, infant health, post-partum women’s health, and family planning, no differences were found in decision-making among members of the three study groups." (Turan, 2001) |
| Covert Contraceptive Use | Harrington, 2016 | “While concealed contraceptive use represents, on some level, autonomous decision-making, participants did not celebrate covert use as an empowered choice. Rather, covert use was overwhelmingly portrayed as a secretive act and as a source of guilt and fear.[nihms754911.pdf] Page 9: Men were not specifically queried about women’s covert contraceptive use, as there was concern on the part of study investigators that such questions could lead to male suspicion and possible confrontation or violence with female partners. Nonetheless, this domain surfaced spontaneously in interviews with over one-third of male respondents." (Harrington, 2016) |
| Unmet Need | Harrington, 2019 | “However, male resistance to FP in the context of un- equal relationship power dynamics may also be a contributor to women’s unmet need for contraception.” (Harrington, 2019) |

### Appendix 7.0 References for Included Studies

### Appendix 7.1 References for All Included Evaluation Studies (n=127)

- Acharya, D. R. (2014). Measuring the effectiveness of teachings sex education in Nepalese secondary schools: An outcome from a Randomised Controlled Trial (RCT). Retrieved from https://pure.aber.ac.uk/portal/en/theses/measuring-the-effectiveness-of-teachings-sex-education-in-nepalese-secondary-schools(7aed061b-668e-4789-879e-b30ba401b6c6).html
- Agha, S. (2002). A quasi-experimental study to assess the impact of four adolescent sexual health interventions in sub-Saharan Africa. *International Family Planning Perspectives, 28*(2), 67-118. doi: 10.2307/3088237
- Agha, S., Karim, A. M., Balal, A., & Sosler, S. (2007). The impact of a reproductive health franchise on client satisfaction in rural Nepal. *Health Policy Plan, 22*(5), 320-328. doi: 10.1093/heapol/czm025
- Ahmed, S., Ahmed, S., McKaig, C., Begum, N., Mungia, J., Norton, M., & Baqui, A. H. (2015). The Effect of Integrating Family Planning with a Maternal and Newborn Health Program on Postpartum Contraceptive Use and Optimal Birth Spacing in Rural Bangladesh. *Stud Fam Plann, 46*(3), 297-312. doi: 10.1111/j.1728-4465.2015.00031.x
- Ajuwon, A. J., & Brieger, W. R. (2007). Evaluation of a school-based reproductive health education program in rural South Western, Nigeria. *Afr J Reprod Health, 11*(2), 47-59. doi: 10.2307/25549715
- Almeida, N., Teixeira, A., Garcia, J., Martins, N., & Ramalho, C. (2019). Effects of an Educational Intervention on Angolan Adolescents' Knowledge of Human Reproduction: A Quasi-Experimental Study. *Int J Environ Res Public Health, 16*(24). doi: 10.3390/ijerph16245155
- Amatya, R., Akhter, H., McMahan, J., Williamson, N., Gates, D., & Ahmed, Y. (1994). The effect of husband counseling on NORPLANT contraceptive acceptability in Bangladesh. *Contraception, 50*(3), 263-273. doi: 10.1016/0010-7824(94)90072-8
- Amin, S., & Suran, L. (2005). *Program Efforts to Delay Marriage Through Improved Opportunities: Some Evidence from Rural Bangladesh*. New York, NY. Retrieved from http://www.demoscope.ru/weekly/knigi/tours_2005/papers/iussp2005s52255.pdf
- Andrade, H. H., Mello, M. B., Sousa, M. H., Makuch, M. Y., Bertoni, N., & Faundes, A. (2009). Changes in sexual behavior following a sex education program in Brazilian public schools. *Cad Saude Publica, 25*(5), 1168-1176. doi: 10.1590/s0102-311x2009000500023
- Aninanya, G. A., Debpuur, C. Y., Awine, T., Williams, J. E., Hodgson, A., & Howard, N. (2015). Effects of an adolescent sexual and reproductive health intervention on health service usage by young people in northern Ghana: a community-randomised trial. *Plos One, 10*(4), e0125267. doi: 10.1371/journal.pone.0125267
- Atwood, K. A., Kennedy, S. B., Shamblen, S., Taylor, C. H., Quaqua, M., Bee, E. M., . . . Dennis, B. (2012). Reducing sexual risk taking behaviors among adolescents who engage in transactional sex in post-conflict Liberia. *Vulnerable Child Youth Stud, 7*(1), 55-65. doi: 10.1080/17450128.2011.647773
- Bachanas, P., Kidder, D., Medley, A., Pals, S. L., Carpenter, D., Howard, A., . . . Moore, J. (2016). Delivering Prevention Interventions to People Living with HIV in Clinical Care Settings: Results of a Cluster Randomized Trial in Kenya, Namibia, and Tanzania. *AIDS Behav, 20*(9), 2110-2118. doi: 10.1007/s10461-016-1349-2
- Bertrand, J. T., Santiso, R., Linder, S. H., & Pineda, M. A. (1987). Evaluation of a Communications Program to Increase Adoption of Vasectomy in Guatemala. *Studies in Family Planning, 18*(6), 361–370. doi: 10.2307/1966602
- Black, T. R., & Harvey, P. D. (1976). A report on a contraceptive social marketing experiment in rural Kenya. *Stud Fam Plann, 7*(4), 101-108. doi: 10.2307/1965042
- Boti, N., Hussen, S., Shegaze, M., Shibru, S., Shibiru, T., Zerihun, E., . . . Temtime, Z. (2019). Effects of comprehensive sexuality education on the comprehensive knowledge and attitude to condom use among first-year students in Arba Minch University: a quasi-experimental study. *BMC Res Notes, 12*(1), 700. doi: 10.1186/s13104-019-4746-6
- Brieger, W. R., Delano, G. E., Lane, C. G., Oladepo, O., & Oyediran, K. A. (2001). West African Youth Initiative: outcome of a reproductive health education program. *J Adolesc Health, 29*(6), 436-446. doi: 10.1016/s1054-139x(01)00264-6
- Caceres, C. F., Rosasco, A. M., Mandel, J. S., & Hearst, N. (1994). Evaluating a school-based intervention for STD/AIDS prevention in Peru. *J Adolesc Health, 15*(7), 582-591. doi: 10.1016/1054-139x(94)90143-q
- Campero, L., Suarez-Lopez, L., & Cruz-Jimenez, L. (2019). Intervention for the comprehension of menstrual cycle, body function and pregnancy in adolescents from rural context. *Salud pública Méx, 61*(5), 572-581. doi: 10.21149/9988
- Campero, L., Walker, D., Atienzo, E. E., & Gutierrez, J. P. (2011). A quasi-experimental evaluation of parents as sexual health educators resulting in delayed sexual initiation and increased access to condoms. *J Adolesc, 34*(2), 215-223. doi: 10.1016/j.adolescence.2010.05.010
- Chi, X., Hawk, S. T., Winter, S., & Meeus, W. (2015). The effect of comprehensive sexual education program on sexual health knowledge and sexual attitude among college students in Southwest China. *Asia Pac J Public Health, 27*(2), NP2049-2066. doi: 10.1177/1010539513475655
- Chong, A., Gonzalez-Navarro, M., Karlan, D., & Valdivia, M. (2013). *Effectiveness and Spillovers of Online Sex Education: Evidence from a Randomized Evaluationin Colombian Public Schools*. Cambrige, MA. Retrieved from http://www.grade.org.pe/upload/publicaciones/archivo/download/pubs/nberWP_CH_GN_K_V.pdf
- Coffman, D. L., Smith, E. A., Flisher, A. J., & Caldwell, L. L. (2011). Effects of HealthWise South Africa on condom use self-efficacy. *Prev Sci, 12*(2), 162-172. doi: 10.1007/s11121-010-0196-z
- Cowan, F. M., Pascoe, S. J., Langhaug, L. F., Mavhu, W., Chidiya, S., Jaffar, S., . . . Regai Dzive Shiri trial, t. (2010). The Regai Dzive Shiri project: results of a randomized trial of an HIV prevention intervention for youth. *Aids, 24*(16), 2541-2552. doi: 10.1097/QAD.0b013e32833e77c9
- Daniel, E. E., & Nanda, R. (2012). The Effect of Reproductive Health Communication Interventions on Age at Marriage and First Birth in Rural Bihar, India: A retrospective study. Bihar, India: Pathfinder International. Retrieved from http://www2.pathfinder.org/site/DocServer/AOM_paper_-_full_paper_with_covers.pdf?docID=19841
- Daniele, M. A., Ganaba, R., Sarrassat, S., Cousens, S., Rossier, C., Drabo, S., . . . Filippi, V. (2018). Involving male partners in maternity care in Burkina Faso: a randomized controlled trial. *Bull World Health Organ, 96*(7), 450-461. doi: 10.2471/BLT.17.206466
- Debpuur, C., Phillips, J. F., Jackson, E. F., Nazzar, A., Ngom, P., & Binka, F. N. (2002). The impact of the Navrongo Project on contraceptive knowledge and use, reproductive preferences, and fertility. *Stud Fam Plann, 33*(2), 141-164. doi: 10.1111/j.1728-4465.2002.00141.x
- Decat, P. (2015). Addressing the unmet contraceptive need of adolescents and unmarried youth: Act or Interact Learning from comprehensive interventions in China and Latin America. Gent, Belgium.
- Decat, P., De, M., & Jaruseviciene, L. (2014). Abstracts of Free Communications. *The European Journal of Contraception & Reproductive Health Care, 19*(sup1), S67-S90. doi: 10.3109/13625187.2014.894779.10
- Diop, N., Bathidja, H., Diop, T., Dieng, T., Mane, B., Ramarao, S., . . . Fall, B. (2004). *Improving the reproductive health of adolescents in Senegal*. Washington, DC:
- Doyle, A. M., Weiss, H. A., Maganja, K., Kapiga, S., McCormack, S., Watson-Jones, D., . . . Ross, D. A. (2011). The long-term impact of the MEMA kwa Vijana adolescent sexual and reproductive health intervention: effect of dose and time since intervention exposure. *Plos One, 6*(9), e24866. doi: 10.1371/journal.pone.0024866
- Doyle, K., Levtov, R. G., Barker, G., Bastian, G. G., Bingenheimer, J. B., Kazimbaya, S., . . . Shattuck, D. (2018). Gender-transformative Bandebereho couples' intervention to promote male engagement in reproductive and maternal health and violence prevention in Rwanda: Findings from a randomized controlled trial. *Plos One, 13*(4), e0192756. doi: 10.1371/journal.pone.0192756
- Eggleston, E., Jackson, J., Rountree, W., & Pan, Z. (2000). Evaluation of a sexuality education program for young adolescents in Jamaica. *Pan American Journal of Public Health, 7*(2), 102-112. doi: 10.1590/s1020-49892000000200006
- El-Khoury, M., Thornton, R., Chatterji, M., Kamhawi, S., Sloane, P., & Halassa, M. (2016). Counseling Women and Couples on Family Planning: A Randomized Study in Jordan. *Stud Fam Plann, 47*(3), 222-238. doi: 10.1111/sifp.69
- Erulkar, A. S., Ettyang, L. I., Onoka, C., Nyagah, F. K., & Muyonga, A. (2004). Behavior change evaluation of a culturally consistent reproductive health program for young Kenyans. *Int Fam Plan Perspect, 30*(2), 58-67. doi: 10.1363/3005804
- Exner, T. M., Mantell, J. E., Adeokun, L. A., Udoh, I. A., Ladipo, O. A., Delano, G. E., . . . Akinpelu, K. (2009). Mobilizing men as partners: the results of an intervention to increase dual protection among Nigerian men. *Health Educ Res, 24*(5), 846-854. doi: 10.1093/her/cyp021
- Firestone, R., Moorsmith, R., James, S., Urey, M., Greifinger, R., Lloyd, D., . . . Sanoe, M. (2016). Intensive Group Learning and On-Site Services to Improve Sexual and Reproductive Health Among Young Adults in Liberia: A Randomized Evaluation of HealthyActions. *Glob Health Sci Pract, 4*(3), 435-451. doi: 10.9745/GHSP-D-16-00074
- Fisek, N. H., & Sumbuloglu, K. (1978). The effects of husband and wife education on family planning in rural Turkey. *Stud Fam Plann, 9*(10-11), 280-285. doi: 10.2307/1965765
- Fitzgerald, A. M., Stanton, B. F., Terreri, N., Shipena, H., Li, X., Kahihuata, J., . . . de Jaeger, A. M. (1999). Use of Western-based HIV risk-reduction interventions targeting adolescents in an African setting. *Journal of Adolescent Health, 25*(1), 52-61. doi: 10.1016/s1054-139x(98)00120-7
- Fleming, P. J., Silverman, J., Ghule, M., Ritter, J., Battala, M., Velhal, G., . . . Raj, A. (2018). Can a Gender Equity and Family Planning Intervention for Men Change Their Gender Ideology? Results from the CHARM Intervention in Rural India. *Stud Fam Plann, 49*(1), 41-56. doi: 10.1111/sifp.12047
- Foreit, J. R., Garate, M. R., Brazzoduro, A., Guillen, F., Herrera, M. C., & Suarez, F. C. (1992). A comparison of the performance of male and female CBD distributors in Peru. *Stud Fam Plann, 23*(1), 58-62. doi: 10.2307/1966828
- Gallegos, E. C., Villarruel, A. M., Loveland-Cherry, C., Ronis, D. L., & Yan Zhou, M. (2008). [Intervention to reduce adolescents sexual risk behaviors: a randomized controlled trial]. *Salud pública Méx, 50*(1), 59-66. doi: 10.1590/s0036-36342008000100012
- Gao, Y., Lu, Z. Z., Shi, R., Sun, X. Y., & Cai, Y. (2001). AIDS and sex education for young people in China. *Reproduction, Fertility, & Development, 13*(7-8), 729-737. doi: 10.1071/rd01082
- Ha, B. T., Jayasuriya, R., & Owen, N. (2005). Increasing male involvement in family planning decision making: trial of a social-cognitive intervention in rural Vietnam. *Health Educ Res, 20*(5), 548-556. doi: 10.1093/her/cyh013
- Hallman, K., & Roca, E. (2011). Siyakha Nentsha: Building economic, health, and social capabilities among highly vulnerable adolescents in KwaZulu-Natal, South Africa. New York, NY:
- Halpern, C. T., Mitchell, E. M., Farhat, T., & Bardsley, P. (2008). Effectiveness of web-based education on Kenyan and Brazilian adolescents' knowledge about HIV/AIDS, abortion law, and emergency contraception: findings from TeenWeb. *Soc Sci Med, 67*(4), 628-637. doi: 10.1016/j.socscimed.2008.05.001
- Harrington Eiizabeth, K. (2017). Evaluation of an mHealth SMS Dialogue Strategy to Meet Women's and Couples' Postpartum Contraceptive Needs in Kenya (Mobile WACh XY): a Randomized Controlled Trial. ProQuest Dissertations Publishing, Ann Arbor. Retrieved from https://search.proquest.com/docview/2021741378?accountid=13374
- Harrington, E. K., Drake, A. L., Matemo, D., Ronen, K., Osoti, A. O., John-Stewart, G., . . . Unger, J. A. (2019). An mHealth SMS intervention on Postpartum Contraceptive Use Among Women and Couples in Kenya: A Randomized Controlled Trial. *Am J Public Health, 109*(6), 934-941. doi: 10.2105/AJPH.2019.305051
- He, D., Cheng, Y. M., Wu, S. Z., Decat, P., Wang, Z. J., Minkauskiene, M., & Moyer, E. (2012). Promoting contraceptive use more effectively among unmarried male migrants in construction sites in China: a pilot intervention trial. *Asia Pac J Public Health, 24*(5), 806-815. doi: 10.1177/1010539511406106
- Jewkes, R., Nduna, M., Levin, J., Jama, N., Dunkle, K., Puren, A., & Duvvury, N. (2008). Impact of stepping stones on incidence of HIV and HSV-2 and sexual behaviour in rural South Africa: cluster randomised controlled trial. *BMJ, 337*, a506. doi: 10.1136/bmj.a506
- Johnson, D., Juras, R., Riley, P., Chatterji, M., Sloane, P., Choi, S. K., & Johns, B. (2017). A randomized controlled trial of the impact of a family planning mHealth service on knowledge and use of contraception. *Contraception, 95*(1), 90-97. doi: 10.1016/j.contraception.2016.07.009
- Kaljee, L. M., Tho le, H., Minh, T. T., Lerdboon, P., Green, M., Riel, R., & Pham, V. (2012). A sexual health program for Vietnamese parents of adolescents: a preliminary report on changes in parental knowledge, communication, and self-efficacy for condom use. *J Assoc Nurses AIDS Care, 23*(6), 555-560. doi: 10.1016/j.jana.2011.11.003
- Kanesathasan, A., Cardinal, L. J., Pearson, E., Das Gupta, S., Mukherjee, S., & Malhotra, A. (2008). *Catalyzing change: Improving youth sexual and reproductive health through DISHA, an integrated program in India*. New Delhi, India: ICRW. Retrieved from https://www.icrw.org/wp-content/uploads/2016/10/Catalyzing-Change-Improving-Youth-Sexual-and-Reproductive-Health-Through-disha-an-Integrated-Program-in-India-DISHA-Report.pdf
- Katz, K. R., West, C. G., Doumbia, F., & Kane, F. (1998). Increasing Access to Family Planning Services in Rural Mali Through Community-Based Distribution. *International Family Planning Perspectives, 24*(3), 104–110. doi: 10.2307/3038206
- Kemigisha, E., Bruce, K., Ivanova, O., Leye, E., Coene, G., Ruzaaza, G. N., . . . Michielsen, K. (2019). Evaluation of a school based comprehensive sexuality education program among very young adolescents in rural Uganda. *BMC Public Health, 19*(1), 1393. doi: 10.1186/s12889-019-7805-y
- Khanm, A. R., & Huber, D. H. (1976). Door-to-Door Distribution Working in Bangladesh. *International Family Planning Digest, 2*(3), 9-11. doi: 10.2307/2948172
- Khatun, M., Mahboob, E. A., & Nazneen, Q. N. (2011). Assessing public-private reproductive health efforts to reach young married couples in rural Bangladesh. *Int Q Community Health Educ, 32*(1), 73-94. doi: 10.2190/IQ.32.1.g
- Kim, Y. M., Kols, A., Nyakauru, R., Marangwanda, C., & Chibatamoto, P. (2001). Promoting Sexual Responsibility among Young People in Zimbabwe. *International Family Planning Perspectives, 27*(1), 11-19. doi: 10.2307/2673800
- Kulathinal, S., Joseph, B., & Saavala, M. (2019). Mobile Helpline and Reversible Contraception: Lessons From a Controlled Before-and-After Study in Rural India. *JMIR Mhealth Uhealth, 7*(8), e12672. doi: 10.2196/12672
- Lemani, C., Tang, J. H., Kopp, D., Phiri, B., Kumvula, C., Chikosi, L., . . . Rosenberg, N. E. (2017). Contraceptive uptake after training community health workers in couples counseling: A cluster randomized trial. *Plos One, 12*(4), e0175879. doi: 10.1371/journal.pone.0175879
- Lou, C. H., Wang, B., Shen, Y., & Gao, E. S. (2004). Effects of a community-based sex education and reproductive health service program on contraceptive use of unmarried youths in Shanghai. *J Adolesc Health, 34*(5), 433-440. doi: 10.1016/j.jadohealth.2003.07.020
- Lou, C. H., Zhao, Q., Gao, E. S., & Shah, I. H. (2006). Can the Internet be used effectively to provide sex education to young people in China? *J Adolesc Health, 39*(5), 720-728. doi: 10.1016/j.jadohealth.2006.04.003
- Luck, M., Jarju, E., Nell, M. D., & George, M. O. (2000). Mobilizing demand for contraception in rural Gambia. *Stud Fam Plann, 31*(4), 325-335. doi: 10.1111/j.1728-4465.2000.00325.x
- Lutalo, T., Kigozi, G., Kimera, E., Serwadda, D., Wawer, M. J., Zabin, L. S., & Gray, R. H. (2010). A randomized community trial of enhanced family planning outreach in Rakai, Uganda. *Stud Fam Plann, 41*(1), 55-60. doi: 10.1111/j.1728-4465.2010.00224.x
- Magnani, R. J., Gaffikin, L., de Aquino, E. M., Seiber, E. E., Almeida, M. C., & Lipovsek, V. (2001). Impact of an integrated adolescent reproductive health program in Brazil. *Stud Fam Plann, 32*(3), 230-243. doi: 10.1111/j.1728-4465.2001.00230.x
- Mantell, J. E., Cooper, D., Exner, T. M., Moodley, J., Hoffman, S., Myer, L., . . . Nywagi, N. (2017). Emtonjeni-A Structural Intervention to Integrate Sexual and Reproductive Health into Public Sector HIV Care in Cape Town, South Africa: Results of a Phase II Study. *AIDS Behav, 21*(3), 905-922. doi: 10.1007/s10461-016-1562-z
- Mantell, J. E., Harrison, A., Hoffman, S., Smit, J. A., Stein, Z. A., & Exner, T. M. (2006). TheMpondombiliProject: Preventing HIV/AIDS and Unintended Pregnancy among Rural South African School-Going Adolescents. *Reproductive Health Matters, 14*(28), 113-122. doi: 10.1016/s0968-8080(06)28269-7
- Mark, K. E., Meinzen-Derr, J., Stephenson, R., Haworth, A., Ahmed, Y., Duncan, D., . . . Allen, S. (2007). Contraception among HIV concordant and discordant couples in Zambia: a randomized controlled trial. *J Womens Health (Larchmt), 16*(8), 1200-1210. doi: 10.1089/jwh.2006.0238
- Martiniuk, A. L., O'Connor, K. S., & King, W. D. (2003). A cluster randomized trial of a sex education programme in Belize, Central America. *Int J Epidemiol, 32*(1), 131-136. doi: 10.1093/ije/dyg014
- Martiniuk, A. L., O'Connor, K. S., & King, W. D. (2003). A cluster randomized trial of a sex education programme in Belize, Central America. *Int J Epidemiol, 32*(1), 131-136. doi: 10.1093/ije/dyg014
- Mathur, S., Mehta, M., & Malhotra, A. (2004). *Youth reproductive health in Nepal: is participation the answer?* Retrieved from https://www.eldis.org/document/A15330
- Mba, C. I., Obi, S. N., & Ozumba, B. C. (2007). The impact of health education on reproductive health knowledge among adolescents in a rural Nigerian community. *J Obstet Gynaecol, 27*(5), 513-517. doi: 10.1080/01443610701478991
- Mbizvo, M. T., Kasule, J., Gupta, V., Rusakaniko, S., Kinoti, S. N., Mpanju-Shumbushu, W., . . . Padayachy, J. (1997). Effects of a randomized health education intervention on aspects of peproductive health knowledge and reported behaviour among adolescents in Zimbabwe. *Social Science & Medicine, 44*(5), 573-577. doi: 10.1016/s0277-9536(96)00204-3
- Mbonye, A. K. (2003). Disease and health seeking patterns among adolescents in Uganda. *Int J Adolesc Med Health, 15*(2), 105-112. doi: 10.1515/ijamh.2003.15.2.105
- McCarthy, O., Ahamed, I., Kulaeva, F., Tokhirov, R., Saibov, S., Vandewiele, M., . . . Free, C. (2018). A randomized controlled trial of an intervention delivered by mobile phone app instant messaging to increase the acceptability of effective contraception among young people in Tajikistan. *Reprod Health, 15*(1), 28. doi: 10.1186/s12978-018-0473-z
- Medina, J. E., Cifuentes, A., Abernathy, J. R., Spieler, J. M., & Wade, M. E. (1980). Comparative evaluation of two methods of natural family planning in Columbia. *Am J Obstet Gynecol, 138*(8), 1142-1147. doi: 10.1016/s0002-9378(16)32781-8
- Ngure, K., Heffron, R., Mugo, N., Irungu, E., Celum, C., & Baeten, J. M. (2009). Successful increase in contraceptive uptake among Kenyan HIV-1-serodiscordant couples enrolled in an HIV-1 prevention trial. *Aids, 23 Suppl 1*, S89-95. doi: 10.1097/01.aids.0000363781.50580.03
- Odeyemi, K. A., & Ibude, B. E. (2011, 2011). *Promoting Male Participation in Family Planning in Rural Nigeria: A Community-based Intervention.* Paper presented at the International Conference on Family Planning, Dakar, Senegal.
- Ojengbede, O., Morhason-Bello, I., Adedokun, B., Becker, S., Oni, G., & Tsui, A. (2009). P740 Psycho-social support in labour, as a catalyst for contraceptive uptake in Nigeria: Preliminary analysis of a randomised controlled trial. *International Journal of Gynecology & Obstetrics, 107*, S623-S624. doi: 10.1016/s0020-7292(09)62231-1
- Onono, M., Guze, M. A., Grossman, D., Steinfeld, R., Bukusi, E. A., Shade, S., . . . Newmann, S. J. (2015). Integrating family planning and HIV services in western Kenya: the impact on HIV-infected patients' knowledge of family planning and male attitudes toward family planning. *AIDS Care, 27*(6), 743-752. doi: 10.1080/09540121.2014.999744
- Oyugi, B., Kioko, U., Kaboro, S. M., Gikonyo, S., Okumu, C., Ogola-Munene, S., . . . Nzioka, C. (2017). Accessibility of long-term family planning methods: a comparison study between Output Based Approach (OBA) clients verses non-OBA clients in the voucher supported facilities in Kenya. *BMC Health Serv Res, 17*(1), 236. doi: 10.1186/s12913-017-2164-9
- Ozcebe, H., & Akin, L. (2003). Effects of peer education on reproductive health knowledge for adolescents living in rural areas of Turkey. *J Adolesc Health, 33*(4), 217-218. doi: 10.1016/s1054-139x(03)00131-9
- Ozgur, S., Ihsan Bozkurt, A., & Ozcirpici, B. (2000). The effects of family planning education provided to different gender groups. *BJOG, 107*(10), 1226-1232. doi: 10.1111/j.1471-0528.2000.tb11611.x
- Pham, V., Nguyen, H., Tho le, H., Minh, T. T., Lerdboon, P., Riel, R., . . . Kaljee, L. M. (2012). Evaluation of three adolescent sexual health programs in ha noi and khanh hoa province, Vietnam. *AIDS Res Treat, 2012*, 986978. doi: 10.1155/2012/986978
- Phillips, J. F., Jackson, E. F., Bawah, A. A., MacLeod, B., Adongo, P., Baynes, C., & Williams, J. (2012). The long-term fertility impact of the Navrongo project in northern Ghana. *Stud Fam Plann, 43*(3), 175-190. doi: 10.1111/j.1728-4465.2012.00316.x
- Pinchoff, J., Boyer, C. B., Nag Chowdhuri, R., Smith, G., Chintu, N., & Ngo, T. D. (2019). The evaluation of the Woman's Condom marketing approach: What value did peer-led interpersonal communication add to the promotion of a new female condom in urban Lusaka? *Plos One, 14*(12), e0225832. doi: 10.1371/journal.pone.0225832
- Pineda, M. A., Bertrand, J. T., Santiso, R., & Guerra, S. (1983). Increasing the effectiveness of community workers through training of spouses: a family planning experiment in Guatemala. *Public Health Reports, 98*(3), 273-277. Retrieved from https://queens.ezp1.qub.ac.uk/login?url=http://ovidsp.ovid.com/ovidweb.cgi?T=JS&CSC=Y&NEWS=N&PAGE=fulltext&D=med2&AN=6867260
- Pitt, M. M., Khandker, S. R., McKernan, S. M., & Abdul Latif, M. (1999). Credit programs for the poor and reproductive behavior in low-income countries: are the reported causal relationships the result of heterogeneity bias? *Demography, 36*(1), 1-21. doi: 10.2307/2648131
- Pivatti, A. S. A., Osis, M., & de Moraes Lopes, M. H. B. (2019). "The use of educational strategies for promotion of knowledge, attitudes and contraceptive practices among teenagers - A randomized clinical trial". *Nurse Educ Today, 72*, 18-26. doi: 10.1016/j.nedt.2018.10.005
- Prasertsawat, P. O., & Koktatong, U. (2002). Hands-on is better than look-on: condom use. *Journal of the Medical Association of Thailand, 85*(12), 1309-1313. Retrieved from https://pubmed.ncbi.nlm.nih.gov/12678169/
- Raj, A., Ghule, M., Ritter, J., Battala, M., Gajanan, V., Nair, S., . . . Saggurti, N. (2016). Cluster Randomized Controlled Trial Evaluation of a Gender Equity and Family Planning Intervention for Married Men and Couples in Rural India. *Plos One, 11*(5), e0153190. doi: 10.1371/journal.pone.0153190
- Rogers, E. M., Vaughan, P. W., Swalehe, R. M., Rao, N., Svenkerud, P., & Sood, S. (1999). Effects of an entertainment-education radio soap opera on family planning behavior in Tanzania. *Stud Fam Plann, 30*(3), 193-211. doi: 10.1111/j.1728-4465.1999.00193.x
- Ross, D. A., Changalucha, J., Obasi, A. I., Todd, J., Plummer, M. L., Cleophas-Mazige, B., . . . Hayes, R. J. (2007). Biological and behavioural impact of an adolescent sexual health intervention in Tanzania: a community-randomized trial. *Aids, 21*(14), 1943-1955. doi: 10.1097/QAD.0b013e3282ed3cf5
- Rusakaniko, S., Mbizvo, M. T., Kasule, J., Gupta, V., Kinoti, S. N., Mpanju-Shumbushu, W., . . . Padayachy, J. (1997). Trends in reproductive health knowledge following a health education intervention among adolescents in Zimbabwe. *Central African journal of medicine, 43*(1), 1-6. Retrieved from https://queens.ezp1.qub.ac.uk/login?url=http://ovidsp.ovid.com/ovidweb.cgi?T=JS&CSC=Y&NEWS=N&PAGE=fulltext&D=med4&AN=9185371
- Sahip, Y., & Turan, J. M. (2007). Education for expectant fathers in workplaces in Turkey. *J Biosoc Sci, 39*(6), 843-860. doi: 10.1017/S0021932007002088
- Schuler, S. R. (2012). Interactive workshops to promote gender equity and family planning in rural communities of Tanzania: Results of a field test. Retrieved from internal-pdf://2570948697/Schuler-2012.pdf
- Schuler, S. R., Nanda, G., Ramirez, L. F., & Chen, M. (2015). Interactive Workshops to Promote Gender Equity and Family Planning in Rural Communities of Guatemala: Results of a Community Randomized Study. *J Biosoc Sci, 47*(5), 667-686. doi: 10.1017/S0021932014000418
- Sebastian, M. P., Khan, M. E., & Roychowdhury, S. (2010). Promoting healthy spacing between pregnancies in India: need for differential education campaigns. *Patient Educ Couns, 81*(3), 395-401. doi: 10.1016/j.pec.2010.10.019
- Shattuck, D., Kerner, B., Gilles, K., Hartmann, M., Ng'ombe, T., & Guest, G. (2011). Encouraging contraceptive uptake by motivating men to communicate about family planning: the Malawi Male Motivator project. *Am J Public Health, 101*(6), 1089-1095. doi: 10.2105/AJPH.2010.300091
- Shuey, D. A., Babishangire, B. B., Omiat, S., & Bagarukayo, H. (1999). Increased sexual abstinence among in-school adolescents as a result of school health education in Soroti district, Uganda. *Health Educ Res, 14*(3), 411-419. doi: 10.1093/her/14.3.411
- Singh, D., Negin, J., Orach, C. G., & Cumming, R. (2016). Supportive supervision for volunteers to deliver reproductive health education: a cluster randomized trial. *Reprod Health, 13*(1), 126. doi: 10.1186/s12978-016-0244-7
- Speizer, I. S., Tambashe, B. O., & Tegang, S. P. (2001). An evaluation of the "Entre Nous Jeunes" peer-educator program for adolescents in Cameroon. *Stud Fam Plann, 32*(4), 339-351. doi: 10.1111/j.1728-4465.2001.00339.x
- Stephenson, R., Vwalika, B., Greenberg, L., Ahmed, Y., Vwalika, C., Chomba, E., . . . Allen, S. (2011). A randomized controlled trial to promote long-term contraceptive use among HIV-serodiscordant and concordant positive couples in Zambia. *J Womens Health (Larchmt), 20*(4), 567-574. doi: 10.1089/jwh.2010.2113
- Subramanian, L., Simon, C., & Daniel, E. E. (2018). Increasing Contraceptive Use Among Young Married Couples in Bihar, India: Evidence From a Decade of Implementation of the PRACHAR Project. *Glob Health Sci Pract, 6*(2), 330-344. doi: 10.9745/GHSP-D-17-00440
- Tang, S., Tian, L., Cao, W. W., Zhang, K., Detels, R., & Li, V. C. (2009). Improving reproductive health knowledge in rural China--a web-based strategy. *J Health Commun, 14*(7), 690-714. doi: 10.1080/10810730903204270
- Taylor, M., Jinabhai, C., Dlamini, S., Sathiparsad, R., Eggers, M. S., & De Vries, H. (2014). Effects of a teenage pregnancy prevention program in KwaZulu-Natal, South Africa. *Health Care Women Int, 35*(7-9), 845-858. doi: 10.1080/07399332.2014.910216
- Terefe, A., & Larson, C. P. (1993). Modern contraception use in Ethiopia: does involving husbands make a difference? *Am J Public Health, 83*(11), 1567-1571. doi: 10.2105/ajph.83.11.1567
- Thato, R., Jenkins, R. A., & Dusitsin, N. (2008). Effects of the culturally-sensitive comprehensive sex education programme among Thai secondary school students. *J Adv Nurs, 62*(4), 457-469. doi: 10.1111/j.1365-2648.2008.04609.x
- Tilahun, T., Coene, G., Temmerman, M., & Degomme, O. (2015). Couple based family planning education: changes in male involvement and contraceptive use among married couples in Jimma Zone, Ethiopia. *BMC Public Health, 15*(1), 682. doi: 10.1186/s12889-015-2057-y
- Timol, F., Vawda, M. Y., Bhana, A., Moolman, B., Makoae, M., & Swartz, S. (2016). Addressing adolescents' risk and protective factors related to risky behaviours: Findings from a school-based peer-education evaluation in the Western Cape. *Sahara J, 13*(1), 197-207. doi: 10.1080/17290376.2016.1241188
- Torres, P., Walker, D. M., Gutierez, J. P., & Bertozzi, S. M. (2006). [A novel school-based strategy for the prevention of HIV/AIDS, sexually transmitted disease (STDs), and teen pregnancies]. *Salud pública Méx, 48*(4), 308-316. doi: 10.1590/s0036-36342006000400005
- Townsend, J. W., Diaz de May, E., Sepulveda, Y., Santos de Garza, Y., & Rosenhouse, S. (1987). Sex education and family planning services for young adults: alternative urban strategies in Mexico. *Stud Fam Plann, 18*(2), 103-108. doi: 10.2307/1966701
- Tu, X., Lou, C., Gao, E., & Shah, I. H. (2008). Long-term effects of a community-based program on contraceptive use among sexually active unmarried youth in Shanghai, China. *J Adolesc Health, 42*(3), 249-258. doi: 10.1016/j.jadohealth.2007.08.028
- Tuladhar, J. M., & Stoeckel, J. (1982). The relative impacts of vertical and integrated FP/MCH programs in rural Nepal. *Stud Fam Plann, 13*(10), 275-286. doi: 10.2307/1965850
- Turan Janet, M. (1997). The design and evaluation of an intervention to promote postpartum health in Istanbul: Does including fathers make a difference? (PhD). Johns Hopkins University, Maryland United States. Retrieved from https://queens.ezp1.qub.ac.uk/login?url=https://www.proquest.com/dissertations-theses/design-evaluation-intervention-promote-postpartum/docview/304346206/se-2?accountid=13374
- UERD. (1999). An evaluation of the impact of community health field stations in Burkina Faso. *Africa's population and development bulletin*, 28. Retrieved from https://repository.uneca.org/bitstream/handle/10855/4411/bib.%2031598_I.pdf?sequence=1&isAllowed=y
- Van, H., Van, K., Tinh, H. Q., Thuy, T. H., & Hung, N. P. (2019). Extra-curricular activities improved reproductive health knowledge of ethnic minority high school students in Vietnam. *Journal of family and reproductive health, 13*(2), 80-84. Retrieved from http://jfrh.tums.ac.ir/index.php/jfrh/article/download/1068/484 https://queens.ezp1.qub.ac.uk/login?url=http://ovidsp.ovid.com/ovidweb.cgi?T=JS&CSC=Y&NEWS=N&PAGE=fulltext&D=emexa&AN=2003254159
- Van Rossem, R., & Meekers, D. (1999). *An Evaluation of the Effectiveness of Targeted Social Marketing to Promote Adolescent Reproductive Health in Guinea*. Washington, DC. Retrieved from https://www.psi.org/wp-content/uploads/1999/10/WP23.pdf
- Vernon, R., & Dura, M. (2004). Improving the reproductive health of youth in Mexico. Washington, DC:
- Villarruel, A. M., Zhou, Y., Gallegos, E. C., & Ronis, D. L. (2010). Examining long-term effects of Cuidate-a sexual risk reduction program in Mexican youth. *Pan American Journal of Public Health, 27*(5), 345-351. doi: 10.1590/s1020-49892010000500004
- Walker, D., Gutierrez, J. P., Torres, P., & Bertozzi, S. M. (2006). HIV prevention in Mexican schools: prospective randomised evaluation of intervention. *BMJ, 332*(7551), 1189-1194. doi: 10.1136/bmj.38796.457407.80
- Wall, K. M., Kilembe, W., Nizam, A., Vwalika, C., Kautzman, M., Chomba, E., . . . Allen, S. (2012). Promotion of couples' voluntary HIV counselling and testing in Lusaka, Zambia by influence network leaders and agents. *BMJ Open, 2*(5). doi: 10.1136/bmjopen-2012-001171
- Wang, B., Hertog, S., Meier, A., Lou, C., & Gao, E. (2005). The potential of comprehensive sex education in China: findings from suburban Shanghai. *Int Fam Plan Perspect, 31*(2), 63-72. doi: 10.1363/3106305
- Wang, C. C., Vittinghoff, E., Hua, L. S., Yun, W. H., & Rong, Z. M. (1998). Reducing pregnancy and induced abortion rates in China: family planning with husband participation. *Am J Public Health, 88*(4), 646-648. doi: 10.2105/ajph.88.4.646
- World Health, O. (1987). A prospective multicenter trial of the ovulation method of natural family planning. V. Psychosexual aspects**Supported by the Special Programme of Research Development and Research Training in Human Reproduction, World Health Organization, Geneva, Switzerland. *Fertility and Sterility, 47*(5), 765-772. doi: 10.1016/s0015-0282(16)59162-6
- Yang, J. M., Bang, S., Kim, M. H., & Lee, M. G. (1965). Fertility and family planning in rural Korea. *Population Studies, 18*(3), 237-250. doi: 10.1080/00324728.1965.10405451
- Zhang, W.-H., Li, J., Che, Y., Wu, S., Qian, X., Dong, X., . . . Temmerman, M. (2017). Effects of post-abortion family planning services on preventing unintended pregnancy and repeat abortion (INPAC): a cluster randomised controlled trial in 30 Chinese provinces. *The Lancet, 390*(SPEC.ISS 1), 29‐. doi: 10.1016/s0140-6736(17)33167-7
- Zhu, J. L., Zhang, W. H., Cheng, Y., Xu, J., Xu, X., Gibson, D., . . . Temmerman, M. (2009). Impact of post-abortion family planning services on contraceptive use and abortion rate among young women in China: a cluster randomised trial. *Eur J Contracept Reprod Health Care, 14*(1), 46-54. doi: 10.1080/13625180802512994

### Appendix 7.2 References for Male Engagement Studies (n=44)

- Ahmed, S., Ahmed, S., McKaig, C., Begum, N., Mungia, J., Norton, M., & Baqui, A. H. (2015). The Effect of Integrating Family Planning with a Maternal and Newborn Health Program on Postpartum Contraceptive Use and Optimal Birth Spacing in Rural Bangladesh. *Stud Fam Plann, 46*(3), 297-312. doi: 10.1111/j.1728-4465.2015.00031.x
- Amatya, R., Akhter, H., McMahan, J., Williamson, N., Gates, D., & Ahmed, Y. (1994). The effect of husband counseling on NORPLANT contraceptive acceptability in Bangladesh. *Contraception, 50*(3), 263-273. doi: 10.1016/0010-7824(94)90072-8
- Bertrand, J. T., Santiso, R., Linder, S. H., & Pineda, M. A. (1987). Evaluation of a Communications Program to Increase Adoption of Vasectomy in Guatemala. *Studies in Family Planning, 18*(6), 361–370. doi: 10.2307/1966602
- Daniel, E. E., & Nanda, R. (2012). The Effect of Reproductive Health Communication Interventions on Age at Marriage and First Birth in Rural Bihar, India: A retrospective study. Bihar, India: Pathfinder International. Retrieved from http://www2.pathfinder.org/site/DocServer/AOM_paper_-_full_paper_with_covers.pdf?docID=19841
- Daniele, M. A., Ganaba, R., Sarrassat, S., Cousens, S., Rossier, C., Drabo, S., . . . Filippi, V. (2018). Involving male partners in maternity care in Burkina Faso: a randomized controlled trial. *Bull World Health Organ, 96*(7), 450-461. doi: 10.2471/BLT.17.206466
- Doyle, K., Levtov, R. G., Barker, G., Bastian, G. G., Bingenheimer, J. B., Kazimbaya, S., . . . Shattuck, D. (2018). Gender-transformative Bandebereho couples' intervention to promote male engagement in reproductive and maternal health and violence prevention in Rwanda: Findings from a randomized controlled trial. *Plos One, 13*(4), e0192756. doi: 10.1371/journal.pone.0192756
- El-Khoury, M., Thornton, R., Chatterji, M., Kamhawi, S., Sloane, P., & Halassa, M. (2016). Counseling Women and Couples on Family Planning: A Randomized Study in Jordan. *Stud Fam Plann, 47*(3), 222-238. doi: 10.1111/sifp.69
- Exner, T. M., Mantell, J. E., Adeokun, L. A., Udoh, I. A., Ladipo, O. A., Delano, G. E., . . . Akinpelu, K. (2009). Mobilizing men as partners: the results of an intervention to increase dual protection among Nigerian men. *Health Educ Res, 24*(5), 846-854. doi: 10.1093/her/cyp021
- Fisek, N. H., & Sumbuloglu, K. (1978). The effects of husband and wife education on family planning in rural Turkey. *Stud Fam Plann, 9*(10-11), 280-285. doi: 10.2307/1965765
- Fleming, P. J., Silverman, J., Ghule, M., Ritter, J., Battala, M., Velhal, G., . . . Raj, A. (2018). Can a Gender Equity and Family Planning Intervention for Men Change Their Gender Ideology? Results from the CHARM Intervention in Rural India. *Stud Fam Plann, 49*(1), 41-56. doi: 10.1111/sifp.12047
- Foreit, J. R., Garate, M. R., Brazzoduro, A., Guillen, F., Herrera, M. C., & Suarez, F. C. (1992). A comparison of the performance of male and female CBD distributors in Peru. *Stud Fam Plann, 23*(1), 58-62. doi: 10.2307/1966828
- Ha, B. T., Jayasuriya, R., & Owen, N. (2005). Increasing male involvement in family planning decision making: trial of a social-cognitive intervention in rural Vietnam. *Health Educ Res, 20*(5), 548-556. doi: 10.1093/her/cyh013
- Hallman, K., & Roca, E. (2011). Siyakha Nentsha: Building economic, health, and social capabilities among highly vulnerable adolescents in KwaZulu-Natal, South Africa. New York, NY:
- Harrington Eiizabeth, K. (2017). Evaluation of an mHealth SMS Dialogue Strategy to Meet Women's and Couples' Postpartum Contraceptive Needs in Kenya (Mobile WACh XY): a Randomized Controlled Trial. ProQuest Dissertations Publishing, Ann Arbor. Retrieved from https://search.proquest.com/docview/2021741378?accountid=13374
- Harrington, E. K., Drake, A. L., Matemo, D., Ronen, K., Osoti, A. O., John-Stewart, G., . . . Unger, J. A. (2019). An mHealth SMS intervention on Postpartum Contraceptive Use Among Women and Couples in Kenya: A Randomized Controlled Trial. *Am J Public Health, 109*(6), 934-941. doi: 10.2105/AJPH.2019.305051
- Jewkes, R., Nduna, M., Levin, J., Jama, N., Dunkle, K., Puren, A., & Duvvury, N. (2008). Impact of stepping stones on incidence of HIV and HSV-2 and sexual behaviour in rural South Africa: cluster randomised controlled trial. *BMJ, 337*, a506. doi: 10.1136/bmj.a506
- Kanesathasan, A., Cardinal, L. J., Pearson, E., Das Gupta, S., Mukherjee, S., & Malhotra, A. (2008). *Catalyzing change: Improving youth sexual and reproductive health through DISHA, an integrated program in India*. New Delhi, India: ICRW. Retrieved from https://www.icrw.org/wp-content/uploads/2016/10/Catalyzing-Change-Improving-Youth-Sexual-and-Reproductive-Health-Through-disha-an-Integrated-Program-in-India-DISHA-Report.pdf
- Lemani, C., Tang, J. H., Kopp, D., Phiri, B., Kumvula, C., Chikosi, L., . . . Rosenberg, N. E. (2017). Contraceptive uptake after training community health workers in couples counseling: A cluster randomized trial. *Plos One, 12*(4), e0175879. doi: 10.1371/journal.pone.0175879
- Mantell, J. E., Cooper, D., Exner, T. M., Moodley, J., Hoffman, S., Myer, L., . . . Nywagi, N. (2017). Emtonjeni-A Structural Intervention to Integrate Sexual and Reproductive Health into Public Sector HIV Care in Cape Town, South Africa: Results of a Phase II Study. *AIDS Behav, 21*(3), 905-922. doi: 10.1007/s10461-016-1562-z
- Mathur, S., Mehta, M., & Malhotra, A. (2004). *Youth reproductive health in Nepal: is participation the answer?* Retrieved from https://www.eldis.org/document/A15330
- McCarthy, O., Ahamed, I., Kulaeva, F., Tokhirov, R., Saibov, S., Vandewiele, M., . . . Free, C. (2018). A randomized controlled trial of an intervention delivered by mobile phone app instant messaging to increase the acceptability of effective contraception among young people in Tajikistan. *Reprod Health, 15*(1), 28. doi: 10.1186/s12978-018-0473-z
- Ngure, K., Heffron, R., Mugo, N., Irungu, E., Celum, C., & Baeten, J. M. (2009). Successful increase in contraceptive uptake among Kenyan HIV-1-serodiscordant couples enrolled in an HIV-1 prevention trial. *Aids, 23 Suppl 1*, S89-95. doi: 10.1097/01.aids.0000363781.50580.03
- Odeyemi, K. A., & Ibude, B. E. (2011, 2011). *Promoting Male Participation in Family Planning in Rural Nigeria: A Community-based Intervention.* Paper presented at the International Conference on Family Planning, Dakar, Senegal.
- Ojengbede, O., Morhason-Bello, I., Adedokun, B., Becker, S., Oni, G., & Tsui, A. (2009). P740 Psycho-social support in labour, as a catalyst for contraceptive uptake in Nigeria: Preliminary analysis of a randomised controlled trial. *International Journal of Gynecology & Obstetrics, 107*, S623-S624. doi: 10.1016/s0020-7292(09)62231-1
- Onono, M., Guze, M. A., Grossman, D., Steinfeld, R., Bukusi, E. A., Shade, S., . . . Newmann, S. J. (2015). Integrating family planning and HIV services in western Kenya: the impact on HIV-infected patients' knowledge of family planning and male attitudes toward family planning. *AIDS Care, 27*(6), 743-752. doi: 10.1080/09540121.2014.999744
- Ozgur, S., Ihsan Bozkurt, A., & Ozcirpici, B. (2000). The effects of family planning education provided to different gender groups. *BJOG, 107*(10), 1226-1232. doi: 10.1111/j.1471-0528.2000.tb11611.x
- Pham, V., Nguyen, H., Tho le, H., Minh, T. T., Lerdboon, P., Riel, R., . . . Kaljee, L. M. (2012). Evaluation of three adolescent sexual health programs in ha noi and khanh hoa province, Vietnam. *AIDS Res Treat, 2012*, 986978. doi: 10.1155/2012/986978
- Phillips, J. F., Jackson, E. F., Bawah, A. A., MacLeod, B., Adongo, P., Baynes, C., & Williams, J. (2012). The long-term fertility impact of the Navrongo project in northern Ghana. *Stud Fam Plann, 43*(3), 175-190. doi: 10.1111/j.1728-4465.2012.00316.x
- Raj, A., Ghule, M., Ritter, J., Battala, M., Gajanan, V., Nair, S., . . . Saggurti, N. (2016). Cluster Randomized Controlled Trial Evaluation of a Gender Equity and Family Planning Intervention for Married Men and Couples in Rural India. *Plos One, 11*(5), e0153190. doi: 10.1371/journal.pone.0153190
- Sahip, Y., & Turan, J. M. (2007). Education for expectant fathers in workplaces in Turkey. *J Biosoc Sci, 39*(6), 843-860. doi: 10.1017/S0021932007002088
- Schuler, S. R. (2012). Interactive workshops to promote gender equity and family planning in rural communities of Tanzania: Results of a field test. Retrieved from internal-pdf://2570948697/Schuler-2012.pdf
- Schuler, S. R., Nanda, G., Ramirez, L. F., & Chen, M. (2015). Interactive Workshops to Promote Gender Equity and Family Planning in Rural Communities of Guatemala: Results of a Community Randomized Study. *J Biosoc Sci, 47*(5), 667-686. doi: 10.1017/S0021932014000418
- Sebastian, M. P., Khan, M. E., & Roychowdhury, S. (2010). Promoting healthy spacing between pregnancies in India: need for differential education campaigns. *Patient Educ Couns, 81*(3), 395-401. doi: 10.1016/j.pec.2010.10.019
- Shattuck, D., Kerner, B., Gilles, K., Hartmann, M., Ng'ombe, T., & Guest, G. (2011). Encouraging contraceptive uptake by motivating men to communicate about family planning: the Malawi Male Motivator project. *Am J Public Health, 101*(6), 1089-1095. doi: 10.2105/AJPH.2010.300091
- Singh, D., Negin, J., Orach, C. G., & Cumming, R. (2016). Supportive supervision for volunteers to deliver reproductive health education: a cluster randomized trial. *Reprod Health, 13*(1), 126. doi: 10.1186/s12978-016-0244-7
- Subramanian, L., Simon, C., & Daniel, E. E. (2018). Increasing Contraceptive Use Among Young Married Couples in Bihar, India: Evidence From a Decade of Implementation of the PRACHAR Project. *Glob Health Sci Pract, 6*(2), 330-344. doi: 10.9745/GHSP-D-17-00440
- Terefe, A., & Larson, C. P. (1993). Modern contraception use in Ethiopia: does involving husbands make a difference? *Am J Public Health, 83*(11), 1567-1571. doi: 10.2105/ajph.83.11.1567
- Tilahun, T., Coene, G., Temmerman, M., & Degomme, O. (2015). Couple based family planning education: changes in male involvement and contraceptive use among married couples in Jimma Zone, Ethiopia. *BMC Public Health, 15*(1), 682. doi: 10.1186/s12889-015-2057-y
- Turan Janet, M. (1997). The design and evaluation of an intervention to promote postpartum health in Istanbul: Does including fathers make a difference? (PhD). Johns Hopkins University, Maryland United States. Retrieved from https://queens.ezp1.qub.ac.uk/login?url=https://www.proquest.com/dissertations-theses/design-evaluation-intervention-promote-postpartum/docview/304346206/se-2?accountid=13374
- Vernon, R., & Dura, M. (2004). Improving the reproductive health of youth in Mexico. Washington, DC:
- Wang, C. C., Vittinghoff, E., Hua, L. S., Yun, W. H., & Rong, Z. M. (1998). Reducing pregnancy and induced abortion rates in China: family planning with husband participation. *Am J Public Health, 88*(4), 646-648. doi: 10.2105/ajph.88.4.646
- Yang, J. M., Bang, S., Kim, M. H., & Lee, M. G. (1965). Fertility and family planning in rural Korea. *Population Studies, 18*(3), 237-250. doi: 10.1080/00324728.1965.10405451
- Zhang, W.-H., Li, J., Che, Y., Wu, S., Qian, X., Dong, X., . . . Temmerman, M. (2017). Effects of post-abortion family planning services on preventing unintended pregnancy and repeat abortion (INPAC): a cluster randomised controlled trial in 30 Chinese provinces. *The Lancet, 390*(SPEC.ISS 1), 29‐. doi: 10.1016/s0140-6736(17)33167-7
- Zhu, J. L., Zhang, W. H., Cheng, Y., Xu, J., Xu, X., Gibson, D., . . . Temmerman, M. (2009). Impact of post-abortion family planning services on contraceptive use and abortion rate among young women in China: a cluster randomised trial. *Eur J Contracept Reprod Health Care, 14*(1), 46-54. doi: 10.1080/13625180802512994

### Appendix 7.3 References for all Included Connected papers (n=23)

- Ahmed, S., Norton, M., Williams, E., Ahmed, S., Shah, R., Begum, N., . . . Baqui, A. H. (2013). Operations research to add postpartum family planning to maternal and neonatal health to improve birth spacing in Sylhet District, Bangladesh. *Glob Health Sci Pract, 1*(2), 262-276. doi: 10.9745/GHSP-D-13-00002
- Akhter, H., Dunson, T. R., Amatya, R. N., Begum, K., Chowdhury, T., Dighe, N., . . . Rahman, S. (1993). A five-year clinical evaluation of NORPLANTR contraceptive subdermal implants in Bangladeshi acceptors. *Contraception, 47*(6), 569-582. doi: 10.1016/0010-7824(93)90024-2
- Baqui, A. H., Ahmed, S., Begum, N., Khanam, R., Mohan, D., Harrison, M., . . . Projahnmo Study Group in, B. (2018). Impact of integrating a postpartum family planning program into a community-based maternal and newborn health program on birth spacing and preterm birth in rural Bangladesh. *J Glob Health, 8*(2), 020406. doi: 10.7189/jogh.08.020406
- Bertrand, J. T., Santiso, R., Cisneros, R. J., Mascarin, F., & Morris, L. (1982). Family planning communications and contraceptive use in Guatemala, El Salvador, and Panama. *Studies in Family Planning, 13*(6-7), 190-199. Retrieved from https://queens.ezp1.qub.ac.uk/login?url=http://ovidsp.ovid.com/ovidweb.cgi?T=JS&CSC=Y&NEWS=N&PAGE=fulltext&D=med2&AN=6981231
- Cooper, C. M., Ahmed, S., Winch, P. J., Pfitzer, A., McKaig, C., & Baqui, A. H. (2014). Findings from the use of a narrative story and leaflet to influence shifts along the behavior change continuum toward postpartum contraceptive uptake in Sylhet District, Bangladesh. *Patient Educ Couns, 97*(3), 376-382. doi: 10.1016/j.pec.2014.09.007
- Daniele, M. (2017). Involving Men in Maternity Care in Burkina Faso:An Intervention Study.
- Doyle, K., Kato-Wallace, J., Kazimbaya, S., & Barker, G. (2014). Transforming gender roles in domestic and caregiving work: preliminary findings from engaging fathers in maternal, newborn, and child health in Rwanda. *Gender & Development, 22*(3), 515-531. doi: 10.1080/13552074.2014.963326
- Ghule, M., Raj, A., Palaye, P., Dasgupta, A., Nair, S., Saggurti, N., . . . Balaiah, D. (2015). Barriers to use contraceptive methods among rural young married couples in Maharashtra, India: Qualitative findings. *Asian J Res Soc Sci Humanit, 5*(6), 18-33. doi: 10.5958/2249-7315.2015.00132.X
- Harrington Eiizabeth, K. (2017). Evaluation of an mHealth SMS Dialogue Strategy to Meet Women's and Couples' Postpartum Contraceptive Needs in Kenya (Mobile WACh XY): a Randomized Controlled Trial. ProQuest Dissertations Publishing, Ann Arbor. Retrieved from https://search.proquest.com/docview/2021741378?accountid=13374
- Harrington, E. K., Drake, A. L., Matemo, D., Perrier, T., Osoti, A., John-Stewart, G., . . . Unger, J. A. (2017). Experience including men in a novel short message service (SMS) approach to improve postpartum family planning education and counseling in Kenya. *Contraception, 96*(4), 301‐. doi: 10.1016/j.contraception.2017.07.143
- Harrington, E. K., Dworkin, S., Withers, M., Onono, M., Kwena, Z., & Newmann, S. J. (2016). Gendered power dynamics and women's negotiation of family planning in a high HIV prevalence setting: a qualitative study of couples in western Kenya. *Cult Health Sex, 18*(4), 453-469. doi: 10.1080/13691058.2015.1091507
- Harrington, E. K., McCoy, E. E., Drake, A. L., Matemo, D., John-Stewart, G., Kinuthia, J., & Unger, J. A. (2019). Engaging men in an mHealth approach to support postpartum family planning among couples in Kenya: a qualitative study. *Reprod Health, 16*(1), 17. doi: 10.1186/s12978-019-0669-x
- Hartmann, M., Gilles, K., Shattuck, D., Kerner, B., & Guest, G. (2012). Changes in couples' communication as a result of a male-involvement family planning intervention. *J Health Commun, 17*(7), 802-819. doi: 10.1080/10810730.2011.650825
- Jewkes, R., Wood, K., & Duvvury, N. (2010). 'I woke up after I joined Stepping Stones': meanings of an HIV behavioural intervention in rural South African young people's lives. *Health Educ Res, 25*(6), 1074-1084. doi: 10.1093/her/cyq062
- Khan, M. E., Mary Philip Sebastian, U. S. R. I. K. K., & Shahid, A. (2008). *Promoting healthy timing and spacing of births in India through a community-based approach*. Washington, DC. Retrieved from https://knowledgecommons.popcouncil.org/departments_sbsr-rh/417/
- Mantell, J. E., Exner, T. M., Cooper, D., Bai, D., Leu, C. S., Hoffman, S., . . . Stein, Z. A. (2014). Pregnancy intent among a sample of recently diagnosed HIV-positive women and men practicing unprotected sex in Cape Town, South Africa. *J Acquir Immune Defic Syndr, 67 Suppl 4*, S202-209. doi: 10.1097/QAI.0000000000000369
- McCarthy, O., Kulaeva, F., Tohirov, R., Saibov, S., Vandewiele, M., Standaert, S., . . . et al. (2018). Book of Abstracts: The 15th Congress of the European Society of Contraception and Reproductive Health. *Eur J Contracept Reprod Health Care, 23*(sup1), 1-143. doi: 10.1080/13625187.2018.1442911
- McCarthy, O. L. (2019). *Changing young people's attitudes towards effective contraception using mobile phone messaging.* Retrieved from https://researchonline.lshtm.ac.uk/id/eprint/4653004/1/2019_EPH_PhD_McCarthy_O.pdf
- McCarthy, O. L., Wazwaz, O., Osorio Calderon, V., Jado, I., Saibov, S., Stavridis, A., . . . Free, C. (2018). Development of an intervention delivered by mobile phone aimed at decreasing unintended pregnancy among young people in three lower middle income countries. *BMC Public Health, 18*(1), 576. doi: 10.1186/s12889-018-5477-7
- Nair, S., Dixit, A., Ghule, M., Battala, M., Gajanan, V., Dasgupta, A., . . . Raj, A. (2019). Health care providers' perspectives on delivering gender equity focused family planning program for young married couples in a cluster randomized controlled trial in rural Maharashtra, India. *Gates Open Res, 3*, 1508. doi: 10.12688/gatesopenres.13026.1
- Ngure, K., Ng’ang’a, Z., Kimani, V., Khamadi, S., Onchiri, F., Irungu, E., . . . Mugo, N. (2012). Correlates of contraceptive use among HIV discordant couples in Kenya. *African Population Studies, 26*(1). doi: 10.11564/26-1-221
- Rahman, M., & Daniel, E. E. (2010). Reproductive Health Communication Model That Helps Improve Young Women’s Reproductive Life and Reduce Population Growth: The Case of PRACHAR from Bihar, India. Retrieved from https://healtheducationresources.unesco.org/sites/default/files/resources/bie_prachar_impact_-
- Ross, J. A., & Bang, S. (1966). The AID computer programme, used to predict adoption of family planning in Koyang. *Popul Stud (Camb), 20*(1), 61-75. doi: 10.1080/00324728.1966.10406084
- Singh, D., Cumming, R., Mohajer, N., & Negin, J. (2016). Motivation of Community Health Volunteers in rural Uganda: the interconnectedness of knowledge, relationship and action. *Public Health, 136*, 166-171. doi: 10.1016/j.puhe.2016.01.010
